# Secreted inhibitors drive the loss of regeneration competence in *Xenopus* limbs

**DOI:** 10.1242/dev.199158

**Published:** 2021-06-09

**Authors:** Can Aztekin, Tom W. Hiscock, John Gurdon, Jerome Jullien, John Marioni, Benjamin David Simons

**Affiliations:** 1 Wellcome Trust/Cancer Research UK Gurdon Institute, University of Cambridge, Cambridge CB2 1QN, UK; 2 Department of Zoology, University of Cambridge, Cambridge CB2 3EJ, UK; 3 Cancer Research UK Cambridge Institute, University of Cambridge, Cambridge CB2 0RE, UK; 4 Institute of Medical Sciences, Foresterhill Health Campus, University of Aberdeen, Aberdeen AB25 2ZD, UK; 5 Nantes Université, Inserm, Centre de Recherche en Transplantation et Immunologie, UMR 1064, ITUN, F-44000 Nantes, France; 6 EMBL–European Bioinformatics Institute, Wellcome Genome Campus, Cambridge CB10 1SA, UK; 7 Wellcome Sanger Institute, Wellcome Genome Campus, Cambridge CB10 1SA, UK; 8 Department of Applied Mathematics and Theoretical Physics, Centre for Mathematical Sciences, University of Cambridge, Cambridge CB3 0WA, UK; 9 Wellcome Trust Centre for Stem Cell Research, University of Cambridge, Cambridge CB2 0AW, UK

**Keywords:** Limb regeneration, *Xenopus*, Apical-ectodermal-ridge, scRNA-Seq, *Ex vivo* limbs

## Abstract

Absence of a specialized wound epidermis is hypothesized to block limb regeneration in higher vertebrates. However, the factors preventing its formation in regeneration-incompetent animals are poorly understood. To characterize the endogenous molecular and cellular regulators of specialized wound epidermis formation in *Xenopus laevis* tadpoles, and the loss of their regeneration competency during development, we used single-cell transcriptomics and *ex vivo* regenerating limb cultures. Transcriptomic analysis revealed that the specialized wound epidermis is not a novel cell state, but a re-deployment of the apical-ectodermal-ridge (AER) programme underlying limb development. Enrichment of secreted inhibitory factors, including *Noggin*, a morphogen expressed in developing cartilage/bone progenitor cells, are identified as key inhibitors of AER cell formation in regeneration-incompetent tadpoles. These factors can be overridden by *Fgf10*, which operates upstream of *Noggin* and blocks chondrogenesis. These results indicate that manipulation of the extracellular environment and/or chondrogenesis may provide a strategy to restore regeneration potential in higher vertebrates.

## Abstract (precis)

**Summary:** Secreted inhibitors associated with chondrogenic progression inhibit AER cell formation and restrict limb regeneration potential.

## INTRODUCTION

Amphibian limb regeneration relies on a specialized wound epidermis (also known as the apical-epithelial-cap, AEC) that forms on the amputation plane and has been characterized primarily as a tissue in regenerating salamander limbs (Campbell et al., 2011; Campbell and Crews, 2008; Knapp et al., 2013; Monaghan et al., 2012; Pearl et al., 2008; Tsai et al., 2020, 2019). It has been hypothesized that the absence or immature state of this tissue limits the regeneration potential of higher vertebrates, including mammals (Tassava and Olsen, 1982). The AEC has been suggested to impact underlying tissues by: degrading extracellular matrix (Kato et al., 2003; Miyazaki et al., 1996; Yang et al., 1999); secreting growth factors to promote proliferation (Han et al., 2001; Thornton, 1960; Thornton and Thornton, 1965; Tsai et al., 2020); enabling the self-renewal of underlying progenitor and dedifferentiated cells, leading to the formation of a proliferative structure called the blastema (Mescher, 1976; Tassava and Loyd, 1977; Tassava and Mescher, 1975); and providing directionality cues for growth (Ghosh et al., 2008; Thornton, 1960; Thornton and Thornton, 1965). Some marker genes associated with AEC (e.g. *Fgf8* and *Fn1*) were seen only in the basal layers of AEC tissue, suggesting there is cellular heterogeneity within the AEC (Christensen and Tassava, 2000; Tsai et al., 2020; Yokoyama et al., 2000). However, it remains largely unclear which cell types within AEC tissue are crucial for regeneration, which transcriptional and functional properties are associated with a mature AEC and regeneration, and why the AEC cannot form or maturate in some instances and/or species.

Owing to their requirement for proximal-distal outgrowth as well as the similarity in *Fgf8* expression patterns, the AEC in regenerating limbs was suggested to be analogous to the apical-ectodermal-ridge (AER), a tissue that has been well-studied during mouse and chicken limb development (Beck et al., 2009). However, current results suggest that limb regeneration-competent salamanders lack a developmental AER (Purushothaman et al., 2019). Moreover, recent findings (including single-cell transcriptomic data) have provided conflicting results on epidermal *Fgf8* expression during axolotl limb regeneration (Gerber et al., 2018; Han et al., 2001; Leigh et al., 2018; Li et al., 2020; Nacu et al., 2016; Qin et al., 2020; Rodgers et al., 2020; Vincent et al., 2020). Therefore, it is unclear whether cells within AEC tissue use a novel transcriptional programme for regeneration, or whether they re-deploy a transcriptional programme associated with developmental AER.

*Xenopus laevis* is the only commonly used model organism that develops its limbs in a similar manner to amniotes, has a detectable AER and shows limb regeneration ability (Purushothaman et al., 2019). Moreover, tadpoles lose their limb regeneration ability progressively during development, coinciding with their inability to form a specialized wound epidermis, although the mechanisms of regeneration incompetence and their connection to the specialized wound epidermis remain incompletely understood (Christen and Slack, 1997; Dent, 1962). At the developmental stages prior to the formation of digits, amputations lead to a complete regeneration of the limb [Nieuwkoop and Faber stage (NF) ∼52-54 (Nieuwkoop and Faber, 1994), regeneration competent]. As autopod development proceeds, amputations result in partial regeneration, characterized by missing digits (NF ∼55-57, regeneration restricted). Towards metamorphosis, amputations either cause the growth of an unpatterned spike-like cartilaginous structure without joints and muscles, or a simple wound-healing response (NF ∼58 and beyond, regeneration incompetent) (Beck et al., 2009; Dent, 1962). In addition to being stage dependent, *Xenopus* limb regeneration competence depends on amputation position, and is reduced when amputations are performed at more proximal regions of the limb, where there are more mature chondrogenic and osteogenic cells (Nye and Cameron, 2005; Wolfe et al., 2000). Likewise, amputation through bone results in reduced regeneration compared with amputations at the joints (Nye and Cameron, 2005; Wolfe et al., 2000). Nonetheless, the association between this stage and position dependence, and regeneration competency remains unclear.

Regeneration incompetency was suggested to result from changes in mesodermal tissue, and may involve defects in patterning of the blastema (Sessions and Bryant, 1988; Yokoyama et al., 2001). In particular, the lack of activating signals (e.g. *Fgf10*) was proposed to prevent the formation of a specialized wound epidermis (Yokoyama et al., 2001). However, these studies were performed at the tissue level, and it remains unclear which individual cell types within the tissue are responsible for regeneration incompetency, whether intrinsic properties of mesodermal cell types fail to activate upon injury, and whether inhibitory secreted factors, rather than a lack of activating factors, plays a role in determining regeneration-outcome. Additionally, exogenous perturbations to major signalling pathways [e.g. BMP (Beck et al., 2006; Pearl et al., 2008), FGF (D'Jamoos et al., 1998) and WNT (Yokoyama et al., 2011)] have been shown to inhibit regeneration. However, it is largely unknown how these pathways endogenously influence cell types and cellular behaviours during regeneration, or how these different pathways operate in the context of cell-cell interactions that mediate regeneration. Overall, although there are numerous tissues and genes implicated in limb regeneration competency, there is currently no unifying cellular model accounting for these disparate observations.

Here, by using single-cell RNA-sequencing (scRNA-seq), we define the cellular framework of specialized wound epidermis formation during regeneration and its failure to form at later developmental stages. Then, by using scRNA-seq and *ex vivo* limb cultures, we reveal the crucial role of secreted inhibitory factors in determining regeneration competency, and test this phenotype by using regeneration-associated genes. Together, these findings implicate a cellular mechanism in which factors secreted during bone/cartilage formation inhibit the formation of specialized wound epidermis at later developmental stages, compromising regeneration competency.

## RESULTS

### Single-cell RNA-seq analysis reveals cell type heterogeneity during development and following amputation of the limb

**Fig. 1.**
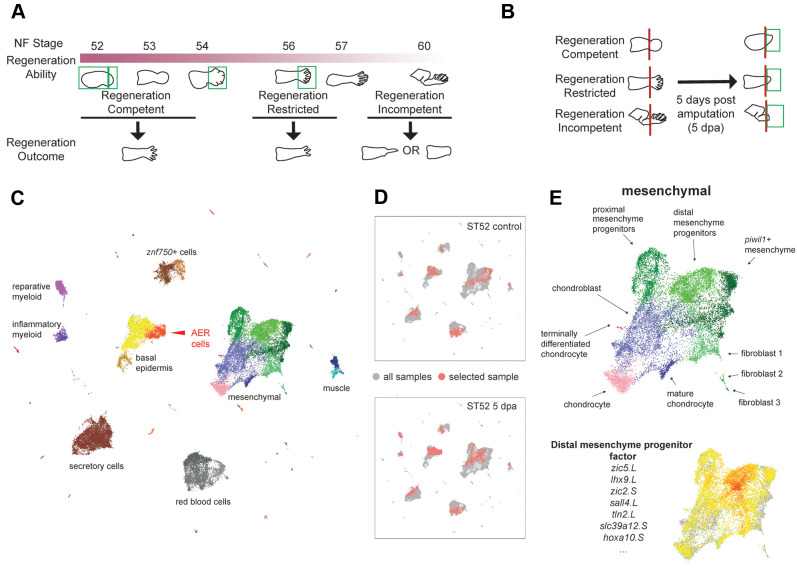
**Single-cell transcriptomics reveals cellular heterogeneity in developing and amputated *Xenopus* limbs at different stages of regeneration competence.** (A) Schematic describing *Xenopus* limb regeneration at different NF stages. NF stage ∼52-54 tadpoles are regeneration competent and amputations result in regeneration of a full limb. Regeneration ability begins to decline at NF stage ∼54. Tadpoles are regeneration restricted at NF ∼stage 56, where two or three digits can be regenerated. Beyond NF stage ∼58, tadpoles are regeneration incompetent and amputations result in simple wound healing or unpatterned spike formation. Green boxes indicate the location of samples collected for scRNA-seq, taken at stages prior to, at the onset of and after the loss of regeneration ability. (B) Schematic describing 5 days post-amputation (dpa) samples for regeneration-competent, -restricted and -incompetent tadpoles. Green boxes indicate the location of samples collected for scRNA-seq. (C) An atlas of cell types in intact and amputated limbs. Samples from each condition are processed separately for sequencing, and are then pooled together for UMAP visualization and clustering. Each dot corresponds to a single cell, colours indicate cluster identity, text labels important tissue/cell types. See Fig. S3 for full annotation. (D) Comparisons can be made between conditions to highlight transcriptional changes associated with regeneration; here, NF stage 52 amputated limbs (bottom) are compared with their contralateral control samples (top). Red dots indicate cells in the selected sample; grey dots indicate cells in all samples. (E) Diversity of mesenchymal cell types detected in our dataset (top), together with putative gene expression programmes identified using unbiased factor analysis (bottom).

To compare differences in AER and AEC, as well as to detail the cellular landscape of regeneration, we used single-cell transcriptomics. To characterize developmental AER and cellular changes associated with regeneration ability, we first sequenced developing intact hindlimbs at specific morphologically defined stages: NF stage ∼52 (limb bud stages), NF stage ∼54 (autopod forming) and NF stage ∼56 (autopod formed) (Fig. 1A). Then, to evaluate regeneration-associated AEC and the cellular responses to amputations, we profiled cells from amputated limbs and their contralateral controls. Specifically, we amputated hindlimbs from presumptive knee/ankle levels for regeneration-competent tadpoles (NF stage ∼52-53) and ankle level for regeneration-restricted (NF stage ∼55-56) and regeneration-incompetent (NF stage ∼58-60) tadpoles, and sequenced cells from newly-generated tissues at 5 days post-amputation (dpa) (Fig. 1B) when the specialized wound epidermis and blastema are seen morphologically (Beck et al., 2009). Contralateral developing limb buds or autopods were sequenced as controls. We did not include a contralateral control at the regeneration-incompetent stage as our dissociation protocol was unable to dissociate bone cells without compromising other tissues.

Next, we pooled the single-cell RNA-sequencing data derived from at least two replicates for each condition (Fig. S1), and corrected our atlas for cell cycle effects (Fig. S2), yielding a total of 42,348 cells (Materials and Methods; Fig. 1C,D; Figs S3 and S4; Table S1). After clustering of cells based upon their gene expression profiles, examination of multiple marker genes (Fig. S5) revealed at least 60 distinct clusters representative of putative cell types (Fig. 1C and Fig. S3), including known populations (e.g. AER cells) and potentially new and uncharacterized cell states (e.g. a *Piwil1*^+^ population in the mesenchyme) (Fig. 1E). From the cell atlas, we were able to detect cell cycle differences between cell types, e.g. distal mesenchyme progenitors were more biased towards G2/M phases compared with proximal mesenchyme progenitors (Fig. S2), as reported in mouse (Boehm et al., 2010). The *Xenopus* limb cell atlas is accessible using an interactive platform (https://marionilab.cruk.cam.ac.uk/XenopusLimbRegeneration/).

### Quantitative features of AER cell formation are associated with regeneration outcome

**Fig. 2.**
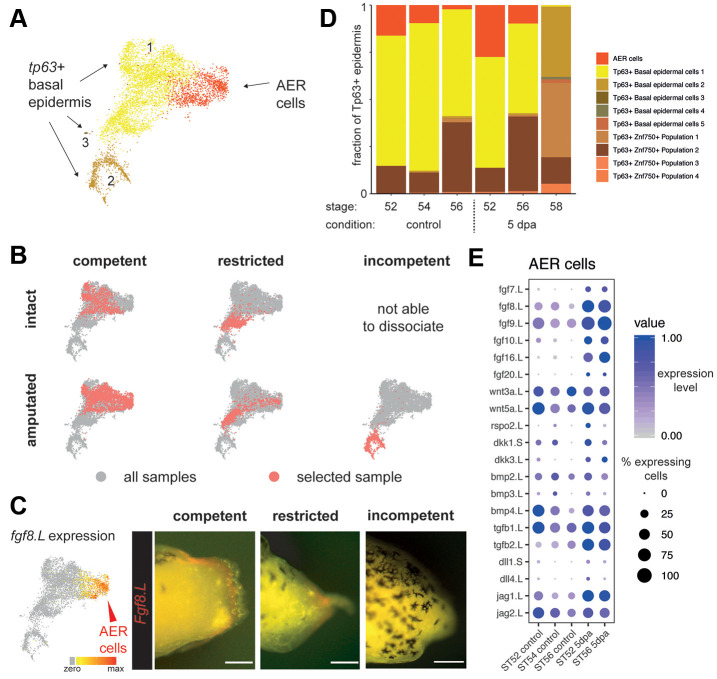
**Formation of a signalling centre comprising apical-ectodermal-ridge (AER) cells is associated with the successful regeneration.** (A) Multiple basal epidermal cell states are detected, including AER cells, in the pooled dataset. (B) UMAP visualization of basal epidermis reveals that re-establishment of AER cells is associated with successful regeneration. Red dots indicate cells in the selected sample; grey dots indicate cells in all samples. (C) Left: UMAP visualization of pooled data for AER cells expressing *Fgf8.L*. Right: stereomicroscope images of the 5 dpa amputation plane of regeneration-competent, -restricted and -incompetent tadpoles. *Fgf8.L-*expressing AER cells (red) are formed in regeneration-competent and -restricted tadpoles, but not in regeneration-incompetent tadpoles. Scale bars: 250 μm. (D) The abundance of basal epidermal cell types across conditions reveals a correlation between AER abundance and regeneration outcome. AER cells are present in intact regeneration-competent samples, and are enriched after amputation. A similar pattern is seen in regeneration-restricted samples, although abundances of AER cells are reduced. Very few AER cells are detected in regeneration-incompetent tadpoles. (E) Dot plot showing expression of selected ligands for AER cells during development and at 5 dpa in regeneration-competent and -restricted samples. Dot colour indicates mean expression; dot size represents the percentage of cells with non-zero expression.

We then focused on the specialized wound epidermis, or AEC, that was suggested to be analogous to the AER. Although both populations are characterized by *Fgf8* expression (Beck et al., 2009), the extent of the similarity between these cells has not been previously tested beyond assessing the similarity of expression of a small number of markers. Using our single-cell atlas, we compared the transcriptional profiles of cells that belonged to the AER (defined as *Fgf8*-expressing epidermal cells during limb development) and the AEC (*Fgf8*-expressing epidermal cells in 5 dpa samples). Although we did see some quantitative expression differences between cells related to AEC and AER tissues (Figs S5, S6A, Table S2), they expressed many genes in common and showed a high degree of transcriptional similarity (Fig. 2A,B, Fig. S5). Consistent with this, cells related to these tissues were aggregated within a single *Fgf8*^+^ epidermal cluster (Fig. 2B,C). Additionally, both during development and 5 days post-amputation, *Fgf8^+^* epidermal cells were mostly detected as a monolayer of polarized cuboidal basal cells (Fig. S7), although multilayers were seen to form in some instances (Fig. S8). This suggests that AEC and AER tissues are not homogenous in their cellular composition, and that it is only the basal cells that express the key *Fgf8*^+^ transcriptional programme. Overall, based on their transcriptomic signature, tissue localization and cellular morphology, the *Fgf8^+^* cells that compose the AEC and AER tissues are very similar. We find that the AEC tissue does not require a novel cell state, but rather a re-deployment of the transcriptional programme associated with developmental AER, albeit with a higher signalling centre potential (Fig. 2E, Fig. S6C,D). Owing to their high degree of similarity and common expression of developmental AER genes, we named all cells from the identified *Fgf8^+^* epidermal cluster as AER cells, and referred to specific samples to distinguish between cells from the regeneration-associated AEC and the developmental AER.

To test the similarities of cell types composing the specialized wound epidermis in different regeneration conditions, we compared transcriptomes of cells corresponding to limb and tail specialized wound epidermis. We found that AER cells (limb-specialized wound epidermal cells) and cells that define the specialized wound epidermis during *Xenopus* tail regeneration (regeneration-organizing cells, ROCs; Aztekin et al., 2019) showed similar, but non-identical, gene expression profiles (Fig. S9), emphasizing that the cellular framework of the specialized wound epidermis is context dependent and appendage regeneration scenarios can use different cell types.

Limb amputation is known to result in the formation of a *Fgf8*-expressing AEC at the amputation plane in regeneration-competent tadpoles, but not in regeneration-incompetent tadpoles (Christen and Slack, 1997), whereas AEC formation has not been characterized previously for regeneration-restricted tadpoles. Using our atlas, we found that, at 5 dpa, tadpole epidermis contained abundant AER cells in regeneration-competent tadpoles and a limited number of AER cells in regeneration-restricted tadpoles, whereas AER cells were largely absent from regeneration-incompetent tadpoles (Fig. 2B-D). In parallel, AER cell-associated ligand expression was lower or absent in regeneration-incompetent *tp63*^+^ epidermal cells (Fig. S6E). In our dataset, we found that different populations express ligands from different major signalling pathways (FGF, BMP, WNT, DELTA and TGFβ) (Fig. S6C). However, only AER cells can express multiple ligands from these gene families concurrently and at a very high level, making them a highly potent signalling centre (Fig. 2E, Fig. S6). Although *Fgf8* was always expressed in AER cells, the relative expression of *Fgf8* and other ligands varied among conditions (Fig. 2E, Fig. S6, Table S2), emphasizing that the detection of *Fgf8* alone does not discriminate the signalling centre potency of AER cells. Indeed, in addition to the changes in AER cell abundance, we also detected differentially expressed genes between AER cells from regeneration-competent and -restricted tadpoles (Fig. S6B). These differences suggest that AER cells in regeneration-competent 5 dpa samples may be more ‘mature’ compared with regeneration-restricted cells, although further work on the functional role of these genes (e.g. *Sesn1.L*, *Cpn1.S*, *Ddx21.L*) is required. Overall, although the signalling centre potency of AER cells appeared variable, the redeployment of this developmental cell type with a high signalling centre potential had a strong correlation with regeneration outcome.

### The presence of AER cells is associated with injury-induced mesenchymal plasticity

It has been suggested that the AEC enables the self-renewal activity of dedifferentiated cells, leading to blastema formation (Tassava and Mescher, 1975; Tassava and Loyd, 1977). To identify signatures of dedifferentiation in our atlas, we first examined the expression of genes related to dedifferentiation and blastema formation (Gerber et al., 2018; Haas and Whited, 2017; Leigh et al., 2018) (e.g. *Sall4* and *Kazald1*). We found that these genes were either already expressed before amputation or upregulated upon amputation in a subset of fibroblasts (Fig. S10A,B) that were located near the skin and perichondrium (Fig. S11). Likewise, we found that a small fraction of these fibroblasts expressed muscle-related genes (e.g. *Pax3*) before and after amputation (Fig. S10B). Moreover, independently of regeneration outcome, amputation resulted in these fibroblast cells expressing genes related to distal mesenchyme progenitors (e.g. *Grem1*, *Shh*, *Msx1* and *Fgf10*) and chondrogenesis (e.g. *Col8a2* and *Sox9*) (Fig. S10A). Finally, amputation not only increased the expression of known marker genes, but also led to the upregulation of an entire putative distal mesenchyme progenitor gene set (Fig. S10C), with the magnitude of this upregulation being lower in samples that had fewer AER cells. Together, we concluded that, upon amputation, a subset of fibroblasts manifest injury-induced mesenchymal plasticity – at least at the transcriptional level – and its extent correlates with AER cell abundance.

### AER cell formation requires activation of multiple signalling pathways

**Fig. 3.**
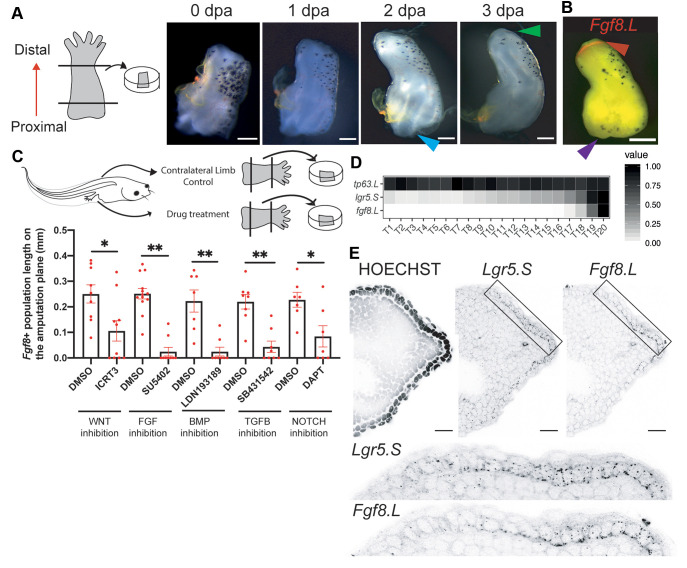
***Ex vivo* regenerating limbs demonstrate that AER cell formation requires activation of multiple pathways and can form from basal epidermal cells.** (A) Left: schematic for *ex vivo* regeneration limb culture. Right: time-lapse images of a regeneration-competent explant. The explant grows a cone shape at its distal site reminiscent of *in vivo* regeneration (green arrowhead), while the proximal site shows chondrogenesis (blue arrowhead). Scale bars: 200 μm. (B) An example image of a regeneration-competent explant at 3 days post-culture. The distal site of explants is *Fgf8.L* positive (red arrowhead); the proximal site is *Fgf8.L* negative (purple arrowhead). Red, *Fgf8.L* mRNA. Scale bar: 200 μm. (C) Drug screen to test regulators of AER cell formation. Top: schematics describing the screen. One limb of a tadpole was used for perturbation and the contralateral limb from the same tadpole was used as a control. Samples were treated with the indicated drugs for 3 days post-culture, and then stained for *Fgf8.L* mRNA. Bottom: the extent of *Fgf8.L* expression along the amputation plane was measured. Sample sizes: ICRT3 total, *n*≥9 from three biological replicates; SU5402 total, *n*≥9 from two biological replicates; LDN193189 total, *n*=8 from three biological replicates; SB431542 total, *n*=8 from two biological replicates; DAPT total, *n*=7 from three biological replicates. *P<0.05, **P<0.001. Data are mean±s.e.m. (D) Factor analysis identifies a putative gene expression trajectory from basal epidermal cells to AER cells, predicting sequential activation of *Lgr5.S* followed by *Fgf8.L*. (E) A proximal-to-distal gradient of *Lgr5.*S and *Fgf8.L* is observed *in vivo*, with *Fgf8.L* being restricted to the most distal regions of the midline epidermis. Black dots represent HCR mRNA signal. Scale bars: 20 μm.

To investigate the molecular mechanisms that mediate AER cell formation upon amputation, we developed an *ex vivo* regenerating limb culture protocol, inspired by previous work (Cannata et al., 1992) (Fig. 3A). By culturing amputated stylopod, or zeugopod and stylopod from regeneration-competent or regeneration-restricted tadpoles, respectively, we observed *Fgf8* cell formation at the distal part of explants within 3 dpa (Fig. 3B). Regeneration-competent explants also exhibited cone-shaped growth as cells accumulated uniformly underneath *Fgf8* cells, mimicking *in vivo* regeneration (Fig. 3A,B, Fig. S12A,B). Interestingly, the proximal region of explants was also covered with epidermis (Figs S12A, S13A), but neither *Fgf8*-expressing cells nor a uniform cell accumulation underneath the epidermis was observed (Fig. 3A,B, Figs S12B, S13A). Moreover, the proximal part of the explant exhibited active chondrogenesis, manifesting in an outwards growth of cartilaginous tissue (Fig. 3A and Fig. S12C). This phenotype was particularly pronounced when explants were harvested from developmental stages in which proximal tissues were advanced in chondrogenesis (onset of NF stage 53-54) (Fig. 3A and Fig. S12D), and could be further enhanced by addition of BMP4, a known chondrogenesis inducer (Fig. S12E). Hence, the proximal and distal sites of limb explants exhibit different behaviours: the distal sites recapitulate localized AER cell formation, as seen *in vivo*, whereas the proximal site is characterized by active chondrogenesis without AER cell formation.

In addition to changes associated with regeneration, explants could be used to determine signalling requirements for specialized wound epidermis formation. Inhibition of FGF, BMP and WNT pathways via small molecule inhibitors blocked AER cell formation in explants (Fig. 3C), reinforcing the conclusion that the *in vivo* AEC effects reported in former studies are mediated through a direct effect on the limb rather than a systemic effect (Beck et al., 2006; D'Jamoos et al., 1998; Yokoyama et al., 2011). Moreover, by using the culture assay, we found that active TGFβ and NOTCH signalling are also required for *Xenopus* AER cell formation (Fig. 3C). Overall, we concluded that AER cell formation requires the activity of multiple major signalling pathways, although further work is required to determine what roles these pathways play and whether they directly or indirectly regulate AER cell formation.

### AER cells can form without cell division

Next, we asked how AER cells form on the amputation plane. It has been suggested that salamander AEC tissue forms by migration of epidermal cells to the amputation plane, and may not require cell proliferation (Campbell and Crews, 2008; Hay and Fischman, 1961). Moreover, the mouse AER was previously suggested to be a largely mitotically inactive tissue (Storer et al., 2013). However, it is not known whether similar mechanisms apply to AER cells within the specialised wound epidermis, and also to what extent they are seen in *Xenopus.* Therefore, we first traced skin tissue located on the edge of explants, and found that they contributed to the covering of both the distal and proximal sites (Fig. S13B). As the amputation planes are covered by skin tissue from the surrounding area, we reasoned that AER cells are likely to have originated from skin cells. As amputation eliminates the majority, if not all, of AER cells in the limb, we hypothesized that AER cells are derived from remaining skin stem cells. If AER cells are induced through proliferation and differentiation following amputation, all AER cells should be the product of cell division. To test this hypothesis, we assayed the level of EdU incorporation (labelling newly synthesized DNA, hence divided cells) in newly formed AER cells, using *Fgf8* positivity to specifically identify AER cells within the AEC tissue. We found that only ∼40% of AER cells (distal epidermal *Fgf8*^+^) were EdU positive at 3 dpa (Fig. S13C), suggesting that most AER cells are induced independently of cell division following amputation. These results parallel our transcriptomics-based cell-cycle assessment in which AER cells display low levels of proliferation (Fig. S2D). Using the transcriptomics data, we identified a stepwise activation of *Lgr5.S* (a WNT target gene) followed by *Fgf8.L* expression as a possible gene-expression trajectory that could allow basal epidermal cells to convert directly to AER cells without cell division (Fig. 3D). Consistent with such a process, when visualized *in vivo*, we found that *Fgf8*^+^*/Lgr5*^+^ AER cells were flanked by *Lgr5*^+^ cells in the basal epidermis on the amputation plane or in the developing limb (Fig. 3E and Fig. S7A,B). Overall, these results support the hypothesis that basal epidermal cells can acquire AER cell identity without cell division, although understanding the functional relevance of cell division on AER cell fate requires further work.

### Loss of regeneration potential is associated with enrichment in inhibitory secreted factors to AER cell formation

We then asked why fewer or no AER cells form on the amputation plane of regeneration-restricted or -incompetent tadpoles, respectively. Previous studies have proposed that lack of activating signals in the mesodermal tissue, specifically *Fgf10*, causes regeneration incompetency (Yokoyama et al., 2001). However, these results cannot explain why regeneration is impaired when amputations are conducted through bone or at more-proximal limb regions, or why the proximal site of limb explants cannot form AER cells. Thus, we assessed whether *Fgf10* can induce *Fgf8* expression across the whole epidermis or whether there are additional requirements for distal epidermal *Fgf8* expression, and hence AER cell formation.

When we examined the spatial correlation between *Fgf10.L*-expressing mesenchymal cells and *Fgf8.L*-expressing epithelial cells in regeneration-competent tadpoles, we saw regions in which *Fgf10.L* but not *Fgf8.L* was present (Fig. S14A). Second, when adding FGF10 to regeneration-competent explants, we observed a slight, but not statistically significant, increase in AER cell formation on the amputation plane (Fig. S14B); however, this signal was confined to the distal epidermis and did not include a substantial signal at the proximal site of explants (Fig. S14C), where chondrogenic populations are more abundant. This suggested that FGF10 alone cannot induce AER cell formation across the entire epidermis, and that the presence/absence of further activating/inhibitory signals are involved in AER cell formation.

**Fig. 4.**
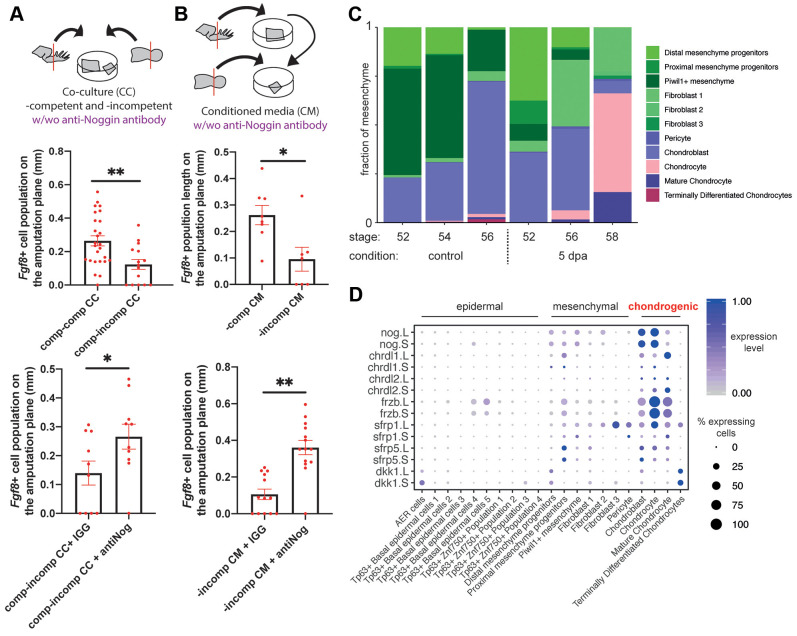
**Inhibitory factors, such as Noggin, are secreted from chondrogenic populations at regeneration-incompetent stages and block AER cell formation.** (A) Top: schematic describing co-culture experiments. Middle: co-culturing regeneration-competent and -incompetent explants decreases the extent of *Fgf8.*L expression at the amputation plane at 3 dpa. Bottom: this effect can be rescued by adding anti-NOGGIN antibody. Regeneration-competent and -competent co-culture total, *n*=26 from four biological replicates; regeneration-competent and -incompetent co-culture total, 15 from four biological replicates; competent and incompetent co-culture, and anti-IGG antibody total, *n*=10 from three biological replicates; competent and incompetent co-culture, and anti-NOGGIN antibody total, *n*=10 from three biological replicates. *P<0.05 and **P<0.001. Data are mean±s.e.m. (B) Top: schematic describing conditioned media experiments to test the effect of secreted factors in regeneration-incompetent tadpole limbs. Middle: supplying conditioned media (CM) from regeneration-incompetent tadpoles to regeneration-competent explants decreases the extent of *Fgf8.*L expression at the amputation plane at 3 dpa. Bottom: this effect can be rescued by adding anti-NOGGIN antibody. Regeneration-competent CM to -competent explants total, *n*=8 from three biological replicates; regeneration-incompetent CM to -competent explants total, *n*=7 from three biological replicates; regeneration-incompetent CM to -competent explants, and anti-IGG antibody total, *n*=10 from three biological replicates; regeneration-incompetent CM and anti-NOGGIN antibody to regeneration-competent explants total, *n*=10 from three biological replicates. *P<0.05 and **P<0.001. Data are mean±s.e.m. (C) Abundance of mesenchymal populations across conditions reveals an enrichment of chondrogenic populations at regeneration-restricted and -incompetent stages, in both intact and amputated limbs. (D) Multiple BMP/WNT antagonists are expressed specifically in chondrogenic populations. This dotplot is generated using the pooled dataset, with late-stage tadpoles having high levels of chondrogenic and fibroblast populations, but not immature mesenchymal cell types, as shown in C.

To test whether there are inhibitory factors secreted from regeneration-incompetent tadpole limbs that block AER cell formation, we took advantage of our *ex vivo* cultures. First, we co-cultured *ex vivo* limbs from regeneration-competent and -incompetent tadpoles. Strikingly, when such cultures were stained against *Fgf8* at 3 dpa, we observed that regeneration-competent tadpole limbs failed to form AER cells (Fig. 4A). Second, we collected media from regeneration-incompetent tadpole explants and cultured freshly amputated regeneration-competent explants with this conditioned media. Consistent with the co-culture experiment, the conditioned media from regeneration-incompetent tadpoles blocked AER cell formation in regeneration-competent explants (Fig. 4B). By contrast, neither co-culturing with regeneration-competent explants nor preparing conditioned media from regeneration-competent explants affected AER cell formation in regeneration-competent explants (Fig. 4A,B). Additionally, conditioned media from regeneration-competent explants was unable to induce AER cell formation in regeneration-incompetent explants (Fig. S15A,B), emphasizing that it is the enrichment of inhibitory secreted factors that is the dominant process interfering with AER cell formation, rather than a depletion in regeneration-promoting factors. Altogether, these results suggest that secreted inhibitory factors block AER cell formation in regeneration-incompetent tadpoles, presumably compromising their regeneration potential.

**Fig. 5.**
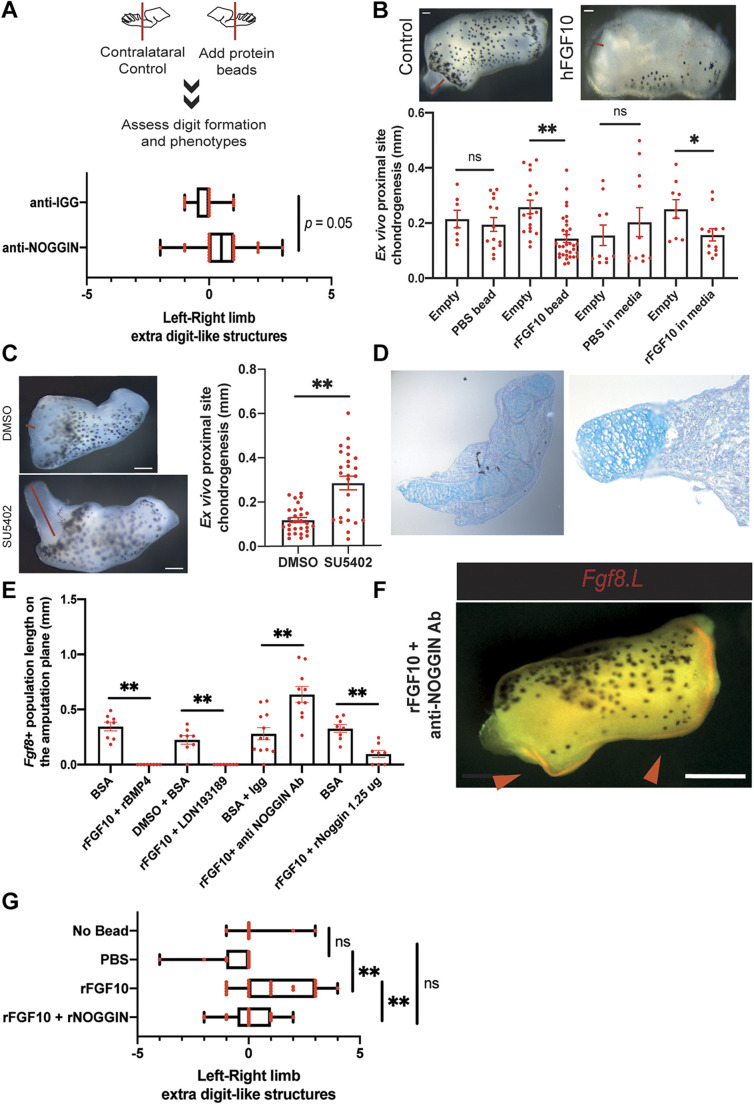
**FGF10 impacts chondrogenesis and operates upstream of NOGGIN.** (A) Anti-NOGGIN antibody application to distal amputations improves regeneration in regeneration-restricted and -incompetent tadpoles. Regeneration-restricted and -incompetent tadpole right and left hindlimbs were amputated, and beads containing anti-IGG antibody or anti-NOGGIN antibody were placed on the right hindlimbs. Formed digits and digit-like structures were quantified in the right and left hindlimbs, and the difference calculated. Anti-IGG antibody total, *n*=17 from three biological replicates; anti-NOGGIN antibody total, *n*=28 from four biological replicates. (B) The effect of FGF10 on chondrogenesis is assessed by measuring the chondrogenic outgrowth at the proximal sites of regeneration-restricted explants at 3 dpa. Red lines show measured proximal chondrogenesis. Implanting 0.1% BSA/PBS beads at the proximal site or supplying 0.1% BSA/PBS to the media had no significant effect on chondrogenesis, while implanting Fgf10 beads at the proximal site or supplying FGF10 in media reduced chondrogenesis. Contralateral limbs were used as controls and are labelled as empty. From left to right, empty and PBS beads total, *n*≥7 from at least two biological replicates; empty and FGF10 bead total, *n*≥14 from at least four biological replicates; empty and 0.1% BSA/PBS in media total, *n*=10 from three biological replicates; empty and FGF10 in media, *n*≥14 from at least three biological replicates. ns, not significant; *P<0.05 and **P<0.001. Data are mean±s.e.m. Scale bar: 50 µm. (C) Left: example images of SU5402-treated explants showing extensive chondrogenesis at the proximal site. Red lines show measured proximal chondrogenesis. Right: blocking FGFR via the small molecule inhibitor SU5402 extends chondrogenesis in 3 days for regeneration-competent and -restricted explants. Contralateral limbs were used as controls and treated with DMSO. DMSO total, *n*=29 from seven biological replicates; SU5402 total, *n*=25 from seen biological replicates. **P<0.001. Data are mean±s.e.m. Scale bar: 200 µm. (D) Representative sectioned histology images for 3 dpa explants treated with SU5402. The outgrowing structures are Alcian Blue rich, which is indicative of chondrogenic cells. (E) Regeneration-competent explants were treated with a combination of FGF10 and recombinant BMP4, or with recombinant NOGGIN, with LDN193189 or with anti-NOGGIN antibody. 0.1% BSA/PBS and anti-IGG antibody were used as controls. From left to right: BSA total, *n*=8 from two biological replicates; recombinant FGF10 and recombinant BMP4 total, *n*=8 from two biological replicates; DMSO and BSA total, *n*=8 from two biological replicates; FGF10 and LDN total, *n*=8 from two biological replicates; BSA and anti-IGG antibody total, *n*=12 from three biological replicates; FGF10 and anti-NOGGIN antibody total, *n*=10 from three biological replicates; BSA total, *n*=8 from two biological replicates; recombinant FGF10 and recombinant NOGGIN total, *n*=8 from two biological replicates. **P<0.001. Data are mean±s.e.m. (F) Representative whole-mount stereomicroscope image of rFGF10 and anti-NOGGIN antibody treated explants can show a substantial *Fgf8.L* expression at different sites of the explant (*n*=5/9 from two biological replicates, compared with *n*=0/121 in controls, *P*<0.0001). Scale bar: 200 μm. (G) Recombinant FGF10 application to distal amputations restore regeneration in -regeneration-restricted and -incompetent tadpoles. Regeneration-restricted and -incompetent tadpole right and left hindlimbs were amputated, and beads containing 0.1% BSA/PBS or recombinant FGF10 or recombinant FGF10 and NOGGIN were placed on the right hindlimbs. Formed digits and digit-like structures were quantified in the right and left hindlimbs and the difference calculated. Box and whisker plots show minimum to maximum for all data points. Red dots represent individual data points. Where applicable, boxes show interquartile range (25-75%). Mean for No bead: 0.15; PBS: −0.5; rFGF10: 1.04; rFGF10 + rNOGGIN: 0. Empty total, *n*=19 from two biological replicates; 0.1%/PBS bead total, *n*=17 from five biological replicates; recombinant FGF10 bead total, *n*=25 from five biological replicates; recombinant FGF10 and NOGGIN bead total, *n*=25 from four biological replicates. ns, not significant; **P<0.001.

To identify the factors responsible for this inhibitory effect, we surveyed our single-cell atlas for the expression of secreted proteins involved in signalling pathways required for AER cell formation. We found that that the loss of regeneration potential is associated with an increased proportion of chondrogenic lineage cells in the mesenchyme [Fig. 4C, aligning with previous tissue-level observations (Dent, 1962)] and that these cells express multiple inhibitory ligands for BMP and WNT pathways (Fig. 4D). As chondrogenic populations specifically express high levels of *Noggin* (Fig. 4D), a known antagonist of BMP signalling, we hypothesized that AER cell formation is antagonized by an excess of secreted *Noggin* in regeneration-incompetent tadpoles. Indeed, consistent with previous observations (Pearl et al., 2008), addition of NOGGIN to regeneration-competent *ex vivo* limbs blocked AER cell formation (Fig. S15C). To test whether endogenous NOGGIN does indeed act as one of the inhibitory secreted factors produced following amputation in regeneration-incompetent tadpoles, we blocked NOGGIN in our co-culture and conditioned media experiments using anti-NOGGIN antibodies (Fig. 4A,B). Strikingly, blocking secreted NOGGIN by antibody addition cancelled the inhibitory activity on AER cell formation in both co-culture and conditioned media experiments (Fig. 4A,B). Based on these observations, we then explored whether anti-NOGGIN application would improve the *in vivo* amputation response. Indeed, when beads loaded with anti-NOGGIN antibodies were implanted on the amputation plane of regeneration-restricted/incompetent tadpoles, we saw a mild improvement in the regenerative response (Fig. 5A), highlighting that secreted inhibitors are influencing the regeneration-outcome *in vivo*.

As these experiments point towards the chondrogenic lineage as the source of inhibitory secreted factors, we then asked whether limiting chondrogenesis can promote AER cell formation. To this end, we generated tip explants by culturing distal limb buds (NF stage ∼52) or early formed autopods (NF stage ∼54) without their proximal segment, where the most advanced chondrogenesis takes place. Indeed, these tip explants showed ectopic *Fgf8* expression at different sites of the epidermis, further suggesting a localized and/or long-range inhibitory effect of secreted factors from mature chondrogenic cells (Fig. S15D). Moreover, the inability of the proximal explant epidermis to form AER might be explained, at least in part, by the abundance of chondrogenic cells at the proximal site (Fig. 3A,B). Overall, these results indicate that the loss of AER cell formation ability is associated with an enrichment in inhibitory secreted factors, including NOGGIN, that are secreted primarily from the chondrogenic lineage.

As NOGGIN is known to neutralize secreted BMPs, we then focused on assessing the effect of the BMP pathway on AER cell formation. Previously, it was demonstrated that, not only do mouse and chicken AER require active BMP signalling, but also excess BMP activation abolishes AER (Pajni-Underwood et al., 2007; Pizette et al., 2001; Pizette and Niswander, 1999; Verheyden and Sun, 2008). To test whether manipulation of BMP signalling can also impact *Xenopus* AER cell formation in regeneration-competent tadpoles, we perturbed the BMP pathway. We found that the addition of BMP4 to regeneration-competent *ex vivo* cultures blocked AER cell formation (Fig. S15C), an effect similar to that reported in chick and mouse embryos (Pajni-Underwood et al., 2007; Pizette et al., 2001; Pizette and Niswander, 1999; Verheyden and Sun, 2008). The addition of NOGGIN to regeneration-competent *ex vivo* cultures blocked AER cell formation (Fig. S15C), as reported before *in vivo* (Beck et al., 2006; Jones et al., 2013). Moreover, we found that inhibiting NOGGIN could increase the formation of AER cells (Fig. S15C), suggesting endogenous BMP4 levels do not reach a level where AER cell formation is blocked. As BMP4 boosts chondrogenesis (Fig. S12E), which can, in turn, lead to *Noggin* expression, these results suggest that fine-tuning of BMP agonist and antagonists levels in the growing limb are key for AER cell formation.

### FGFR activation negatively regulates progression of chondrogenesis and the FGF pathway operates upstream of NOGGIN for AER cell formation

As regeneration competency in late stage tadpoles has bee shown previously to be restored via exogenous application of FGF10 (Yokoyama et al., 2001), we next sought to evaluate whether the effect of *Fgf10* on regeneration is, at least in part, mediated by its impact on chondrogenesis and *Noggin* expression. To test the effect of *Fgf10* on chondrogenesis, we used our *ex vivo* cultures to monitor the substantial chondrogenesis occurring at the proximal site of explants. Application of FGF10 beads to the proximal site of *ex vivo* cultures, or addition of recombinant FGF10 to their media, significantly decreased chondrogenesis at the proximal sites in regeneration-restricted explants (Fig. 5B). Conversely, blocking FGFR significantly extended chondrogenesis at the proximal site of explants (Fig. 5C-D, Fig. S16). Nonetheless, FGF10 treatment was not sufficient to induce strong Fgf8 expression at the proximal site of explants (Fig. S14C), which we hypothesize could be, at least in part, attributable to differences in the abundance of proposed antagonist cues. To test this hypothesis, we treated explants with a combination of FGF10 and anti-NOGGIN antibodies. Strikingly, this combination not only enhanced AER cell formation at the distal sites, but also induced ectopic *Fgf8.L* expression near the proximal sites of explants (Fig. 5E,F), further suggesting that the enrichment of inhibitory secreted factors from the chondrogenic lineage affects the ability to form AER cells. Finally, AER cell formation induced by FGF10 addition was cancelled by the addition of BMP inhibitors (NOGGIN or small molecule inhibitors) (Fig. 5E), suggesting that FGF10 acts upstream of the effect of NOGGIN *ex vivo*. To further test this finding *in vivo*, we asked whether the positive effect of FGF10 in regeneration-incompetent tadpoles could be abrogated by simultaneous NOGGIN addition. To investigate this, we inserted beads co-loaded with FGF10 and NOGGIN to the amputation plane of regeneration-restricted and/or -incompetent tadpoles, and found this significantly decreased the positive effect of FGF10-only beads (Fig. 5G). These results further emphasise that FGF10 operates upstream of NOGGIN, and hence that secreted inhibitors play a dominant role in determining regeneration outcome.

## DISCUSSION

Limb regeneration and its requirement for a mature specialized wound epidermis (the AEC) is a well-established phenomenon with extensive tissue and gene level investigations. Here, moving beyond tissue-level descriptions, we reveal cell types and transcriptional states that mediate *Xenopus* limb regeneration and AEC tissue by using single-cell transcriptomics and *ex vivo* regenerating limb cultures. Transcriptome and morphological assessment indicate that the transcriptional programmes and cells defining AEC and AER tissues are largely the same, differing only in the magnitude of their signalling centre potential. Hence, AEC does not seem to involve a novel transcriptional programme specific for regeneration-competent species, but rather the activation of a programme that is highly reminiscent of developmental AER, at least in *Xenopus*. Moreover, by identifying transcriptomic and morphological differences between the specialized wound epidermis of an amputated tail and limb, we demonstrated that, at the cellular level, appendage regeneration is context dependent and warrants caution for cross-paradigm comparisons. Indeed, it is likely that other regeneration paradigms may use different cell types and transcriptional programmes for their specialised wound epidermis [e.g. zebrafish fin AEC does not express *Fgf8* (Shibata et al., 2016)]. Nonetheless, amniotes, including humans, have a developmental AER (Kelley and Fallon, 1976). Therefore, our results suggest that mammals have the transcriptional programme to orchestrate limb regeneration, but fail to redeploy the AER cell transcriptional programme upon injury. These results prompted us to characterize regulators of AER cell formation.

Recent research has focused on the intrinsic properties of mesodermal tissue and its ability to induce specialized wound epidermis (via *Fgf10* expression), supported by the observation that transplantation of mesoderm tissue from regeneration-incompetent limbs prevents regeneration in competent *Xenopus* limbs (Sessions and Bryant, 1988; Yokoyama et al., 2000)*.* However, this approach is not able to discriminate whether cells are intrinsically incompetent or whether secreted factors cause this effect, as both would be transferred at the same time (as well as the numerous caveats associated with tissue transplantation). Moreover, this hypothesis does not explain why FGF10 is insufficient to induce AER cells across the entire epidermis or why regeneration outcomes are significantly correlated with the extent of ossification at the amputation plane (Dent, 1962; Nye and Cameron, 2005; Wolfe et al., 2000). Inspired by our scRNA-seq data, we sought to determine whether other secreted factors could also be contributing to regeneration incompetency. To our knowledge, there is no practical way to obtain secreted factors from regeneration-incompetent tadpoles and transfer them to regeneration-competent animals *in vivo*. Therefore, we established *ex vivo* cultures that faithfully recapitulated *in vivo* regeneration to test this critical hypothesis. We identified AER cells in the *ex vivo* limbs using spatially resolved and quantitative measurement of epidermal *Fgf8* via HCR (Choi et al., 2018). Our scRNA-seq data demonstrated that high epithelial *Fgf8* expression is a unique late-stage marker in the establishment of AER cell identity (Fig. 3D), and therefore *Fgf8* positivity in our experimental set-up corresponds with high precision to the AER cell type. By using our explant systems and conducting co-culture and conditioned media experiments, both of which would be inaccessible *in vivo*, we found that secreted inhibitory factors in regeneration-incompetent tadpoles negatively impact AER cell formation.

To further explore this observation, we surveyed our scRNA-seq data and saw that a number of putative inhibitors (e.g. *Noggin*) were enriched in chondrogenic cell types, suggesting that factors secreted from the chondrogenic lineage may prevent AER cell formation. To test this hypothesis, we perturbed two genes previously associated with regeneration: *Noggin* (Beck et al., 2006; Pearl et al., 2008) and *Fgf10* (Yokoyama et al., 2001). Previous analysis of these genes was limited to the study of exogenous perturbations and their effect on regeneration outcome, without providing a model involving their endogenous function and their interaction. For example, although NOGGIN overexpression was shown to block regeneration, we show here for the first time that secreted factors in regeneration-incompetent tadpoles block AER cell formation and that endogenous NOGGIN is one of the factors causing this effect. Similarly, although FGF10 was shown to restore regeneration competency, it was not known that FGF10 activity operates upstream of chondrogenesis and NOGGIN to influence regeneration-outcome. Altogether, in this work we have systematically assessed which cell types express *Fgf10* and *Noggin*, how they act on cell types to impact regeneration, and how they operate within our proposed cellular mechanism.

We then tested our model *in vivo* and found that removal of secreted inhibitors (e.g. NOGGIN) or blocking the source of secreted inhibitors (chondrogenic progression via FGF10 application) could indeed improve the regeneration outcome in regeneration-defective stages. Moreover, we demonstrated that NOGGIN attenuates the positive effect of FGF10 application, further highlighting the downstream role played by the secreted inhibitors. Overall, these results align with previous transplantation experiments showing that mesoderm from regeneration-incompetent limbs is inhibitory to regeneration (Sessions and Bryant, 1988; Yokoyama et al., 2001, 2000). However, in contrast to previous interpretations, we suggest that an important contributor to this phenomenon is the enrichment of chondrogenic cell abundance within the mesoderm tissue that express inhibitory secreted factors.

We further showed that, by manipulating NOGGIN and FGF10 levels, we could improve amputation outcomes in regeneration-restricted and/or -incompetent tadpoles. We saw that anti-NOGGIN beads had a mild effect compared with FGF10 beads (Fig. 5A,G), which may suggest that there are other inhibitors secreted from the chondrogenic lineage (e.g. *Chrdl1* and *Frzb*) that must also be eliminated to ensure robust regeneration. However, the mild effect of anti-NOGGIN may also be due to technical problems with the perturbation (e.g. limited duration and/or diffusivity of antibody delivery), and that a more-complete inhibition of NOGGIN function would further improve the amputation outcome.

It is well established that a salamander blastema will form only in a location distal to the amputation plane, a phenomenon termed as the rule of distal transformation (Butler, 1955; Nacu and Tanaka, 2011; Stocum, 1981). In our explants, we also detect that only distal sites started to form a blastema (Fig. 3A), aligning with the rule of distal transformation. Interestingly, by manipulating NOGGIN and FGF10, we also could observe AER cell formation at the proximal sites of explants (Fig. 5F). However, it remains unclear whether these proximal AER cells can enable the formation of a proximal blastema. Further work is required to investigate the relationship between the rule of distal transformation and AER cells.

Benefiting from the stage-dependent regeneration competency in *Xenopus*, our scRNA-seq datasets can discriminate true regeneration responses from injury responses. The majority of limb regeneration-associated genes are derived from salamanders, where an injury control is not necessarily available (as these animals can always regenerate their limbs). We found that many genes associated with salamander limb regeneration (e.g. *Dpt* and *Prdx2*) (Gerber et al., 2018; Haas and Whited, 2017; Leigh et al., 2018) are upregulated upon injury in a subset of fibroblasts, regardless of regeneration competency. In a different context, recent single-cell analysis of mouse digit tip amputations suggests that, independent of the regeneration outcome, some fibroblast populations express blastema-associated genes (e.g. *Prickle1*, *Fbn2* and *Lrrc17*) (Storer et al., 2020). We also see these genes upregulated upon injury in a subset of fibroblasts, but again this response is not specific to regeneration. These results suggest that there may be a conserved response to injury for mesenchymal cells in amphibians and mammals, and may be reflecting early suggestions by Tassava et al. that an injury can induce morphologically assigned ‘dedifferentiation’ that fails to establish a blastema without a specialized wound epidermis (Tassava and Loyd, 1977; Tassava and Mescher, 1975). Indeed, we observed lower levels of some regeneration-associated distal mesenchyme genes (e.g. *Shh*) in the subset of fibroblasts when there are no AER cells (Fig. S10), correlating with regeneration competency. Nonetheless, our results are insufficient to determine: (1) whether the fibroblast cells progressively become intrinsically incompetent to fully dedifferentiate; or (2) whether, without signals from signalling centre potent AER cells, they fail to fully dedifferentiate. Moreover, although a subset of fibroblasts can express genes from multiple lineages, the functional consequences of this gain of transcriptional multipotency and how it resolves during varying stages of regeneration competency remain unclear. Further work on injury-induced mesenchymal plasticity, its interaction with AER cells and cross-species comparison on this topic will be required. Nevertheless, these results underscore that caution is needed when interpreting experiments involving injury (e.g. transplantation), as well as the concern that previously implied ‘regeneration-genes’ may be injury response genes.

Overall, we propose a new cellular model of regeneration incompetency, in which chondrogenesis-associated secreted factors inhibit AER cell formation. Although it remains unclear whether chondrogenesis itself directly inhibits limb regeneration, there are multiple observations from our work and others that support this hypothesis (Dent, 1962; Nye and Cameron, 2005; Wolfe et al., 2000). Our model suggests new avenues for cross-species studies aiming to decipher limb development and regeneration, and can explain why *Xenopus* limb amputations at proximal versus distal sites exhibit different regeneration outcomes, as proximal sites are associated with more advanced stages of chondrogenesis (Dent, 1962; Nye and Cameron, 2005; Wolfe et al., 2000). Furthermore, the pace of chondrogenesis may have an association with limb regeneration ability across species, such that terrestrial warm-blooded animals may have a more robust and fast-paced chondrogenesis programme compared with regeneration-competent aquatic cold-blooded animals. Indeed, limb regeneration-incompetent species such as chicken or mouse have a faster limb chondrogenesis programme during their development compared with regeneration-competent axolotl and *Xenopus*. Additionally, although a side-by-side comparative study would be required, mice bone fractures have been documented to heal faster compared with those in axolotl (Hutchison et al., 2007; Vortkamp et al., 1998). It is well established that chondrogenic programmes are heavily influenced by BMP pathway activity. The ratio of BMP agonist/antagonist (e.g. BMP4/NOGGIN) during development, injury or upon limb amputation may be different between limb regeneration-competent and -incompetent animals. This difference may also be connected to observed *Noggin* phenotypes across species. Specifically, adding exogenous *Noggin* results in extended AER maintenance in chicken (Pizette and Niswander, 1999) and mouse (Wang et al., 2004), whereas it abolishes AER in *Xenopus* (Jones et al., 2013). Targeted comparative studies on these topics will be the subject of future work.

It remains unclear how our identified cellular mechanisms are associated with robust regenerative abilities of some salamanders. Based on current results, regeneration-competent axolotls are suggested to not have a developmental AER (Purushothaman et al., 2019), but can form AEC. Meanwhile AER-associated FGFs are expressed in axolotl mesenchyme (Purushothaman et al., 2019). Hence, it is tempting to speculate that limb regeneration-competent salamanders could withstand inhibitory secreted factors because of the location and, potentially, higher absolute amount of AER cell signals in mesenchymal rather than epidermal cells. Additionally, in contrast to axolotl limb regeneration, where a more homogenous mesenchymal transcriptional response was suggested (Gerber et al., 2018), we identified that only a subset of fibroblast populations can gain transcriptional multipotency and express genes associated with blastema. Whether these differences between species result in more robust regenerative abilities requires further work.

**Fig. 6.**
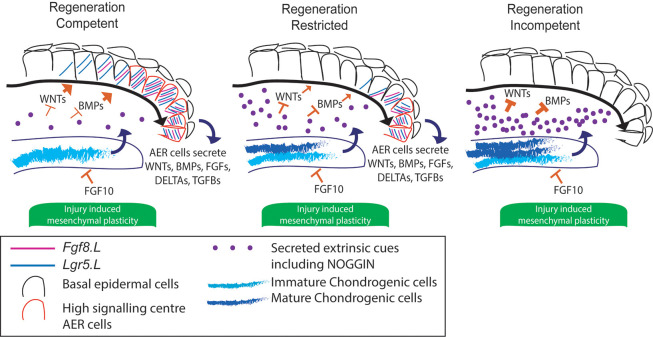
**Schematics describing the proposed model: secreted inhibitory factors associated with chondrogenic progression block AER cell formation.** Secreted factors such as WNTs and BMPs support AER cell formation at the amputation plane. During development, chondrogenesis leads to the accumulation of secreted inhibitory factors, including NOGGIN, which results in failure to establish AER cells (*Fgf8.L+/Lgr5.S+*)*.* FGF10 can suppress chondrogenesis. Amputations, independent of the regeneration outcome, induce injury-induced mesenchymal transcriptional plasticity.

Finally, in this work we have identified a cellular mechanism governing regeneration incompetency in developing tadpoles, although it remains unclear whether similar principles apply in adult frogs with a more definite limb. Manipulation of chondrogenic programmes in adult frogs and other regeneration-incompetent species may lead to novel approaches to promote limb regeneration, albeit with additional barriers to regeneration (e.g. scarring and more complex immune responses) that may have to be overcome. Altogether, our work suggests a new cellular model of limb regeneration (Fig. 6), which unites disparate tissue and gene level findings in the field, and suggests that modulation of secreted factors impacting on epidermal populations has the potential to unlock the ability to regrow lost limbs in non-regenerative higher vertebrates.

## MATERIALS AND METHODS

### Tadpole generation and husbandry

Tadpoles were generated and staged as previously described (Aztekin et al., 2019). After NF stage 45, tadpoles were fed once or twice a day with filamentous blue-green algae (ZM spirulina powder) suspended in water. Wild-type *Xenopus laevis* were used for experiments unless otherwise stated. Tadpoles classified as regeneration competent were NF stage 52-53, regeneration restricted were NF stage 55-56, and regeneration incompetent were NF stage 58-60. Animal experiments were approved by the University Biomedical Services at the University of Cambridge and complied with UK Home Office guidelines and the Animals (Scientific Procedures) Act 1986.

### Single-cell dissociation, library preparation and sequencing

For developmental samples, tadpoles were killed and samples were collected at the aforementioned stages. For amputation/regeneration samples, tadpoles were anaesthetized by incubating them with 0.1×MMR 0.002% MS222 (A0377876, Acros Organics), placed on a wet towel and the right hindlimbs were amputated at the presumptive knee/ankle level for regeneration-competent tadpoles, and at the ankle level for regeneration-restricted or -incompetent tadpoles. Afterwards, the tadpoles were returned to fresh water. At 5 days post amputation (dpa), tadpoles were killed and the newly generated tissues on the amputation plane were collected. Contralateral control samples were also collected from these tadpoles, and intact limb buds or autopods, including ankle, were collected. For each scRNA-seq experiment, tissues were collected from a total of 8-10 tadpoles to reduce variance caused by staging differences. Dissociations were performed on a pool of four limbs in an Eppendorf tube with the following protocol. First, the samples were washed with Ca- and Mg-free 1×MBS (Barth-HEPES saline 10× stock: 88 mM NaCl, 1 mM KCl, 2.4 mM NaHCO_3_, 0.82 mM MgSO_4_.7H_2_O, 0.33 mM Ca(NO3)_2_.4H_2_O, 0.41 mM CaCl_2_.6H_2_O and 10 mM HEPES with ∼3 ml of 10 N NaOH added to obtain a pH of 7.4 to 7.6). Samples were then incubated with 1× trypsin (Sigma, 59427C) in Ca- and Mg-free 1×MBS with 0.5 μM EDTA for 10 min at room temperature on a bench-top shaker at a speed of 300 rpm. Trypsin reaction was diluted with Ca- and Mg-free 1×MBS after 10 min. Physical dispersion was applied (10-15 times up and down trituration with a pipette) to samples before, half way and at the end of trypsinization. Cells were spun down at 250 ***g*** for 5 min, the supernatant was taken out and cells were then resuspended in 1× Ca- and Mg-free MBS. Cells were passed through a 35 μm diameter cell strainer then stained with 20 μM Hoechst 33342 (Sigma, 2261) in 1× Ca- and Mg-free MBS for 10-15 min; Hoechst positive cells were sorted using a Sony SH800s Cell Sorter. scRNA-seq libraries were generated using 10X Genomics (v3 chemistry) and sequenced in pools of two samples per lane on an Illumina Novaseq 6000 SP flow cell, with the following parameters: 28 bp - read 1; 8 bp - i7 index; and 91 bp - read 2, as per standard 10X Genomics recommendations.

### scRNA-seq: data processing

Output files from 10X Genomics were processed using CellRanger v3.0.2, with sequences mapped to the *Xenopus* laevis 9.1 genome (Xenbase, ftp://ftp.xenbase.org/pub/Genomics/JGI/Xenla9.1/Xla.v91.repeatMasked.fa.gz and ftp://ftp.xenbase.org/pub/Genomics/JGI/Xenla9.1/1.8.3.2/XL_9.1_v1.8.3.2.allTranscripts.gff3.gz). Raw counts were normalized by cell library size, and then converted to TPX (transcripts per 10^4^). Cell calling was performed using CellRanger with default parameters. We further filtered the data according to library size, discarding cells with a total UMI count in the lowest quartile. The main cell types and transcriptional changes remained unchanged if this cell-filtering step is omitted, although the clustering and visualization appears less robust (Fig. S4).

### scRNA-seq: feature selection

Highly variable genes (HVGs) were selected for clustering and visualization as described previously (Aztekin et al., 2019) (Fano factor >65th percentile, mean expression >5th percentile and mean expression <80th percentile). Our initial analysis revealed that visualization and clustering were strongly influenced by cell cycle state (Fig. S2). To further refine the set of HVGs, we performed factor analysis with the aim of removing genes significantly associated with the cell cycle. Specifically, non-negative matrix factorization was performed on the cosine normalized, log2-transformed normalized counts matrix, using k=30 components (R package *nnlm*). Factors were manually annotated according to their expression on the UMAP projection, and by inspection of the highest gene loadings for each factor; two factors corresponded to the cell cycle. To minimize the effect of the cell cycle signature on projection/clustering, we identified genes associated with these cell cycle factors (top 10% gene loadings for each factor) and removed these from the set of HVGs.

### scRNA-seq: visualization and clustering

Data were projected onto two dimensions using the UMAP algorithm (Becht et al., 2019), with log2-transformed HVGs, cosine distance as a similarity measure and parameters k=15, min_dist=0.2. Clustering was performed as described previously (Aztekin et al., 2019). Briefly, we constructed a graph using the UMAP function *fuzzy_simplicial_set* with k=10 nearest neighbours, and then performed graphical clustering using the walktrap algorithm (*cluster_walktrap* from R package *igraph*, with steps=10).

### scRNA-seq: gene set enrichment and cell cycle analysis

Single-cell gene set enrichment scores were calculated with the *AUCell* R package (Aibar et al., 2017), using HVGs as the background gene set. Cell cycle phase was inferred using *CellCycleScoring* (R package *Seurat*) (Butler et al., 2018).

### scRNA-seq: annotation of cell types

Cell type annotation was performed by manually comparing cluster-specific gene expression patterns [computed using *findMarkers* in R package *scran* (Lun et al., 2016)] with known cell type markers from the literature. Many clusters could be assigned to a well-characterized functional cell type (e.g. *Satellite cell*). Other clusters could not be unambiguously identified, but were assigned a broad label together with a numeric identifier (e.g. *Blood 1*). Finally, a few clusters remain unannotated (e.g. *Unknown 1*). Dotplots of key marker genes of each cell type are provided in Fig. S5.

### scRNA-seq: gene expression visualization

Gene expression in individual cells is visualized on the UMAP projection with points coloured according to expression level (log10-transformed). Gene expression across groups of cells (e.g. for different clusters or for different stage tadpoles) is shown using dotplots coloured by mean expression (log10-transformed, normalized to group with maximal expression). We can detect alleles from both the large (*Gene.L*) or short (*Gene.S*) chromosomes present in the pseudotetraploid *Xenopus laevis* genome*.* In some figures, we report expression from both the large and short allele; in others, we report whichever allele has higher expression for brevity. Differentially expressed genes were identified using the *findMarkers* function (using default parameters, and comparing cells from different conditions); results were then visualized as volcano plots.

### Regeneration assay and bead experiments

Affi-gel blue gel beads (BioRad, 1537301) were incubated with the following proteins overnight at 4°C: 2-3 μg rabbit-IGG isotype control antibody (ab37415); 2-3 μg anti-NOGGIN antibody (ab16054); 0.1% BSA; 1 μg recombinant human FGF10 (R&D, 345-FG) in 1-2 μl 0.1% BSA; and 1-1.5 μg recombinant human FGF10 (R&D, 345-FG) and 2.5-4 μg recombinant human NOGGIN (R&D, 6057-NG) in 3-4 μl 0.1% BSA. Tadpoles were anaesthetized with 0.002% MS222, placed on a wet towel, and both right and left hindlimbs were amputated from ankle level in either regeneration-restricted or -incompetent tadpoles. Three or four beads were placed on the amputation plane of the right hindlimb. Left hindlimbs served as an internal control for the experiments. Pushing the bead deep in the tissues at the amputation site was avoided as much as possible, and beads were gently positioned instead. Tadpoles were monitored on a wet towel for 3-5 min then tadpoles that retained the beads were placed in fresh water. Tadpoles were killed in between 18 and 21 dpa to assess the regeneration outcome. The difference in the number of digits or digit-like structures between the right to the left limb was quantified for each tadpole.

### Whole-mount mRNA visualization and hybridization chain reaction (HCR), with or without a combination of immunofluorescence or histology

#### HCR on whole-limb or tail samples

HCR was applied as described previously (Choi et al., 2018) with modifications, and materials for HCR were purchased from Molecular Instruments unless otherwise stated. Limb and tail samples were fixed with 4% formaldehyde in 1×PBS for 40-60 min, permeabilized in 70% ethanol in 1×PBS for 2-4 h, washed briefly with 1×PBS and collected in Eppendorf tubes. These procedures were carried out on a rotator at room temperature. The supernatant was taken out, 500 μl wash solution was added, and samples were rotated at room temperature for 5 min. The supernatant was taken out and replaced by 400-500 μl hybridization buffer for a 30 min incubation at 37°C. In parallel, the probe solution was prepared by diluting mRNAs targeting probes to 30-40 nM in 200 μl hybridization buffer and incubated for 30 min at 37°C. The hybridization buffer from samples were taken out and probe solution was placed on samples for a 12-16 h incubation at 37°C. Subsequently, the samples were washed twice for 20 min with wash buffer and twice for 30 min with 5×SSC-T at room temperature. To visualize probes, amplification solution was prepared by first heating the fluorophore attached hairpins pairs (h1 and h2 hairpins) that match to the probes to 95°C for 90 s. Hairpins were then left in the dark at room temperature for 30 min. Afterwards, final amplification solution was prepared at 40-60 nM h1 and h2 in 200 μl amplification buffer. Samples were first incubated in amplification buffer without hairpins for 10 min, then placed in final amplification solution at room temperature, protected from light, for 12-16 h on a rotator. Samples were washed with 2×20 min SSC-T. Samples were then stored in 1×PBS.

#### Whole-mount HCR samples imaging

For stereomicroscope or confocal imaging of whole samples, the samples were mounted in 0.6-0.8% ultra-low gelling temperature agar (Sigma, A5030) in 1×PBS.

#### Sectioning of samples after HCR

In the subsequent step of the protocol, the samples were protected from light to preserve the HCR signal. The samples were incubated in 15% sucrose in 1×PBS at room temperature for 1 h, then 30% sucrose in 1×PBS at 4°C overnight. Samples were then placed in OCT solution and incubated at −80°C overnight. Samples were cryosectioned at 5 μm, stained with 20 μM Hoechst (Sigma, 2261) in 1×PBS at room temperature for 10 min and imaged.

#### Immunostaining

After sectioning of HCR stained limb, the samples were processed for immunostaining. Samples were blocked with 50% Cas-Block (Invitrogen, 008120) in 1×PBS-T (1X PBS+0.1 Tween-100) and incubated for 30 min at room temperature without rotating. Samples were then incubated with antibodies (listed below) at 4°C overnight without rotating. Samples were washed with PBS-T for 2×10 min, blocked with 50% Cas-Block in 1×PBS-T for 30 min, and incubated with secondary antibodies (listed below) for 1 h. All these steps were carried out at room temperature without rotating. Samples were washed with 1×PBS-T twice for 10 min and with 1×PBS twice for 20 min at room temperature without rotating. After antibody staining, samples were stained with Hoechst and washed once for 5 min in 1×PBS at room temperature without rotating. Samples were mounted in 80% glycerol in 1×PBS with a coverslip and imaged.

Tail whole-mount HCR staining can be combined with whole-mount immunofluorescence by following the above immunofluorescence protocol, except that the mounting of whole tails were carried out in ultra-low gelling temperature agar for imaging.

##### HCR probes and hairpins

Probes for *Fgf8.L*, *Dpt.L*, *Htra3.L*, *Prrx1.L* and *Sp9.L* were purchased from Molecular Instruments. Probes were designed against the full-length *Xenopus Lgr5.S*, *Msx1.L* and *Fgf10.L* mRNA sequence as described previously (Choi et al., 2014). HCR hairpins were purchased from Molecular Instruments.

##### Primary antibodies and working dilutions

Primary antibodies and working dilutions were as follows: TP63 [4A4] (Abcam, ab735, 1:200), B-CATENIN (Abcam, ab6302, 1:2000), E-CADHERIN (5D3, DSHB, 1:10), ITGB1 (8C8, DSHB, 1:10) and anti-EGFP (Abcam, ab13970, 1:500).

##### Secondary antibodies

Secondary antibodies were as follows: goat anti-chicken IgY (H+L) secondary antibody, Alexa Fluor 488 (Invitrogen, A11039, 1:500), goat anti-mouse IgG (H+L) cross-adsorbed ReadyProbes secondary antibody, Alexa Fluor 594 (Invitrogen, R37121, 1:500) and goat anti-mouse IgG (H+L) cross-adsorbed ReadyProbes secondary antibody, Alexa Fluor 488 (Invitrogen, R37120, 1:500).

A Leica SP8 upright confocal microscope with a 40×/1.3 HC PL Apo CS2 Oil objective was used for all confocal images except for Fig. S9B,C, which were taken with a Leica SP8 inverted confocal microscope with a 20×/0.75 HC PL Apo CS2 Multi. LAS X was used for setting tiled images and a 20% overlap between tiles was used. Limb whole-mount HCR images were taken via a Leica stereomicroscope equipped with a DFC7000T camera. Fiji was used for maximum projection of *z*-stacks and to adjust contrast to highlight biological relevance. If needed, images were cropped, flipped and/or rotated to highlight biological relevance.

Histological staining can be carried out on cryosectioned HCR samples. Briefly, samples were stained with Hematoxylin and Eosin according to manufacturer's protocol (Abcam, ab245880) then samples were stained for Alcian Blue (Sigma, B8438) according to manufacturer's protocol. Histology images were captured on a Zeiss AxioImager compound microscope.

### *Ex vivo* limb culture method to assess AER cell formation and proximal chondrogenesis

Limbs were first amputated from presumptive knee/ankle level for regeneration-competent and from ankle level for regeneration-restricted or -incompetent tadpoles. The distal parts of these amputated explants were then removed and the remaining proximal segment was placed in 1000, 500 or 200 μl explant media [L-15 (ThermoFisher Scientific, 21083027), 1×Antibiotic-Antimycotic (ThermoFisher Scientific, 15240062) and 20% Fetal Bovine Serum Superior (Sigma, S0615)] in 12, 24 or 96-well plates, respectively. Explants were cultured for 3 days without changing the media. After 3 days, to quantify AER cell formation, the explants were fixed and proceeded to the HCR protocol; to quantify proximal chondrogenesis, the explants were fixed with 4% formaldehyde, mounted in 0.6% Low-Melt agar and directly imaged by stereomicroscopy. Explants emit autofluorescence. Although the abundant HCR signal can be seen despite the autofluorescence, to discriminate the HCR signal from autofluorescence in finer detail, sample images were taken in red and green channel separately with the same exposure and gain settings, and then merged in Fiji. In merged images, the background signal due to autofluorescence was visualized as yellow and the HCR signal was either red or green. As AER cells were largely detected as a monolayer population, AER cell formation was calculated by measuring the length of the *Fgf8.L* signal on the amputation plane using Fiji segmented line option. The proximal chondrogenesis can be visually distinguished and, to determine the chondrogenesis length, chondrogenic structure length from top to bottom was also measured using Fiji. Samples where a clear chondrogenesis was not visible were omitted from further analysis. These images were taken in bright field and measurements were carried out in Fiji.

For drug and recombinant protein treatments, the explants were placed in culture media containing the following small molecules concentration or recombinant protein amounts, unless otherwise stated: 100 μM ICRT3 (Sigma, SML0211), 100 μM SU-5402 (Sigma, SML0443), 50 μM SB-505124 (Sigma, S4696), 100 μM DAPT (Sigma, D5942), 2.5 μM LDN-193189 (Stemgent, 04–0074), 500 ng human recombinant FGF10 (R&D, 345-FG), 1.25 μg human recombinant NOGGIN (R&D, 6057-NG) and 500 ng human recombinant BMP4 (R&D, 314-BP). Drugs were prepared in DMSO, and recombinant proteins were prepared in 0.1% BSA. Small-molecule experiments were conducted in 24-well plates. Recombinant protein experiments were carried out in 96-well plates. A maximum of six explants were placed in 24-well plates. One explant was put in one well of 96-well plate for recombinant protein treatments. In all chemical and recombinant protein perturbation experiments, one limb of the same animal was subjected to the perturbation, and the contralateral limb served as a control. These control explants were exposed to solution containing matching DMSO or BSA concentration in 1×PBS for chemical or recombinant protein perturbations, respectively. Perturbation and control samples were pooled separately at the end of the experiments and stained.

### EdU labelling

*Ex vivo* limbs were cultured with 10 μM EdU (ThermoFisher Scientific, C10337) for 3 days in dark foiled cover. Afterwards, samples were fixed and *Fgf8.L* mRNA was stained using the HCR protocol, followed by cryosectioning, as described above. Sections were subjected to the Click-It reaction, as described in the manufacturer's protocol (ThermoFisher Scientific, C10337). Hoechst was added at the end of the protocol. Samples were visualized by confocal microscopy as described above. *Fgf8.L*-positive cells, and both EdU- and *Fgf8.L*-positive cells on the amputation plane were manually counted, and the percentage of EdU-positive *Fgf8.L*-positive cells were calculated for each sample.

### Bead experiment for proximal chondrogenesis

Beads were prepared as described above. Explants from regeneration-restricted tadpoles were harvested as described above and beads were implanted on the proximal site of explants. At 3 dpa, explants that no longer contained a bead at their proximal site (presumably due to repulsion) were omitted from further analysis. At 3 dpa, samples were imaged without fixation and the extent of chondrogenesis was measured by Fiji.

### DiO labelling

DiO [DiO′; DiOC_18_(3) (3,3′-dioctadecyloxacarbocyanine perchlorate), ThermoFisher Scientific, D275] was prepared by dipping a tip in the DiO-containing powder tube and placing the tip in a 10 μl of 96-100% ethanol in an Eppendorf tube. A glass needle tip was then dipped in the diluted DiO solution and harvested *ex vivo* limbs were labelled on a wet towel. These cultures were placed in *ex vivo* culture media and explants were imaged every day with a stereomicroscope.

### *Ex vivo* limb co-culture, and conditioned media experiments

For co-culture experiments, one regeneration-competent and one regeneration-incompetent limb explant were incubated together in 200 μl explant media in one well of 96-well plate. For antibody experiments, one limb of each animal served as a control and was incubated with 1 μg rabbit-IGG isotype control antibody (ab37415), while the contralateral limb was incubated with 1 μg anti-NOGGIN antibody (ab16054). Antibodies and media were added only at the beginning of the cultures and were not replaced during the experiment.

For conditioned media experiments, conditioned media supplying and receiving explants were prepared separately. Supplying explants were prepared 1 day before harvesting receiving explants and incubated in 200 μl explant media in one well of 96-well plate. After 1 day, media from the supplying explant was collected and used to culture the newly harvested receiving explant, and fresh media were added for supplying explant. This change of media procedure was repeated for 3 days. For antibody experiments, supplying explant media was collected and pre-incubated with 1 μg antibodies for 25-30 min at room temperature on a rotator, then the pre-incubated media was placed on the receiving explants.

### Replicate information and statistical tests

Sample sizes were not pre-determined in any experimental setup. In this work, biological replicates refer to samples obtained from multiple animal batches and to experiments carried out on different days. In all experiments, the number of independent tadpole limbs assayed is recorded and denoted by *n* in the text and figure legends*.* In all experiments, wild-type tadpoles were used from tanks that contain multiple batches (tadpoles raised from different father and/or mother). In all explant perturbation experiments, samples were compared with their contralateral controls and a Mann–Whitney *U*-test was used to determine statistical significance. For regeneration and bead experiments, a *t*-test was used. To assess the significance of proximal *Fgf8* expression in explants (Fig. 5F), Fisher's exact test was used.

## Supplementary Material

Supplementary information

Reviewer comments
