# Supplementary material for: Secreted inhibitors drive the loss of regeneration competence in Xenopus limbs
Source: Development. 2021 Jun 9;148(11):dev199158. doi: 10.1242/dev.199158 (PMC8217717; doi:10.1242/dev.199158)
Supplement: Supplementary information [file develop-148-199158-s1.pdf]

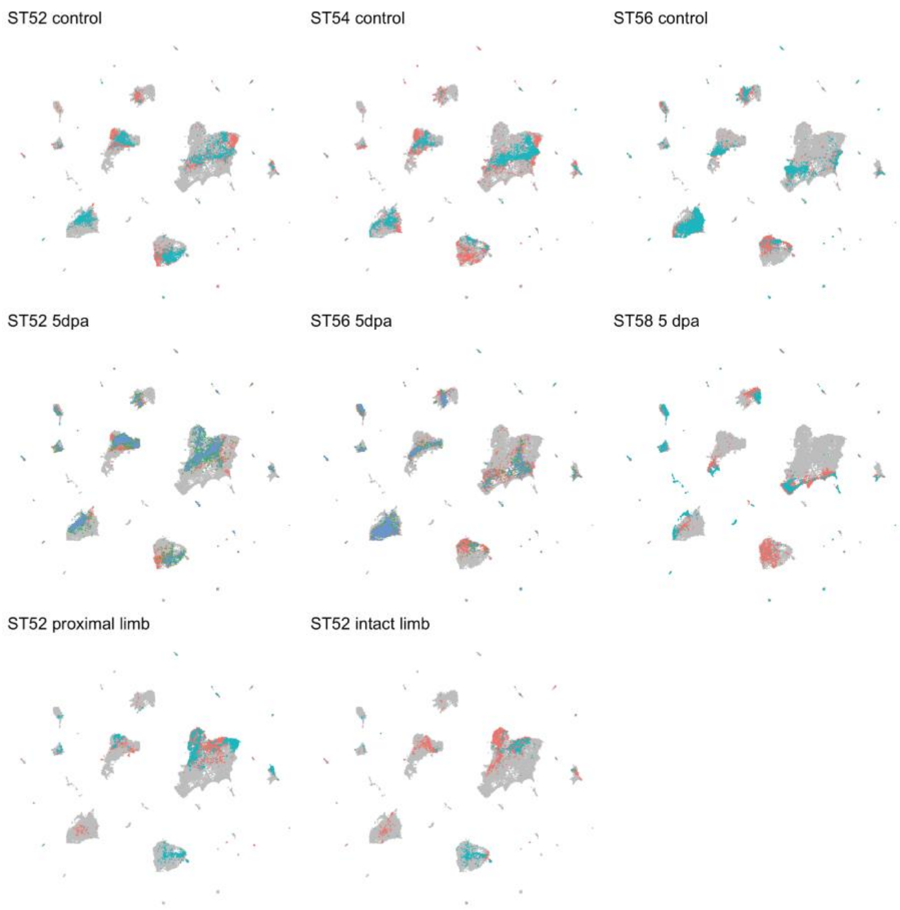

Figure S1: Contribution of different conditions to the pooled UMAP projection. UMAP visualization of cells from all conditions and replicates, allowing the identification of transcriptional changes that are consistent across replicates. Grey dots: cells from all samples; red, blue, green dots: cells from different biological replicates for the selected sample.

Development • Supplementary information

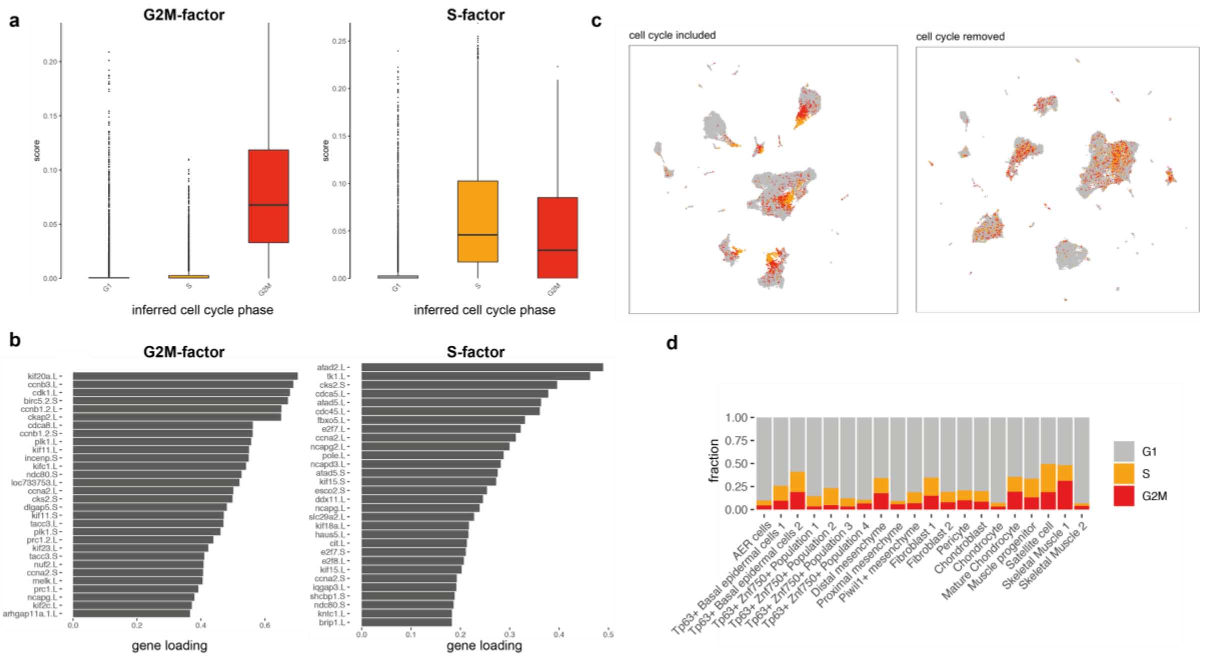

Figure S2: Detection and removal of the cell cycle signature.

(**a**) Unbiased factor analysis identified two factors that correspond to computationally-inferred cell cycle phases (G2M-factor, left; S-factor, right). (**b**) Factor loadings for the top 30 genes associated with cell cycle factors. (**c**) Removal of genes with high loadings for either G2M- or S-factors significantly reduces the influence of cell cycle phase on the UMAP projection. Dot color indicates inferred cell cycle phase. (**d**) Inferred cell cycle states for selected cell types.

Development • Supplementary information

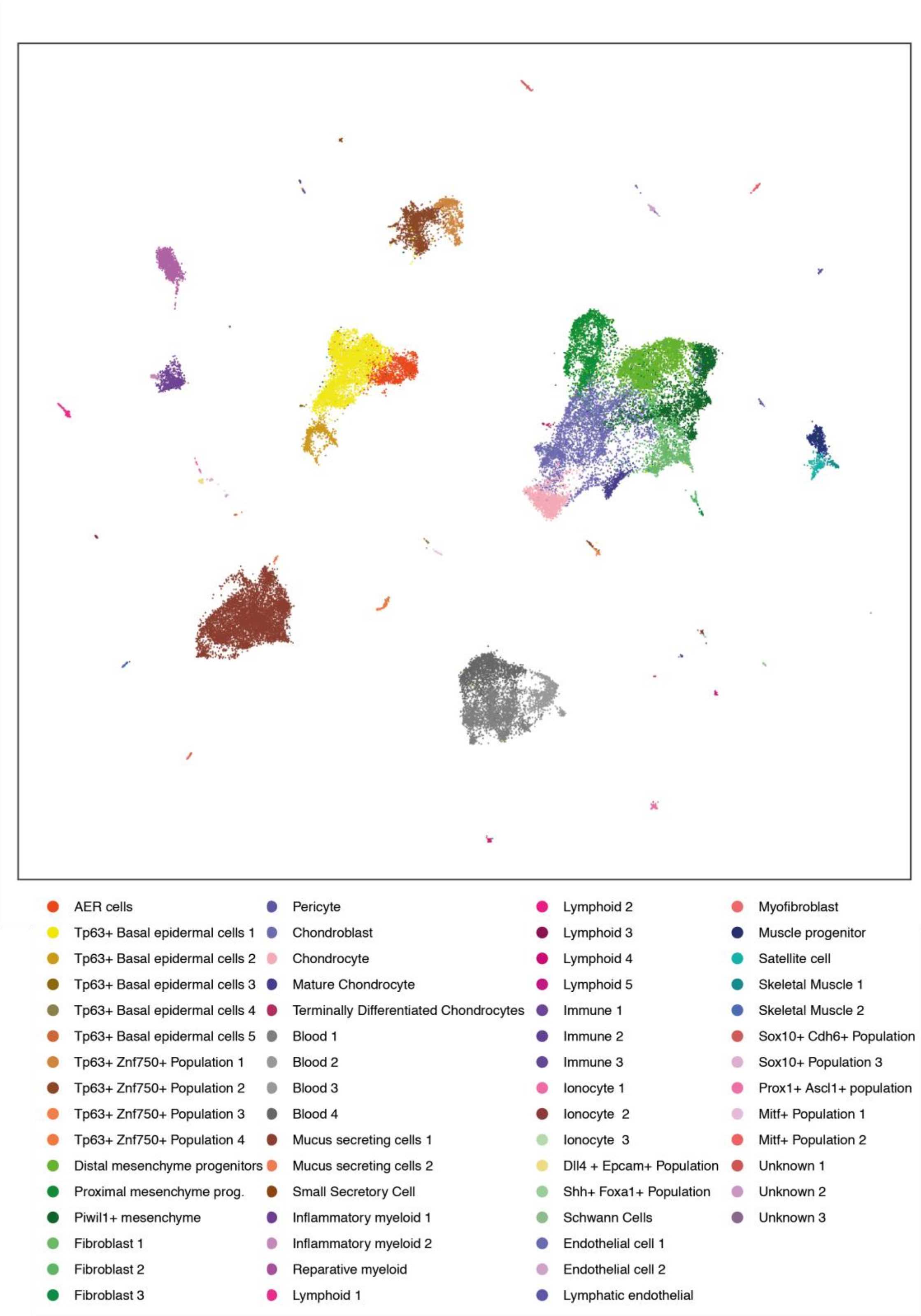

Figure S3: An atlas of cell types in developing and amputated limbs at different stages of regeneration-competence.

Pooled UMAP visualization of *Xenopus* limb cells, with colors representing distinct cluster identities.

Development • Supplementary information

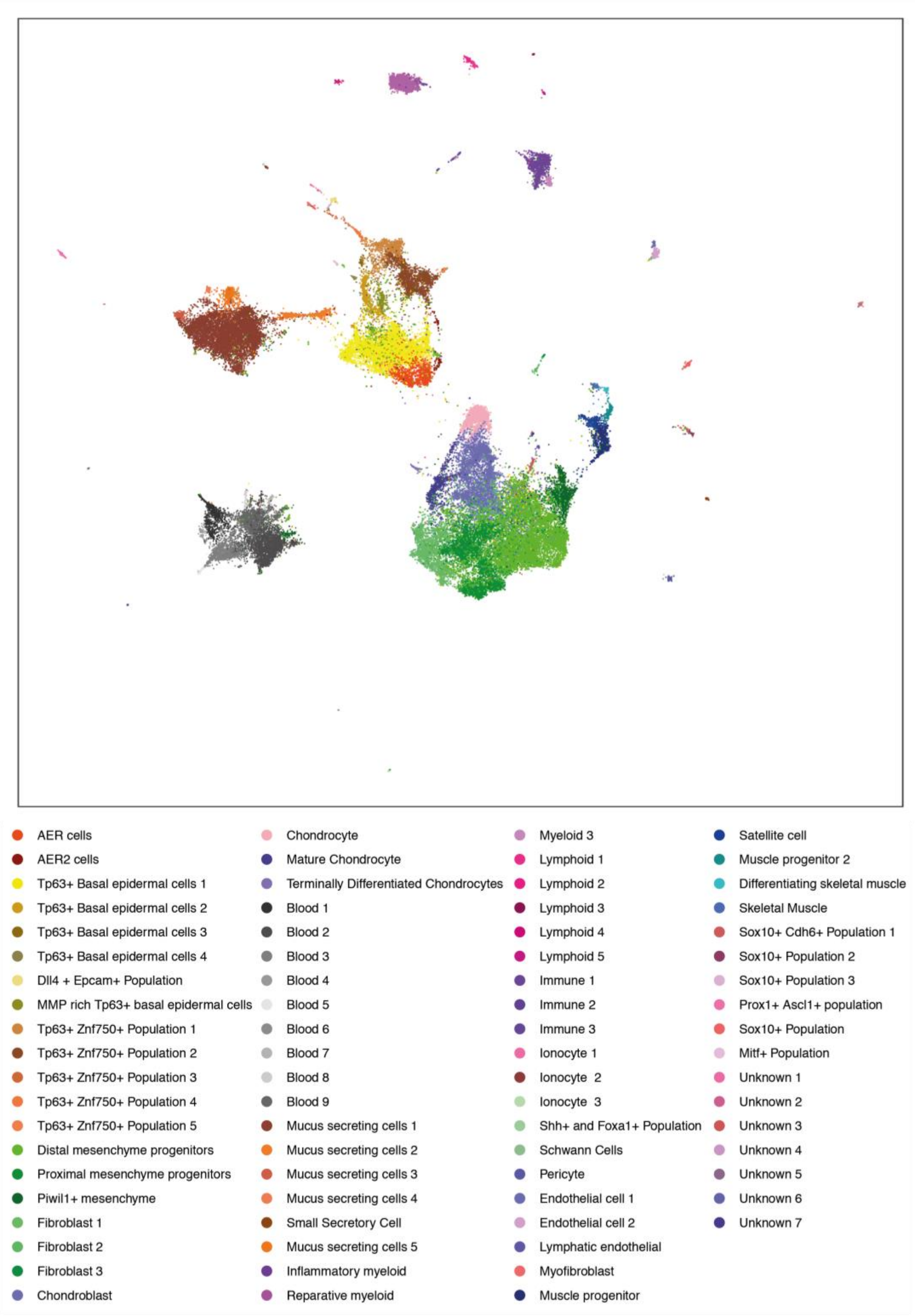

Figure S4: An expanded atlas of the *Xenopus* limb using less stringent cell filtering protocols.

Pooled UMAP visualization and clustering of all barcodes that are identified as cells using cellRanger with default parameters. The majority of transcriptional states are similar to Figure S3, although a fraction of low-UMI mesenchymal cells appear mislocalized across the atlas.

Development • Supplementary information

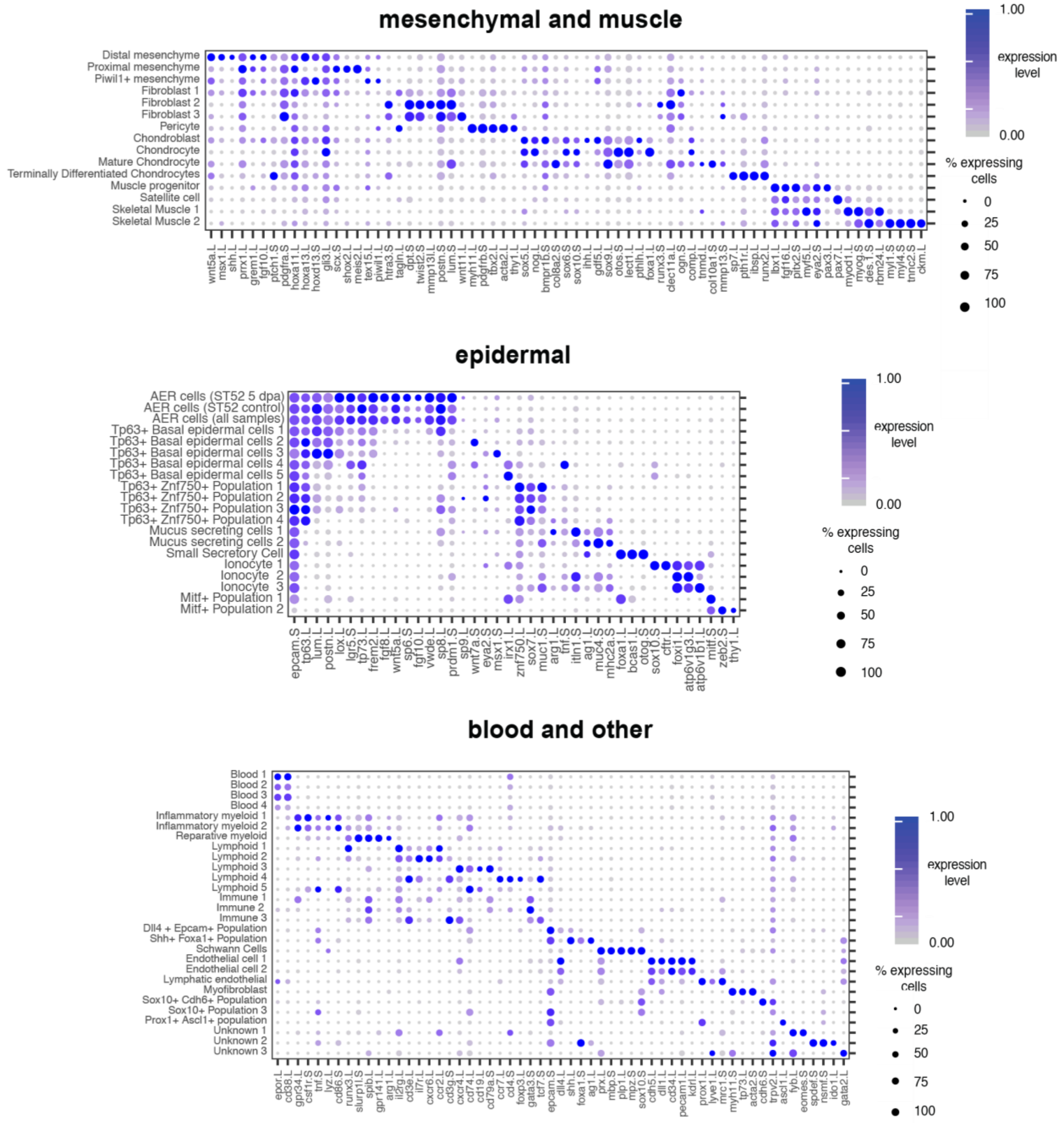

Figure S5: Annotation of cell types using known markers of cell identity.

Dotplots showing marker genes for each of the 61 cell types in our atlas. For ease of presentation, we group cell types into three broad categories: mesenchymal and muscle (top), epidermal (middle), blood and other (bottom). Please note that for the AER cell cluster, we also provide marker gene expression for specific samples as indicated. Dot color denotes mean expression level within the cluster; dot size denotes the percentage of cells within the cluster with non-zero expression.

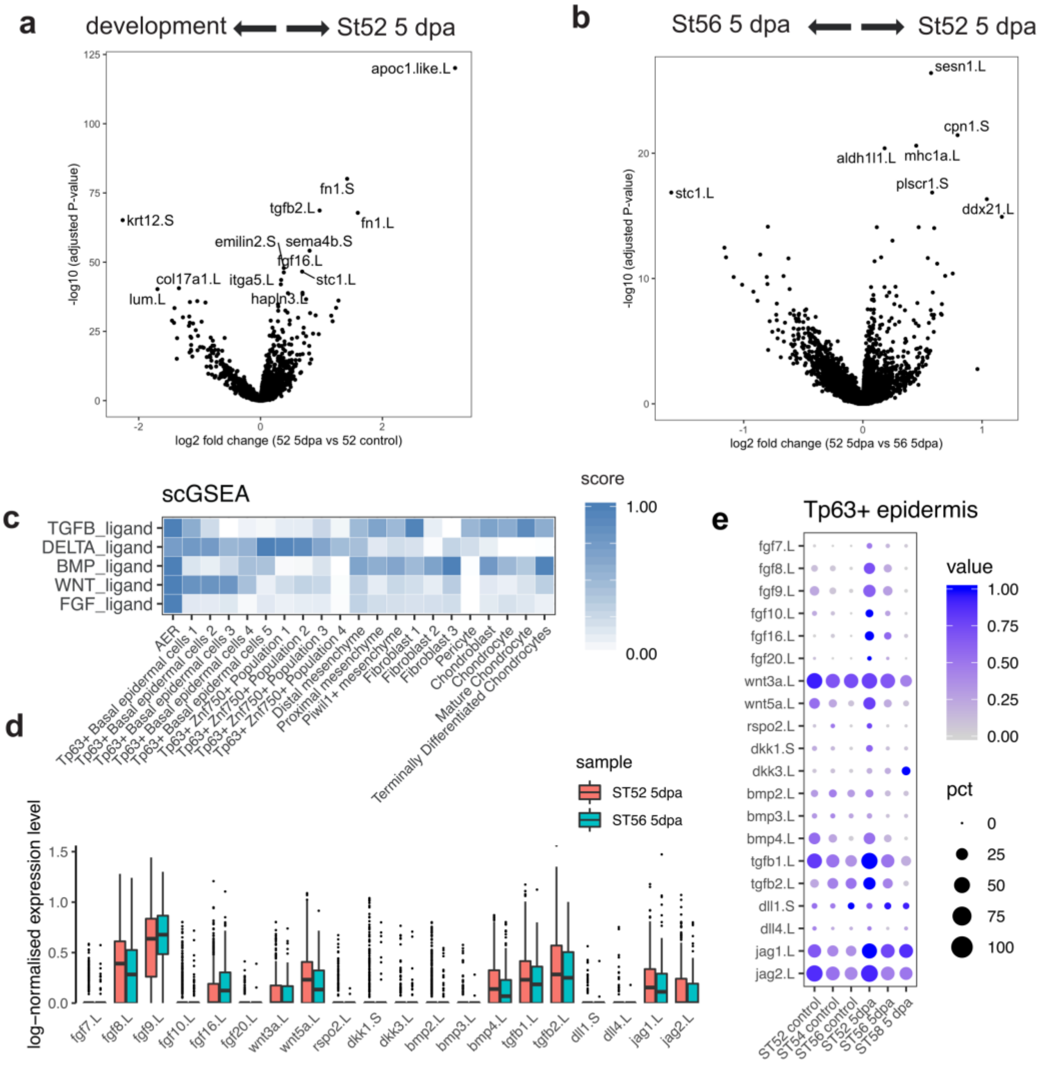

Figure S6: AER cells are a signaling center population.

Differentially expressed genes are detected in AER cells between pairs of conditions and visualised using volcano plots. In **(a)**, Stage 52 5 dpa is compared to Stage 52 control samples; in **(b)**, Stage 56 5dpa is compared to Stage 52 5dpa samples. **(c)** Heatmap showing single-cell gene enrichment scores for ligands from the main signaling pathways are shown for epidermal cell types. AER cells have high signal center properties as they express high levels of TGF-β, Delta, BMP, WNT, and FGF ligands. Please see Supplementary Table 2 for the full list of ligands used in this analysis. **(d)** Log10-normalized gene expression visualised using boxplots to compare expression levels between Stage 52 5 dpa and Stage 56 5 dpa AER cells. **(e)** Dot plot showing expression of AER cell associated selected ligands for TP63+ epidermal cells during development and at 5 dpa in regeneration-competent, -restricted, and –incompetent samples. Dot color indicates mean expression; dot size represents the percentage of cells with non-zero expression."

Development • Supplementary information

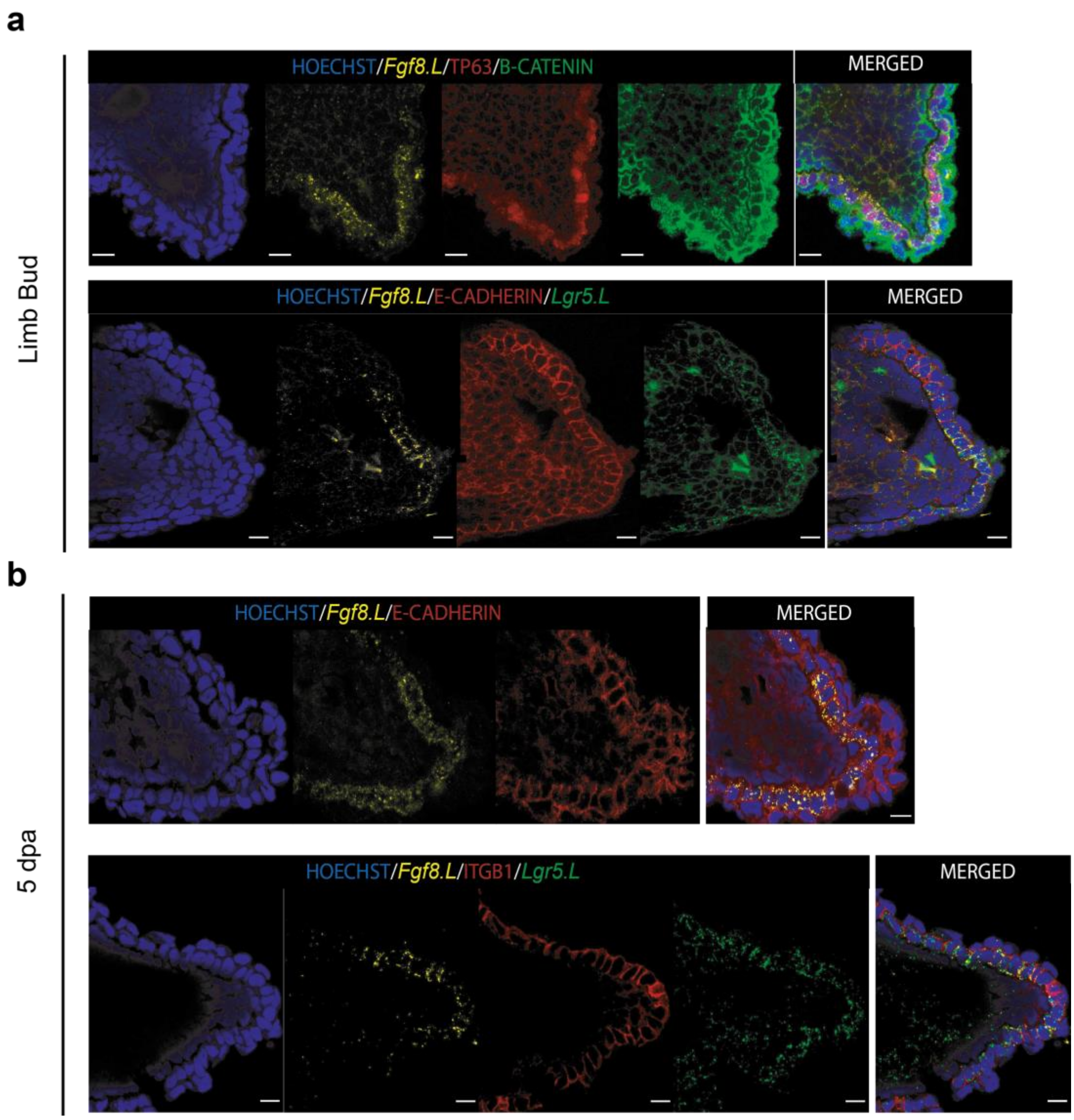

Figure S7: AER cells are largely found as cuboidal monolayer cells showing apical-basal polarity.

AER cells were visualized during limb development **(a)** and at 5 dpa **(b)** in regeneration-competent tadpoles by labelling *Fgf8.L* mRNA. AER cells are largely present as monolayer cuboidal basal epidermal cells with apical-basal polarity. A simple squamous layer is present above AER cells, and cells with mesenchymal morphology are located underneath AER cells. From the proximal to distal midline of the epidermis, *Lgr5.S* expression is first detected, followed by *Fgf8.L* mRNA expression. Both *Fgf8.L* and *Lgr5.S* are expressed at high levels at the tip of limbs. AER cells show similar cuboidal morphology during development and regeneration. Basal epidermal cells are morphologically similar based on Hoechst and

Development • Supplementary information

membrane markers, and *Fgf8.L* detection is required to detect AER cell. Row 1: Blue, Hoechst; Yellow, *Fgf8.L* mRNA; Red, TP63; Green, B-catenin. Row 2: Blue, Hoechst; Yellow, *Fgf8.L* mRNA; Red, E-Cadherin; Green, *Lgr5.S* mRNA. Row 3: Blue, Hoechst; Yellow, *Fgf8.L* mRNA; Red, E-Cadherin. Row 4: Blue, Hoechst; Yellow, *Fgf8.L* mRNA; Red, ITGB1; Green, *Lgr5.S* mRNA. Scale bars = 10 μm.

Development • Supplementary information

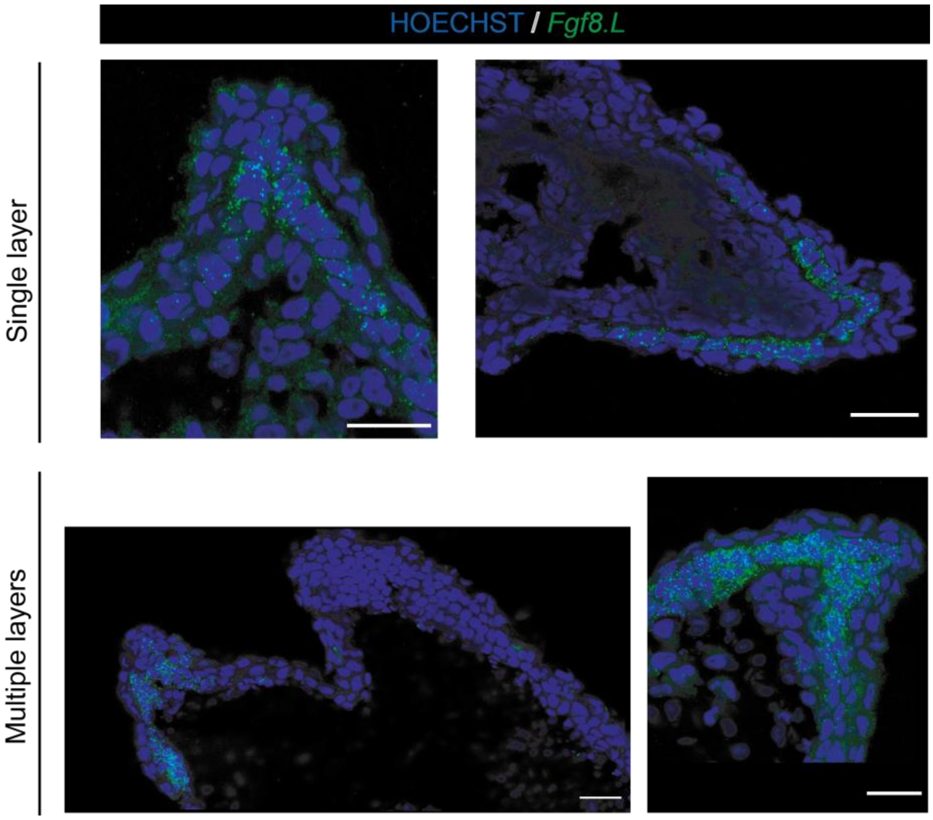

Figure S8: AER cells can be mono- or multi-layered structures.

Fgf8.L images of sectioned *in vivo* 5 dpa samples from regeneration –competent (top) and –restricted (bottom) samples. Morphology of AER cells (*Fgf8.L+*) can vary between sections and samples. Top left, AER cells are seen as single monolayer largely cuboidal although some have higher height to width ratio. Top right, AER cells are seen as single monolayer largely cuboidal cells. Bottom left, AER cells can be seen as multi-layered population that is not covering the whole amputation plane. Bottom right, AER cells can be seen as multi-layered population covering the amputation plane. Blue, Hoechst; Green, *Fgf8.L* mRNA. Scale bars = 25 µm.

Development • Supplementary information

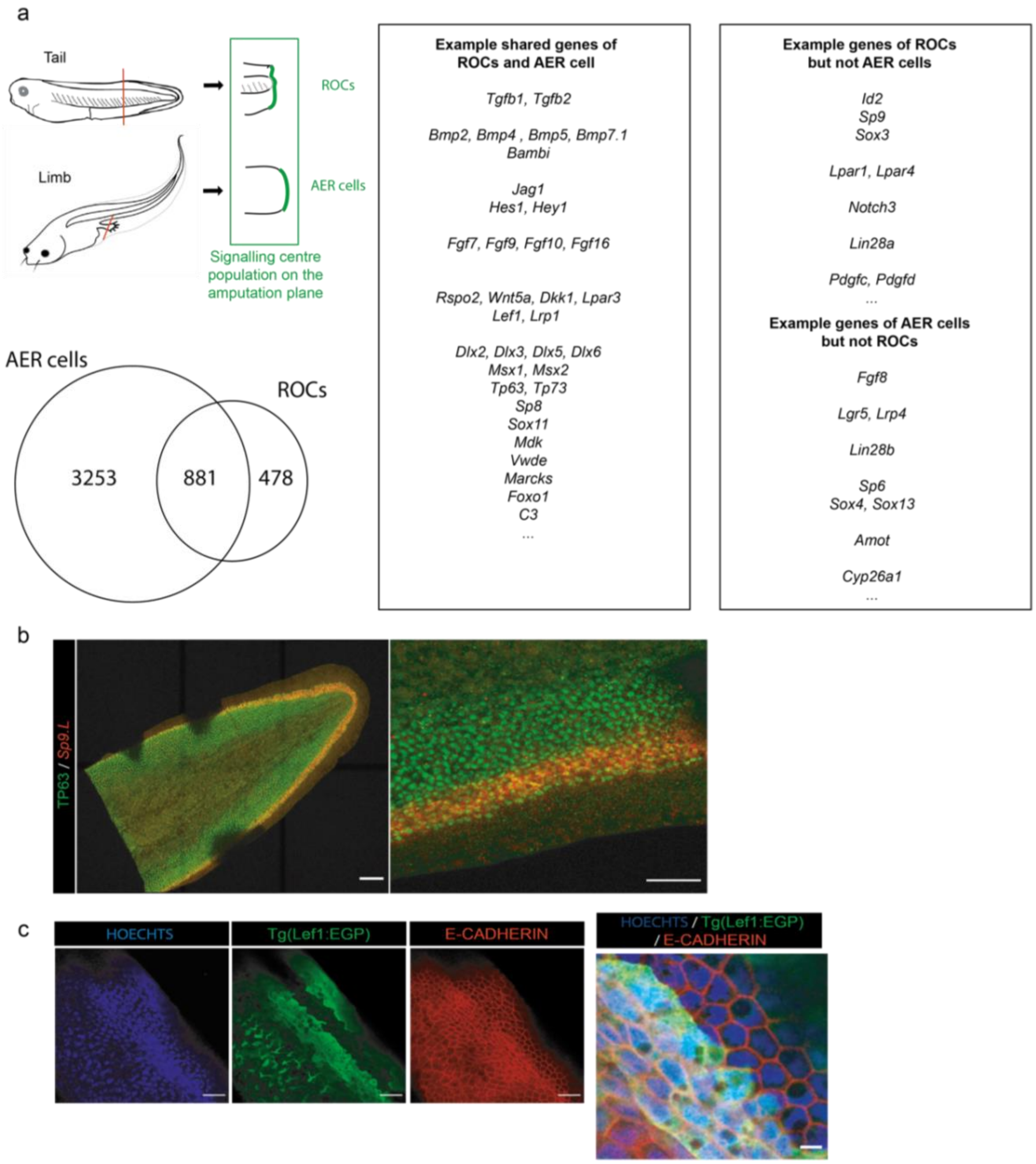

Figure S9: Specialised wound epidermis of tail and limb regeneration share some transcriptional similarities while presenting different cellular morphology.

(**a**) (Left) A signaling center population serving as the specialized wound epidermis is associated with *Xenopus* tail and limb regeneration. However, tail uses regeneration-organizing-cells (ROCs) (Aztekin et al., 2019) while limb uses AER cells for this purpose. Both AER cells and ROCs share the expression of many genes highlighting their similarity, although there are some genes that are unique to each population. AER- and ROC-specific genes were identified as genes significantly upregulated relative to other basal epidermal cells. (Right) A select number of genes, specifically ligands and transcription factors that are

Development • Supplementary information

associated with regeneration, are highlighted. **(b)** ROCs and AER cells show different morphologies (please see **Figure S7** for AER cells). ROCs were visualized by staining NF Stage 40 by *Sp9.L* mRNA expression (highly specific for ROCs (Aztekin et al., 2019)) and TP63 immunolabelling for whole tail (Left) and zoomed in version (Right). In the zoomed in version for staining *Sp9.L* shows two level of expression in ROCs: a single outer layer of *Sp9.L* low cells, and multiple inner layers of Sp9.L high cells. Please note that this is not whole bottom-top image of a tail as evidenced by absence of TP63 staining in the in middle part of the tissue. Red, *Sp9.L* mRNA; Green, TP63. Scale bars= (left) 250 µm, (right) 100 µm. **(c)** ROCs are visualized using the pbin7LEF:GFP line, as defined previously (Aztekin et al., 2019), and E-CADHERIN staining was used to delineate cell shape. Inner layers of ROCs have flattened cell shape while the outside layer ROCs exhibit more square-like shape. ROCs do not have branched nuclei, unlike fin cells. Blue, Hoechst; Green, EGFP; Red, E-cadherin. Scale bars= 10 µm.

Development • Supplementary information

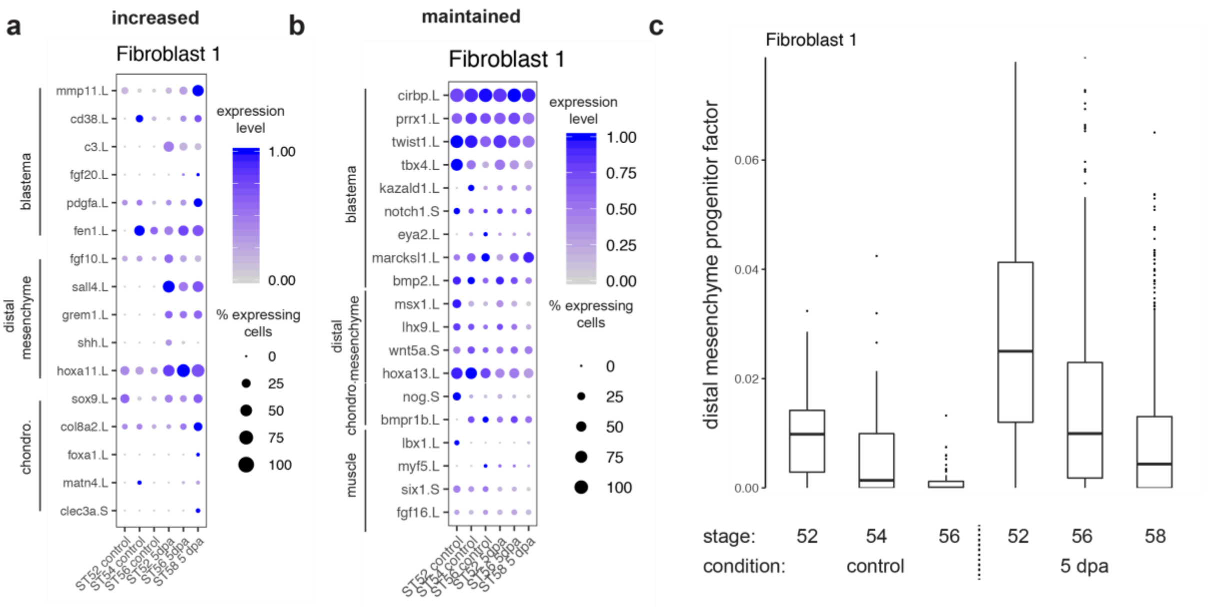

Figure S10: A subset of fibroblasts express dedifferentiation and blastema genes independently of the regeneration-outcome.

Expression of genes and putative gene sets associated with regeneration in the Fibroblast 1 cluster, visualized using dotplots and factor analysis. (**a**) Expression of specific genes that increase upon amputation regardless of stage. (**b**) Expression of specific genes that are expressed in intact limbs and are maintained after injury. (**c**) Following amputation, the putative distal mesenchyme progenitor gene set (factor) increases in Fibroblast 1 cells across all stages.

Development • Supplementary information

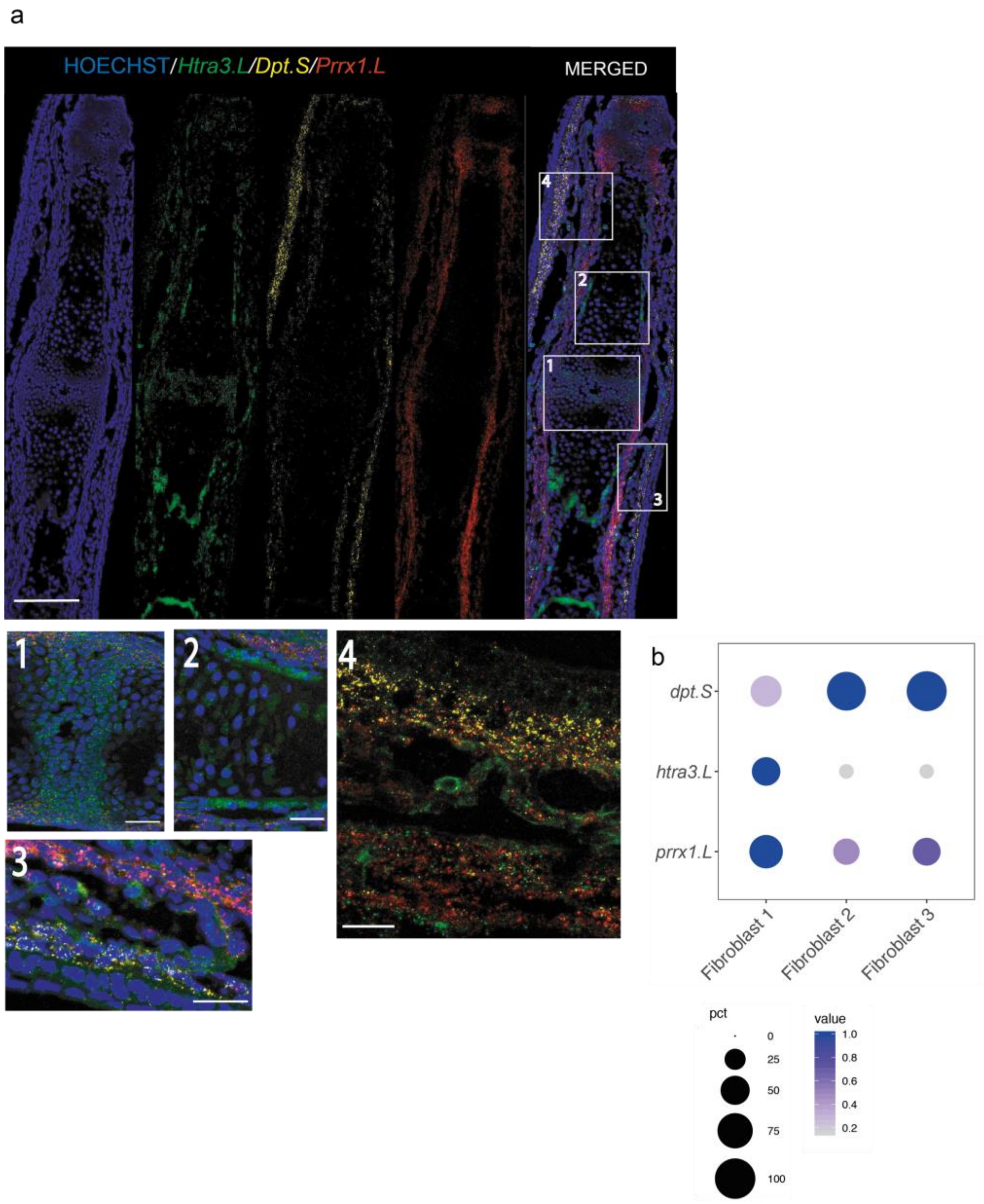

Figure S11: Fibroblast 1 cluster cells are largely found beneath skin cells and nearby perichondrial cells.

(**a**) (Top) Confocal images of a Stage 56 digit stained against *Htra3.L*, *Prrx1.L*, and *Dpt.S*. Cells expressing *Htra3.L*/*Prrx1.L*/*Dpt.S* are found underneath the skin regions and nearby perichondrium regions. (Bottom) Zoomed in version of selected areas show: (1) joint forming regions are enriched for *Htra3.L* expression; (2) Inner perichondrial regions are enriched for *Htra3.L* and outer perichondrial regions are enriched for *Prrx1.L* expression. (3-4) Outerlayers of dermal fibroblast area enriched for *Dpt.S* and lower levels of *Prrx1.L* and *Htra3.L*. Inner layers of dermal fibroblasts/nearby perichondrial regions are enriched for higher Prrx1.L and lower *Dpt.S* and *Htra3.L* expressions. Blue, Hoechst; Green, *Htra3.L* mRNA; Red, *Prrx1.L* mRNA; Yellow, *Dpt.S* mRNA. Scale= 125 μm for top images, 25 μm for bottom no 1-3, and

Development • Supplementary information

20 μm for bottom no 4. **(b)** Dot plot showing expression of *Htra3.L*, *Prrx1.L*, and *Dpt.S* for Fibroblast 1, 2, and 3 clusters.

Development • Supplementary information

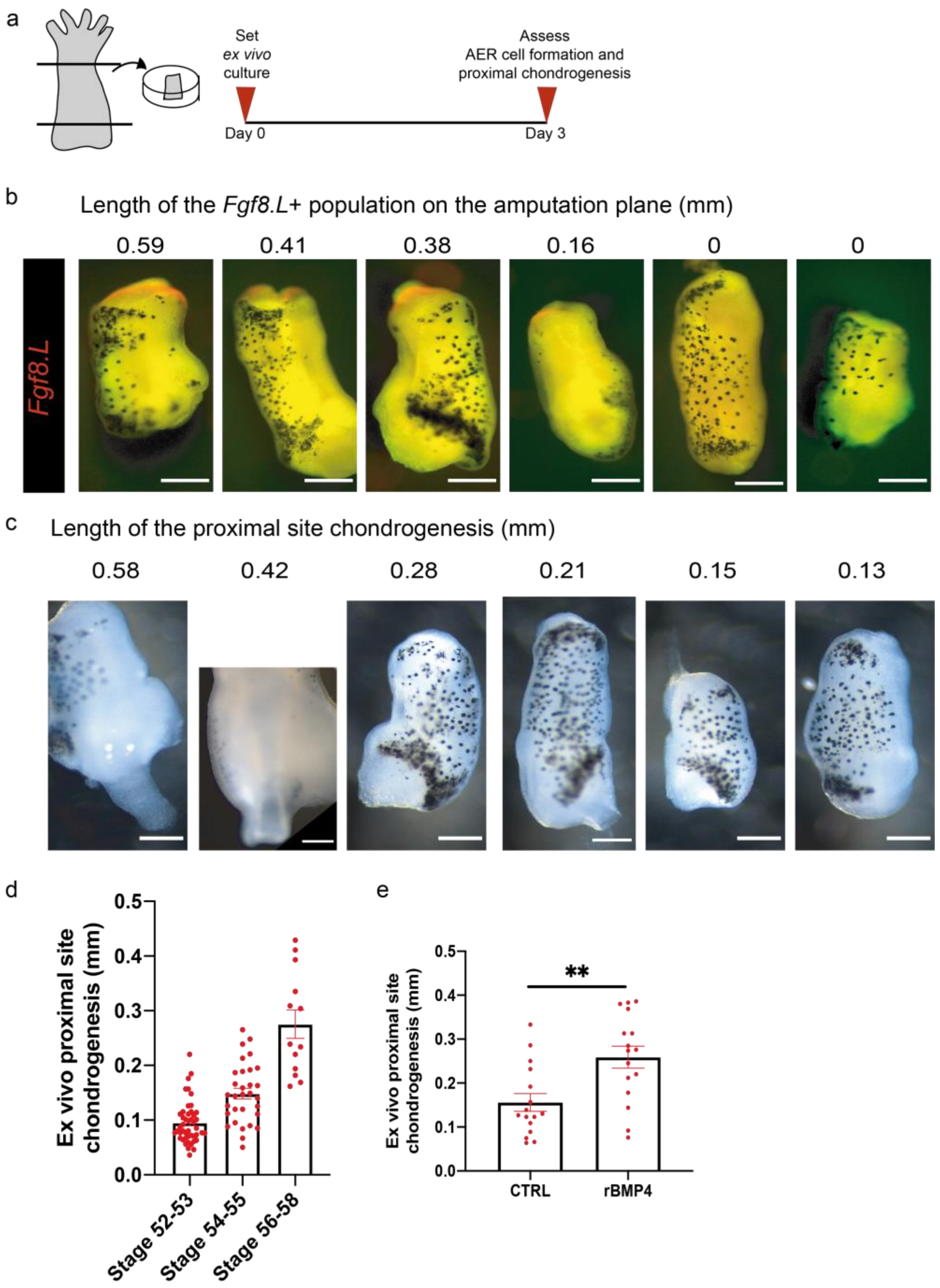

Figure S12: The distal site of *ex vivo* regenerating limbs can be used to detect AER cell formation, and the proximal site of explants can be used for detecting chondrogenesis. **(a)** Schematics describing the *ex vivo* culture protocol for assessing *Fgf8.L* mRNA expression at the distal site, and chondrogenesis levels at the proximal site. All assessments were carried out at 3-days post culture start. **(b)** Whole-mount stereomicroscope images of *Fgf8.L* stained limb explants at 3 dpa. Numbers at the top indicates AER cell formation measured as the length of *Fgf8.L*+ signal on the amputation plane. Red, *Fgf8.L* mRNA. Scale= 200 µm. **(c)** Whole-mount stereomicroscope images of chondrogenesis at the proximal site of explants at 3 dpa.

Development • Supplementary information

Numbers at the top indicates the measured proximal chondrogenesis extent. Scale= 200 µm. **(d)** *Ex vivo* regenerating limb cultures can be used to investigate chondrogenesis. Explants were cultured for 3 days and chondrogenesis was measured as in (c). The extent of chondrogenesis seen at the proximal site of explants changes with the developmental stage and coincides with the progression of *in vivo* chondrogenesis (Dent, 1962). Regeneration-competent explants= total 46 samples from 4 biological replicates; Regeneration-restricted explants= total 31 samples from 3 biological replicates; Regeneration-incompetent explants= total 13 samples from 3 biological replicates. $P^{**} < 0.001$. **(e)** Explants were cultured for 3 days with BMP4 and the extent of chondrogenesis was measured. Addition of recombinant BMP4 to the explant media increased the observed chondrogenesis at the proximal site. Control 0.1% BSA, total n= 16 samples from 4 biological replicates; recombinant BMP4, total n= 16 samples from 4 biological replicates.

Development • Supplementary information

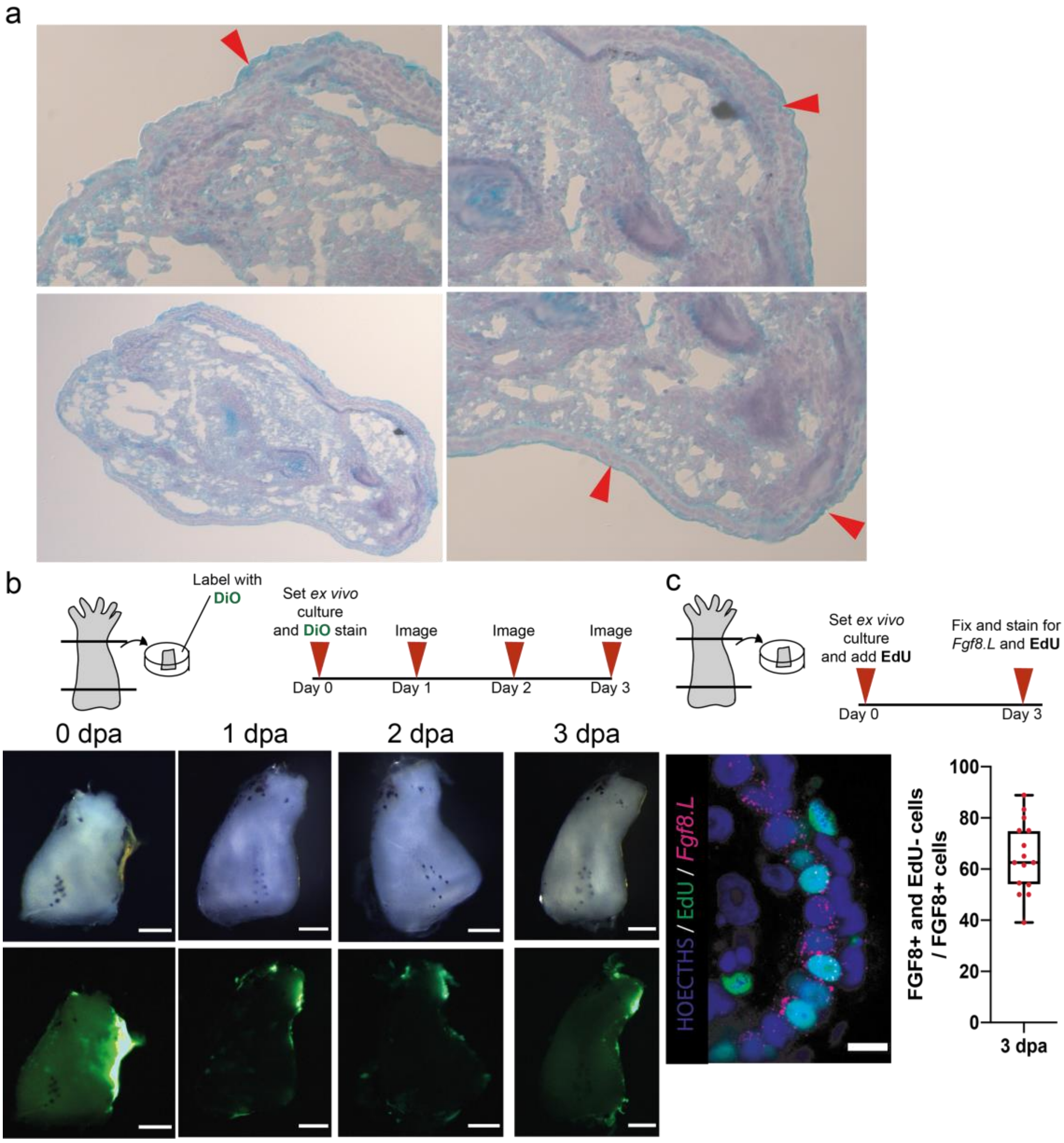

Figure S13: AER cells formation does not require cell division.

(a) 3 dpa regeneration-competent explants are covered with cells morphologically similar to the surrounding basal epidermal cells as evidenced by haematoxylin, eosin, and Alcian blue stain. There are multi-layered or monolayered epidermal cells with cuboidal shape that can be seen not only at the distal site (right-bottom) but also at the lateral sides as well (right-top). A squamous layer can be seen above the basal epidermal cells. **(b)** (Top) Schematic describing DiO based tissue tracing. DiO labelling was performed after *ex vivo* cultures were harvested. Explants were imaged every day until day 3 in culture via stereomicroscope. (Bottom) DiO tracing applied to the sides of explants and traced over time and images were taken in brightfield and green channel. Traced tissues migrated to the distal and proximal amputation planes of explants. Total n = 22 from 2 biological replicates. Scale = 200 µm. **(c)** (Top)

Development • Supplementary information

Schematics describing *ex vivo* culture with EdU treatment. EdU was added to explant media at the beginning of the culture. (Bottom-left) Explants were fixed and stained via HCR for *Fgf8.L*, and EdU after day 3 in culture. (Bottom-middle) Example confocal image of a sectioned sample stained via HCR for *Fgf8.L*, *Lgr5.S*, and EdU, showing that not all *Fgf8.L+/Lgr5.S+* cells are EdU+. Hoechst, Blue; EdU, Green, *Fgf8.L* mRNA, Magenta. Scale = 10 µm. (Bottom-Right) Quantification of EdU positive AER cells proportion to all detected AER cells. Approximately 60% of AER cells are EdU negative. Total n= 15 from 3 biological replicates.

Development • Supplementary information

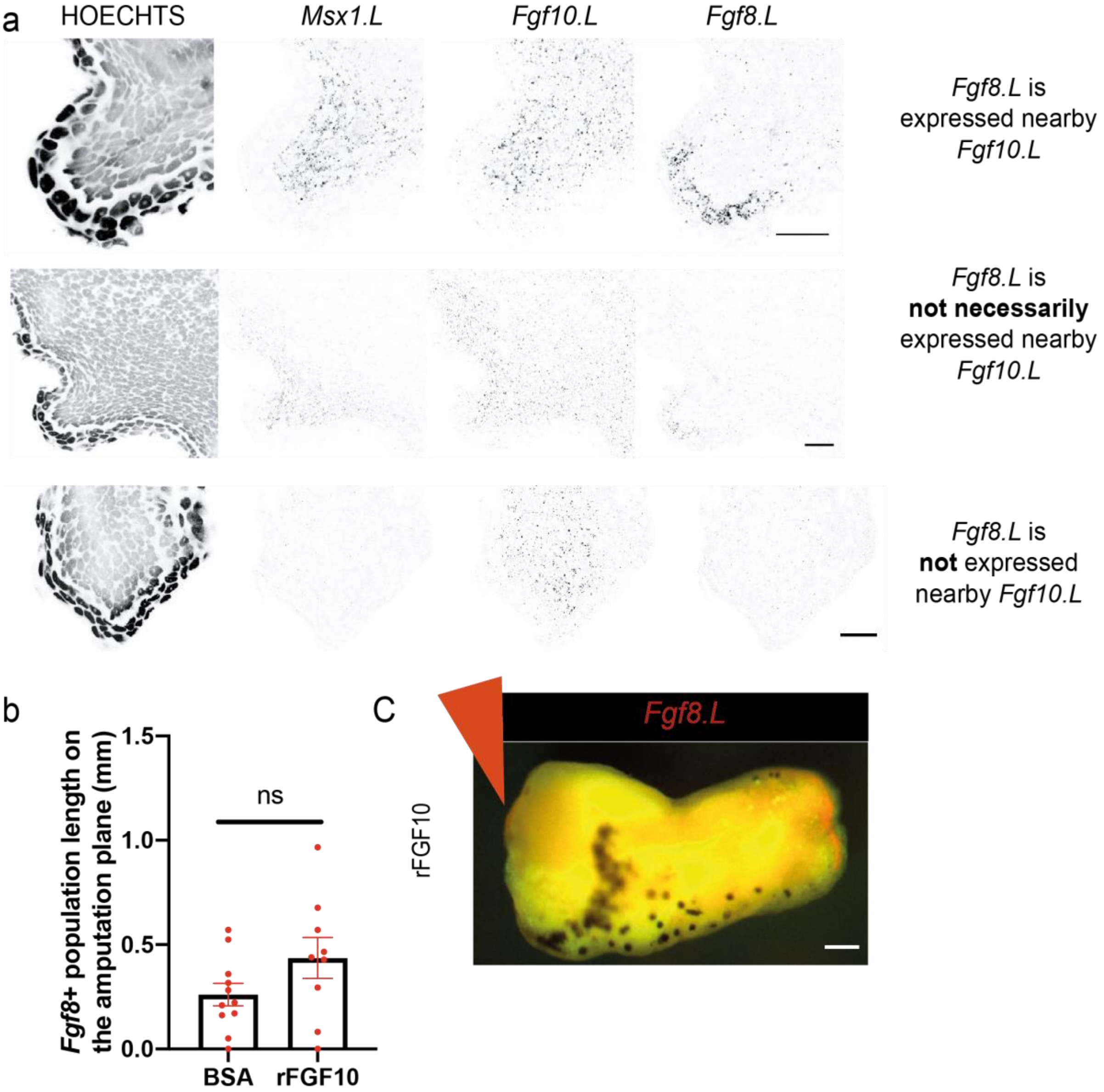

Figure S14: FGF10 is not sufficient to induce *Fgf8* in all epidermis.

(a) Examples of sectioned confocal images of 5 dpa *in vivo* samples from regeneration-competent tadpoles stained for *Msx1.L*, *Fgf10.L* and *Fgf8.L*. Top image series show high levels of *Fgf10.L* and *Msx1.L* in the mesenchyme associated with high levels of *Fgf8.L* in the surrounding epidermis. Middle image series show that not all epidermis in proximity of *Fgf10.L* + mesenchymal cells are expressing *Fgf8.L*. *Msx1.L* + mesenchymal cells are more correlated to *Fgf8.L*+ epidermis than *Fgf10.L* + mesenchymal cells. Bottom, although there is a high level of *Fgf10* expression detected in mesenchyme, no *Fgf8.L* in epidermis or *Msx1.L* in mesenchyme can be seen. Scale, 20 μm. **(b)** Regeneration-competent explants were treated with rFGF10, and 0.1% BSA/PBS was used as control. BSA: total n=11 from 2 biological replicates; FGF10: total n=9 from 2 biological replicates; ns: *P**>0.05. **(c)** Example whole-

Development • Supplementary information

mount stereomicroscope image of rFGF10 treated explants showing a very mild expression of *Fgf8.L* at their proximal sites (n=4/7 from 2 biological replicates). Scale, 200 μm.

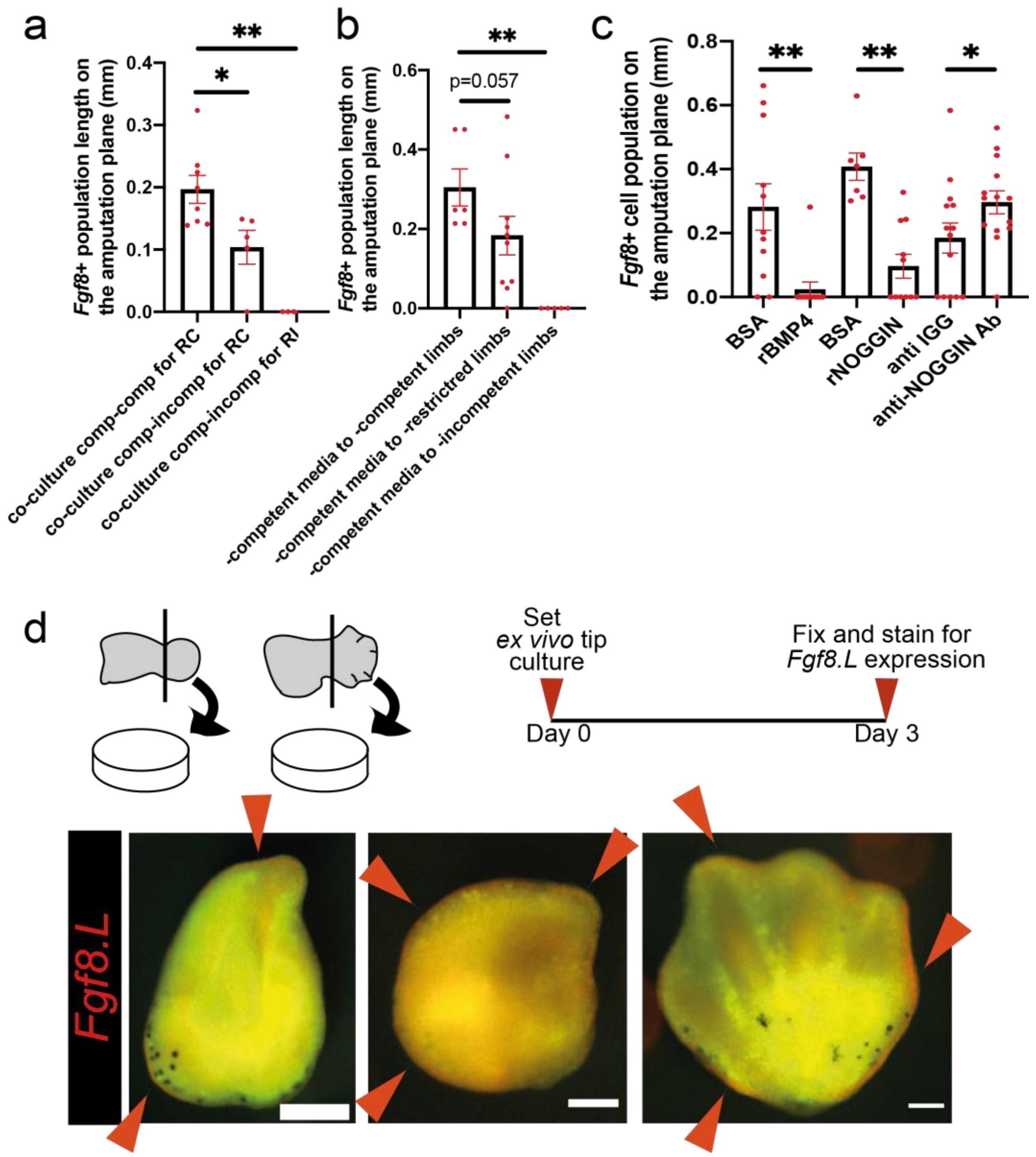

Figure S15: Secreted factors from regeneration-competent explants are not sufficient to induce *Fgf8+* epidermal cells on the amputation plane of regeneration-incompetent explants, and reducing the proportion of chondrogenic lineage populations in explants can induce ectopic *Fgf8+* epidermal cells.

(a) Co-culturing regeneration-competent with -incompetent explants does not enable AER cell formation ability in -incompetent explants. RC: regeneration-competent; RI: regeneration-incompetent. Co-culture of regeneration-competent-competent and assess –competent: total n=8, from 2 biological replicates, Co-culture regeneration-competent-incompetent and assess –competent: n= 5 from 2 biological replicates. Co-culture regeneration-competent-incompetent for -incompetent total n= 6 from 2 biological replicates. *P**< 0.05, and *P***< 0.001. **(b)** Treatment with –competent conditioned media does not enable AER cell formation in -

Development • Supplementary information

incompetent explants. Adding -competent-media to –competent explants: total n=6, from 2 biological replicates. Adding -competent-media to –restricted explants: n= 10, from 3 biological replicates. Adding -competent-media to –incompetent explants: n= 5, from 1 biological replicate. *P***< 0.001. **(c)** Regeneration-competent explants were treated with recombinant BMP4, recombinant NOGGIN, or anti-NOGGIN antibodies. Contralateral limbs were used as controls and treated with vehicle solutions (0.1% BSA, or anti-IGG). Recombinant BMP4 or NOGGIN additions block AER cell formation. Anti-NOGGIN antibody treatment enhances AER cell formation. From left to right 0.1% BSA: total n=11 from 3 biological replicates; rBMP4: total n= 12 from 3 biological replicates; 0.1% BSA: total n=7 from 2 biological replicates; rNOGGIN: total n=11 from 2 biological replicates; anti-IGG antibody: total n=14 from 4 biological replicates; anti-NOGGIN antibody: total n=14 from 4 biological replicates. Each sample group compared to their contralateral group to assess statistical significance. *P**< 0.05, and *P***< 0.001. **(d)** (Top) Schematic describing the protocol for culturing distal limb buds (NF stage ~52) and early autopods (NF Stage ~54). Tip explants were cultured for 3 days in explant media, and assessed for *Fgf8.L* expression. (Bottom) Tip cultures show ectopic *Fgf8.L* expression as assessed by whole-mount stereomicroscope HCR staining for *Fgf8.L*. Red arrows show *Fgf8.L* expression regions. Ectopic AER formation is seen in total 16/18 cases from 2 biological replicates. Red, *Fgf8.L* mRNA. Scale= 100 μm.

Development • Supplementary information

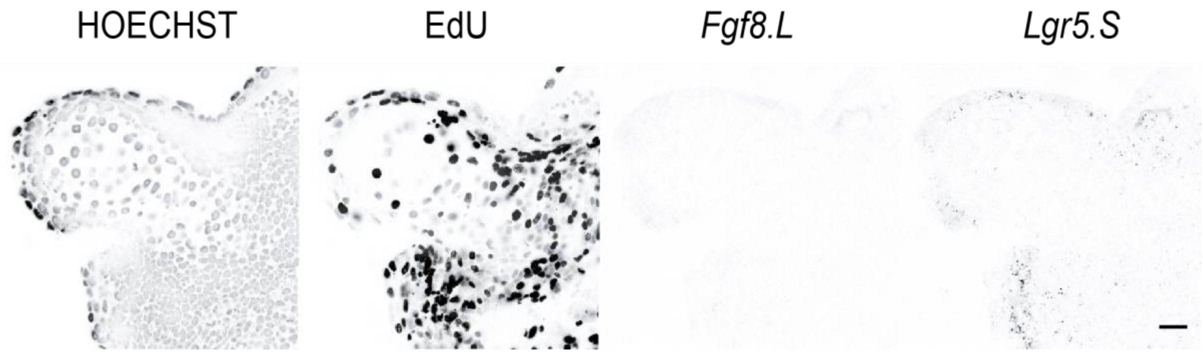

Figure S16: Inhibition of FGF receptor induces chondrogenic populations in the proximal explant.

Example sectioned confocal images of proximal site of 3 dpa explants treated with SU5402 showing sparse circular nucleus indicative of chondrogenic cells as well as lack of *Fgf8.L* in epidermis. Scale bar = 15 µm.

Table S1. Quality control for scRNA-seq

Click here to download Table S1

Table S2. Differentially expressed gene lists for AER cells during development and regeneration, and ligand gene lists used for scGSEA.

Click here to download Table S2

Development • Supplementary information
