# Supplementary material for: Secreted inhibitors drive the loss of regeneration competence in Xenopus limbs
Source: Development. 2021 Jun 9;148(11):dev199158. doi: 10.1242/dev.199158 (PMC8217717; doi:10.1242/dev.199158)
Supplement: Reviewer comments [file develop-148-199158_review_history.pdf]

Secreted inhibitors drive the loss of regeneration competence in *Xenopus* limbs

Can Aztekin, Tom W. Hiscock, John Gurdon, Jerome Jullien, John Marioni and Benjamin David Simons
DOI: 10.1242/dev.199158

Editor: Patrick Tam

Review timeline

Original submission:	7 December 2020
Editorial decision:	6 January 2021
First revision received:	8 April 2021
Editorial decision:	29 April 2021
Second revision received:	4 May 2021
Accepted:	5 May 2021

Original submission

First decision letter

MS ID#: DEVELOP/2020/199158

MS TITLE: Secreted inhibitors drive the loss of regeneration competence in Xenopus limbs

AUTHORS: Benjamin David Simons, Jerome Jullien, John Marioni, John Gurdon, Can Aztekin, and Tom Hiscock

I have now received all the referees' reports on the above manuscript, and have reached a decision. The referees' comments are appended below, or you can access them online: please go to BenchPress and click on the 'Manuscripts with Decisions' queue in the Author Area.

As you will see, the referees express considerable interest in your work, but have some significant criticisms and recommend a substantial revision of your manuscript before we can consider publication. If you are able to revise the manuscript along the lines suggested, which may involve further experiments, I will be happy receive a revised version of the manuscript. Your revised paper will be re-reviewed by one or more of the original referees, and acceptance of your manuscript will depend on your addressing satisfactorily the reviewers' major concerns. Please also note that Development will normally permit only one round of major revision.

We are aware that you may be experiencing disruption to the normal running of your lab that make experimental revisions challenging. If it would be helpful, we encourage you to contact us to discuss your revision in greater detail. Please send us a point-by-point response indicating where you are able to address concerns raised (either experimentally or by changes to the text) and where you will not be able to do so within the normal timeframe of a revision. We will then provide further guidance. Please also note that we are happy to extend revision timeframes as necessary.

Please attend to all of the reviewers' comments and ensure that you clearly highlight all changes made in the revised manuscript. Please avoid using 'Tracked changes' in Word files as these are lost in PDF conversion. I should be grateful if you would also provide a point-by-point response detailing how you have dealt with the points raised by the reviewers in the 'Response to Reviewers' box. If you do not agree with any of their criticisms or suggestions please explain clearly why this is so.

© 2021. Published by The Company of Biologists under the terms of the Creative Commons Attribution License (https://creativecommons.org/licenses/by/4.0/).

Reviewer 1

Advance summary and potential significance to field

The study by Aztekin et al. addresses the loss of complete limb regeneration in the Xenopus laevis froglet. Xenopus have the ability to regenerate hindlimbs up to metamorphosis, and regenerate a spike thereafter. Overall, the study addresses an important unanswered question of why full regeneration is lost during Xenopus development.

Comments for the author

The authors argue that it is likely the lack of the specialized wound epithelium that leads to the regeneration of a spike and refers to this transition to spike regeneration as a loss of regeneration. This argument is a bit ambiguous because Xenopus froglet limbs do regenerate, they just regenerate a spike. Several investigators have argued and provided evidence (Yokoyama et al., 2000; Li et al., 2012; Yakushiji et al., 2007; Carlson, 1982; and others I'm missing) that it is the loss patterning ability in the mesenchyme to pattern the froglet blastema rather than the ability to mount a regenerative response. Froglet limb amputation generates a blastema that is nerve-dependent like urodele limb blastemas (Suzuki et al., 2005) and seems similar to blastemas in frog tadpoles and urodele blastemas. I am basing this on the literature and not experience so I may be missing the observation that most froglet limbs do not regenerate at all. Maybe including the incidence rate of completely failed regeneration versus spike regeneration could strengthen the argument in the introduction.

My argument is not meant to discount the approach because it is clear that a specialized wound epidermis is needed for practically all appendages that regenerate across species, but I feel providing more discussion and references are needed to cover the problem more comprehensively. Also, considering a blastema is generated in the Xenopus froglet, the molecules that are termed inhibitory to regeneration are likely inhibitory to patterning, not blastema formation.

Technically, the study uses the latest in methodologies including scRNAseq, HCR-FISH, and combining in situ hybridization with IHC and histology, while combining these with traditional methodologies like bead implantations. Overall, the methods generated here will be very useful for the research community. As a technical suggestion for later studies, I suggest some use of RI matching medium such as EasyIndex or ethyl cinnamate, which helps overcome autofluorescence and opaqueness when imaging whole mount HCR samples. This is not a request for this manuscript but merely a point of advice.

Another strength of the study is the accessible interactive online platform of the RNAseq data. This will be a great resource for the regeneration community. The manuscript is clear with few grammatical errors.

Lines 72-88: The idea that the AEC of regenerating limbs is analogous to the AER seen in frog, chick, and mouse limbs has changed recently since salamanders do not express FGF ligands fgf8, 9, and 17 in the epithelium of the developing limb (Purushothaman et al., 2019) or AEC of the regenerating limb (Nacu et al., 2016), which has been validated by scRNAseq data (Gerber et al., 2018). In line 83-85, I feel justification for the current study should be modified considering we know the regenerating AEC is necessary, but don't know why it is necessary.

The specialized wound epidermis of the regenerating limb could be phagocytosis of debri, histolysis of underlying tissue, growth support, maintaining the plastic blastemal state of underlying cells, or providing directional growth cues for underlying blastema cells. Considering the froglet limb may have all or some of these roles intact, maybe the AEC loses some key component necessary for limb patterning.

In lines 92-95, it is stated that the progressive loss of limb regeneration in xenopus coincides "with their inability to form a specialized wound epidermis". I think it is important to state that the loss of the WE does not necessarily mean it is the causal reason for losing the ability to regenerate. My reasoning is stated above.

© 2021. Published by The Company of Biologists under the terms of the Creative Commons Attribution License (https://creativecommons.org/licenses/by/4.0/).

Line 128 - The authors state they demonstrated the critical role of inhibitory factors. I think this is overstating. Yes, the results support their hypothesis but they do not demonstrate the necessity and sufficiency of the inhibitory molecules in vivo. One would need to remove these inhibitors to show that regeneration commences in a complete manner in froglets. I think this is a phrasing thing rather than any limitation in the study.

Figure 2E and Line 207-212 – Figure 2E does not show the regeneration incompetent stage 60, which is important to show if the hypothesis is that the AEC gene ligand levels are correlated with regeneration success. Also, I don't see any difference (by eye) between expression levels of the regeneration competent stage 52 5dpa and the regeneration-restricted stage 56 5dpa. Shouldn't the level of these genes decline between these samples?

Line 212 – Is the point that there are AEC cells in regeneration competent and incompetent stages and are approximately equivalent, but their abundance is much lower in regeneration restricted stages? I think this is a fine conclusion, but needs to be highlighted. I'm not sure I see the evidence of "high signaling center potential" correlated with regeneration outcome. There is a lot of data though so maybe this just needs to be highlighted in a more focused manner. For example which genes show the highest differential between AEC genes between regeneration competent and incompetent? What are these gene functions?

Line 220 – do other blastema-specific genes found in urodeles like prrx and cirbp also found like sall4 and kazald1?

Line 258-263 – was there any drug or pathway inhibitor that did not block AEC formation? I think this is as important to show as the ones that did block AEC formation.

Line 267 – "Having established the molecular pathways required for AER cell formation": This is an overstatement of the results from the previous section.

This would all need to be done in vivo to claim this (I'm not stating it should for revision) and what pathways are not necessary? Are these pathways working on the AEC directly or the mesenchyme underneath? The results are fine, but the claim that it is all figured out is too ambitious.

Line 267-285 – yes, the AEC originates from skin cells as stated, but reference others that found this. Also, it is well appreciated in urodeles that most of the proliferation is in cells proximal to the amputation plane early after amputation and the early AEC is generated by migration, not proliferation. These experiments should be cited.

Line 295 – I agree that the fgf10 experiments of Yokoyama et al., 2001 do not explain the formation of the AER on proximal explant tissues, but distal outgrowth is somewhat of a rule for regenerating appendages from insects to amphibians. I would be very surprised to see any functional AEC form on the proximal limb surface. We don't know why, but I don't think this study is addressing this question.

Line 351-353 – Although this idea is interesting, I don't think it is appropriate to speculate on the rule of distal transformation in the results section here.

Line 385-387 – Again, the authors explain the reason for a lack of proximal AEC formation and presume it is due to the abundant antagonist cues. I think this is an overstatement of the data and there is little evidence that this is the case.

It could be due to a multitude of possibilities.

They then follow this with data suggesting that the proximal AEC can be induced with FGF10 and anti-Noggin antibodies. This is a very strong result, but I'm confused from ling 994-999 that two biological replicates were used, but an n = 8.

There needs to be some clarification on the statistical analysis of this because it would be a very important result, but two biological replicates would not be sufficient to make such a claim.

© 2021. Published by The Company of Biologists under the terms of the Creative Commons Attribution License (https://creativecommons.org/licenses/by/4.0/).

Overall, this is an interesting manuscript and I think the data support the correlation with chondrogenesis and the lack of AEC formation. I feel the conclusions are a bit overstated in some places and the last observation of induction of AEC formation on the proximal stump of a limb needs further validation for strong conclusions to be made. Most if not all the points I bring up can be modified without further experiments, which will still lead to a nice contribution to the field. The strong conclusions proposed in the manuscript should be carefully evaluated, which is somewhat raised in lines 467-469, but as a passing point at the end of the manuscript.

Reviewer 2

Advance summary and potential significance to field

Understanding why most vertebrate do not generate functional wound epidermis following limb amputation—and therefore why they might be incapable of mounting a productive regenerative response—is lacking. This paper aims to shed light on the differences between regeneration-competent and regeneration-incompetent epidermis that forms across amputation sites in a single species, Xenopus leavis, that has both regeneration-competent and regeneration-incompetent stages in embryos/developing tadpoles. They are bringing modern tools (single-cell RNAseq and clustering analyses) to an old and longstanding problem, and surely the deposition of this data into the public research domain will be a valuable resource-type contribution. The main issues with this work are conceptual and that some of the main claims have insufficient experimental support. The experiments that are presented are mostly interesting, but there needs to be more of them to make the conclusions they want to make as currently written. The concern is that the authors are interpreting their experimental results both beyond the scope of what they can reasonably support, and they're also downplaying previously published work on this topic. Both of these matters might be fixable if the authors are willing to do so; it will take substantial re-writing (and abandoning some claims), some additional computational analyses (for example, pairwise comparisons between samples), and some more experimentation.

Comments for the author

Specific comments:

• The work published in 2000 and 2001 by Yokoyama et al. should be more thoroughly engaged. In the 2000 paper, they did demonstrate that fgf-10 is expressed in the Xenopus regeneration-competent limb mesenchyme, and they made recombinant limbs with competent mesenchyme and incompetent-staged epidermis and then demonstrated that wound epidermis marker fgf-8 is turned on in these cases. The in situs in this paper are beautiful, and they clearly demonstrate that fgf-10 is expressed in the mesenchyme in stage 52 of developing Xenopus limbs, at the level of the amputations used in this paper as well. Then, in 2001, Yokoyama et al. also showed that FGF-10 protein application is sufficient to promote regeneration in otherwise incompetent-staged Xenopus frog embryos. The second study is mentioned in this manuscript but it is definitely shortchanged. It is possible to thoroughly explain these two works while still making the case that the current manuscript takes the observations in a more granular direction.

• Relatedly, the decision to call these wound epidermis cells "AER" cells throughout the rest of the manuscript needs to be revisited. Removing this nomenclature does not detract from the contribution of new data of the resource variety that this paper will conceivably make to the regeneration community. Keeping it also makes for a confusing read. They simply cannot be called "AER" cells.

• It should be made more clear that when Xenopus are competent to fully regenerate perfect limbs, those original limbs are by no means completely formed/developed. This concept seems to be currently misunderstood in the field, and coverage of the topic like what is submitted here exacerbates the issue. The authors mention this in the introduction and their cartoon in Figure 1 is accurate. Readers should be guided to keep in mind in the text that what is truly regenerative is the developing limb, not the mature limb that has reached either its final morphology or its final differentiation state with respect to the various tissue components.

© 2021. Published by The Company of Biologists under the terms of the Creative Commons Attribution License (https://creativecommons.org/licenses/by/4.0/).

• Concluding the cells that comprise the wound epidermis cells in regeneration-competent Xenopus developing limbs are the same cell types as those in the developing AEC is misleading. This conclusion cannot be supported by sequencing data alone, and making it would also require functional tests (akin to those in the 2000 Yokoyama paper but with molecular follow-up). The sentence in lines 184-187 is a gross overstatement: “Based on their transcriptomic signature, tissue localization, and cellular morphology, these results indicate that the cells composing the AEC do not represent a novel cell type, but rather a re-deployment of developmental AER, albeit with a higher signaling center potential.” Instead, how about:
“Based on our findings, we speculate that the early cells that comprise the wound epidermis in Xenopus limb buds that have been amputated and will go on to regenerate normal limbs are morphologically and transcriptionally similar to the original AER cells that developed there a few days earlier.” The authors do not need to tread into this argument of semantics to make their point, which is basically that they have a larger repertoire of information about these cells, as far as which genes they transcribe or were recently transcribed within them or their parental cells, than previous studies that predate single-cell RNAseq. The notion that AER and AEC are molecular similar is old and supported by lots of studies, but we could all benefit from knowing this more precise information.

• If some of the putative “dedifferentiation” genes identified in salamander studies are already expressed in Xenopus limb buds at the regeneration-competent stages, in the mesenchyme, what does this mean? Can the authors provide an alternative explanation grounded in the idea that these limbs are simply *still* developing, i.e., they are still expressing some of the genes that are characteristic of cells that are not fully differentiated yet? This issue relates to the earlier one I mentioned about making it more clear to readers that this kind of regeneration is predicated on the limb not actually being fully formed/differentiated/developed yet. Still, it is interesting to think about, and framing it as suggested does not mean this work will not be welcomed, digested, debated, and used productively by the research community.

• Relatedly, this sentence is true, but it’s also true that “its extent” tracks with animal age: “Together, we concluded that, upon amputation, a subset of fibroblasts manifest injury-induced mesenchymal plasticity - at least at the transcriptional level - and its extent tracks with AER cell abundance.” It’s a correlation and should be presented as such. Also, there is no true lineage/fate mapping here since it’s just single-cell RNAseq, so we can’t really conclude what they have stated. Even revising to this would convey the more accurate conclusion: “Together, we concluded that, upon amputation, a subset of fibroblasts may be stimulated to express markers known to track with regenerative success in urodeles, and this expression increase at the transcriptional level tracks with AER-cell-like abundance in wound epidermis and inversely tracks with animal maturation.” 舲

• An overt comparison between AER cells (from early limb bud) and the wound epithelium cells proposed to be called “AER” cells should be performed. The only analysis presented to support their claim includes all the other cell types . . .

• The work published in Leigh et al., 2018, is mentioned, but the specific findings about wound epidermis are not. Since this manuscript is centered on regeneration-competent wound epidermis, the authors should relate their findings to that data as well.

• The ex vivo experimentation might actually be the most compelling part of this paper. Noticing that there is not “bidirectional” regeneration when the embryonic limbs removed, explanted, and cultured, and then going a bit further and asking what’s happening on the end that formerly connected to the body is interesting. It’s also noteworthy that the authors did use chemical inhibitors of five different signaling pathways—even if most had already been demonstrated to be involved in limb regeneration—in this ex vivo context. This means that the modulation of these pathways is local to the amputation site, which advances our understanding of the overall process.

• Some of the previous work on where wound epidermis cells come from needs to be dealt with in the manuscript. There are many examples of previous work that supports the notion that they are derived from pre-existing epidermal cells left on the stump, but none of them are referenced or discussed. A good place to start is the seminal work of Elizabeth Hay, for example, Hay and Fischman, 1961, in newts.

© 2021. Published by The Company of Biologists under the terms of the Creative Commons Attribution License (https://creativecommons.org/licenses/by/4.0/).

• Figures 3 and 4 are, in my opinion, the most novel in the paper. One concern, though, about interpreting the data as they have done in 4C with the bar graphs is that we don't really know if the different cell types are differentially amenable to the dissociation, encapsulation, and sequencing protocols or if their data truly reflects a different relative composition of these cell types in the samples from different stages. Certainly tissues at 5dpa, particularly distal ones, are looser and more apt to go through the pipeline successfully.

Some acknowledgement of this limitation would be helpful to others doing these kinds of experiments, and it might also (rightly so) give us pause in interpreting them.

• Perhaps the major shortcoming of the experiments is that while the authors could show that a larger fraction of fgf-8-expressing cells either exists or can be captured and sequenced following treatments that inhibit Noggin, there is no direct regeneration outcome to mirror this finding. The existence of the fgf-8-expressing cells has to suffice as the proxy. To demonstrate that mesenchymal tissues become more activated, and therefore show a greater regenerative response, they should repeat the experiments and follow with a more direct assay. This could be something simple like EdU incorporation in the mesenchymal tissues, but it could also be something else. How long can the cultures be maintained? Is it possible to measure outgrowth? Even with respect to the fgf-8-expressinng epidermal cells, why are there fewer? Can an experiment be performed to distinguish between less recruitment of fgf-8-expressing cells to the wound epithelium versus lower amounts of proliferation of the fgf-8-expressing cells that do reside in the wound epithelium? Do all of the pathways act through the same mechanism?

• Interpretation of the wound epidermal (so-called "AER" cells) as a "signaling center population"— this is a bit dubious to call these such on the basis of them expressing the five ligands called out in Fig. S9. There are so many other signaling molecules known, and certainly many more molecules may act as signaling molecules than are already characterized as such. In this heat map alone, all of the ligands included are substantially expressed in at least one other population that is not "AER." Further, it's unclear which genes these "ligand" molecules correspond to. There are lots of problems here that could be fixed by not drawing such broad conclusions. Also, where's the fgf-10 transcript being expressed among mesenchymal cells?

• Are there any other possible explanations for their ex vivo experiments, in toto, other then their interpretation that chondrogenesis (here, at the previously proximal end) inhibits regeneration? This is an excellent opportunity to elaborate on other possible explanations without staking the claim so fiercely that it's chondrogenesis inhibiting it (and it seems that while the hypothesis is consistent, there are other viable explanations).

• Relatedly, if the authors want to provide more solid evidence that chondrogenesis per se inhibits limb regenerative capacity, then they should devise and execute more convincing experiments. For example, if they remove skeletal elements from the harvested limbs before they culture them, can they demonstrate that these limbs have a longer potential (time frame) for pro-regenerative responses? This experiment could also be attempted in vivo using microsurgery on the animals or possibly by laser ablation. Alternatively, a genetic ablation strategy could be employed.

Reviewer 3

Advance summary and potential significance to field

Understanding of the similarities/differences between the AER (development) and the AEC (regeneration), molecular basis of the formation of the AEC during Xenopus limb regeneration, potential reasons for the loss of regeneration competence in Xenopus limb stages.

Comments for the author

This is a very impressive series of experiments involving single cell RNA sequencing of developmental limb bud stages, regeneration competent regeneration restricted and regeneration

© 2021. Published by The Company of Biologists under the terms of the Creative Commons Attribution License (https://creativecommons.org/licenses/by/4.0/).

incompetent stages in Xenopus. This is not just a characterization of gene expressions by single cell, but it leads to experiments using in vitro limbs which identify the role of the AEC/AER in the transition from regeneration competency to incompetency and the role that chondrogenesis plays in inhibiting the formation of the AEC via the production of Noggin. Although the authors did not get regeneration incompetent stages to regenerate in vivo through noggin/fgf10 manipulation they did get it to happen in vitro in terms of increases in the length and proliferation and AEC production of cultured limbs. Very impressive. This work has great significance for our understanding of the decline of regeneration in Xenopus and possibly for “the potential to unlock the ability to regrow lost limbs in non-regenerative higher vertebrates” (lines 506-507).

The latter suggestion of the authors is contradicted in several places throughout the text where we are rightly cautioned against extrapolating across species. There are two extrapolations which stand out to me in reading this work: one, Urodele limb buds do not have an AER as the text points out (Purushothaman et al), although there is no reason why the AER has to be an identifiable multi-layered structure rather than a single layer of cells with specific gene expressions (as in supplemental figure S7); and two, if Noggin inhibits the AEC why does Noggin application in chick and mouse limb buds promote the AER? I think the latter contradiction should be more rigorously discussed.

I have only one other comment regarding work previously done on the AEC which has been studied in salamanders for more than 70 years and much less so for Xenopus. In the Intro, lines 86-88 ‘Moreover, it is not clear how a specialized wound epidermis forms during limb regeneration, what are the cell types composing the AEC, what is the broad repertoire of genes expressed by it, and why it cannot form in some instances/species’. This is a very Xenopus-centric view because quite a lot is known about the AEC in axolotls/newts. For example it is known that the early migrating epidermal cells which form the AEC do not divide (Hay & Fishman, 1961, Dev Biol. 3, 26-59) - the authors show this here for Xenopus without commenting about this early data from newts, making their findings less “surprising” (line 276); a microarray of the AEC has been done in axolotls (Monaghan et al., 2012)

identifying >1000 genes; many transcription factors are expressed including sp9 which is thought to be crucial for its functioning; the secreted molecules Wnt5a, 5b, and several Fgfs are expressed; several Mmps and extracellular matrix molecules are expressed. This seems to be a “broad repertoire of genes”!

I think at least Hay & Fishman should be referred to because the authors do the very same experiment here (although not with tritiated thymidine admittedly).

There is a ton of other great work here, too much to mention, but I particularly liked the comparison of the AEC with the tail regenerating epithelium form previous work identifying ROCs and also the description of fibroblast changes and their dedifferentiation at different regeneration competent/incompetent stages.

First revision

Author response to reviewers' comments

Reviewer 1 Advance Summary and Potential Significance to Field:
The study by Aztekin et al. addresses the loss of complete limb regeneration in the Xenopus laevis froglet. Xenopus have the ability to regenerate hindlimbs up to metamorphosis, and regenerate a spike thereafter. Overall, the study addresses an important unanswered question of why full regeneration is lost during Xenopus development.

Reviewer 1 Comments for the Author:

© 2021. Published by The Company of Biologists under the terms of the Creative Commons Attribution License (https://creativecommons.org/licenses/by/4.0/).

The authors argue that it is likely the lack of the specialized wound epithelium that leads to the regeneration of a spike and refers to this transition to spike regeneration as a loss of regeneration. This argument is a bit ambiguous because Xenopus froglet limbs do regenerate, they just regenerate a spike. Several investigators have argued and provided evidence (Yokoyama et al., 2000; Li et al., 2012; Yakushiji et al., 2007; Carlson, 1982; and others I'm missing) that it is the loss patterning ability in the mesenchyme to pattern the froglet blastema rather than the ability to mount a regenerative response. Froglet limb amputation generates a blastema that is nerve-dependent like urodele limb blastemas (Suzuki et al., 2005) and seems similar to blastemas in frog tadpoles and urodele blastemas. I am basing this on the literature and not experience so I may be missing the observation that most froglet limbs do not regenerate at all. Maybe including the incidence rate of completely failed regeneration versus spike regeneration could strengthen the argument in the introduction.

We thank the reviewer for this comment, which made us aware that our explanations of tadpole stages was insufficiently clear in the initial manuscript. Our manuscript involves tadpoles (more specifically NF Stage 52-60) and, to our knowledge, these are not considered equivalent to froglets (which are defined specifically as NF Stage 66 and beyond, early post-metamorphosis and sexually immature stages). As the reviewer states, froglets are reported to regenerate a spike in a more homogenous manner. However, this does not seem to be the case for late stage tadpoles. Instead, late stage tadpoles show either simple wound healing/stump formation or spike formation. This was first detected by Dent in his seminal 1962 paper, as well as many different groups including ours subsequently. Dent noted that 5/22 of Stage 55 and 3/18 Stage 57 tadpoles do not show any growth in his 1962 paper. However, these percentages do not seem to be recapitulated in other studies, in line with our own experience. For example, below we refer to some papers exemplifying the situation. In these papers, simple wound healing (rather than spike formation) phenotype has been seen in 58%, 75%, 100% of reported control cases. We do not know of any report where this diversity in phenotypes is systematically assessed or why it happens.

We would also like to note there are more differences between froglets and tadpoles. For example, unlike the situation in froglets, regeneration-competent tadpole limb regeneration was suggested to be a nerve-independent process (Filoni et al. Differentiation, 1990). Moreover, as froglets represent post- metamorphosis stages, their immune system and general metabolism are significantly different to tadpoles, which may again cause differences in amputation outcomes. Finally, some of the post-limbamputation perturbations do not result in the same phenotype for late stage tadpoles and froglets (Lin et al, Dev Cell, 2013).

Nevertheless, we agree that such differences between tadpoles and froglets should be made more clearly to readers and, accordingly, we now cite a detailed review paper (see below Beck et al. 2009 review) in which this situation is described as well as differences in tadpole and froglet regeneration scenarios. Moreover, independent of the differences between froglets and tadpoles, we have discussed the hypothesis that the loss of patterning ability is involved in regeneration incompetency and spike formation in froglets as this may also be relevant in late stage tadpoles.

Revised introduction:
Main text line 114-116:
"Towards metamorphosis, amputations either cause the growth of an unpatterned spike-like cartilaginous structure without joints and muscles, or a simple wound healing response (NF ~58 and beyond, regeneration-incompetent) (Beck et al., 2009; Dent, 1962).."

Additional review cited:
Beck CW, Izpisúa Belmonte JC, Christen B. Beyond early development: Xenopus as an emerging model for the study of regenerative mechanisms. Dev Dyn. 2009 Jun;238(6):1226-48. doi: 10.1002/dvdy.21890. PMID: 19280606.

Examples where limb amputation resulted in either spike formation or simple wound healing:
Zhang M, Chen Y, Xu H, Yang L, Yuan F, Li L, Xu Y, Chen Y, Zhang C, Lin G. Melanocortin Receptor 4 Signaling Regulates Vertebrate Limb Regeneration. Dev Cell. 2018 Aug 20;46(4):397-409.e5. doi: 10.1016/j.devcel.2018.07.021. PMID: 30130530; PMCID: PMC6107305.

Barker DM, Beck CW. Overexpression of the transcription factor Msx1 is insufficient to drive

© 2021. Published by The Company of Biologists under the terms of the Creative Commons Attribution License (https://creativecommons.org/licenses/by/4.0/).

complete regeneration of refractory stage Xenopus laevis hindlimbs. Dev Dyn. 2009 Jun;238(6):1366-78. doi: 10.1002/dvdy.21923. PMID: 19322766.

Kawakami Y, Rodriguez Esteban C, Raya M, Kawakami H, Martí M, Dubova I, Izpisúa Belmonte JC. Wnt/beta-catenin signaling regulates vertebrate limb regeneration. Genes Dev. 2006 Dec 1;20(23):3232-7. doi: 10.1101/gad.1475106. Epub 2006 Nov 17. PMID: 17114576; PMCID: PMC1686599.

Yokoyama H, Ide H, Tamura K. FGF-10 stimulates limb regeneration ability in Xenopus laevis. Dev Biol. 2001 May 1;233(1):72-9. doi: 10.1006/dbio.2001.0180. PMID: 11319858.

My argument is not meant to discount the approach because it is clear that a specialized wound epidermis is needed for practically all appendages that regenerate across species, but I feel providing more discussion and references are needed to cover the problem more comprehensively.

Indeed, this is a very good suggestion. We also agree that the function of the specialised wound epidermis is not clear, especially in *Xenopus*, and it could have many of the features listed by the reviewer (and reiterated in another comment below, rebuttal letter lines 250-255). Among these, we specifically mentioned experiments conducted by Tassava et al. where removal of AEC resulted in no blastema formation, although “dedifferentiation” was observed. We specifically highlighted this finding in our introduction since our injury-induced mesenchymal plasticity phenotype may have some association with the formed AER cells. This is because we see more patterning genes (e.g. Shh) active when there are more AER cells, from which it could be speculated that patterning of the blastema somehow requires AER cells. We stated that further investigation on the interaction between these two topics would be required and mentioned this in line 556-558 (also see below).
Nonetheless, we agree with the concern of the reviewer and have now expand our introduction accordingly.

Revised introduction:
Main text – line 72-90:
“Amphibian limb regeneration relies on a specialized wound epidermis (also known as the apical-epithelial-cap, AEC) that forms on the amputation plane and has been characterized primarily as a tissue in regenerating salamander limbs (Campbell et al., 2011; Campbell and Crews, 2008; Knapp et al., 2013, p. 2013; Monaghan et al., 2012; Pearl et al., 2008; Tsai et al., 2020, 2019). It has been hypothesized that the absence or immature state of this tissue limits the regeneration potential of higher vertebrates, including mammals (Tassava & Olsen, 1982). The AEC has been suggested to impact underlying tissues by: degrading extracellular matrix (Kato et al., 2003; Miyazaki et al., 1996; Yang et al., 1999); secreting growth factors to promote proliferation (Han et al., 2001; Thornton, 1960; Thornton and Thornton, 1965; Tsai et al., 2020); enabling the self-renewal of underlying progenitor and dedifferentiated cells, leading to the formation of a proliferative structure called the blastema (Mescher, 1976; Tassava and Loyd, 1977; Tassava and Mescher, 1975); and providing directionality cues for growth (Ghosh et al., 2008; Thornton, 1960; Thornton and Thornton, 1965). Some marker genes associated with AEC (e.g. Fgf8, Fn1) were specifically seen only in the basal layers of AEC tissue, suggesting there is cellular heterogeneity within the AEC (Christensen and Tassava, 2000; Tsai et al., 2020; Yokoyama et al., 2000). However, it remains largely unclear which cell types within AEC tissue are critical for regeneration, which transcriptional and functional properties are associated with a mature AEC and regeneration, and why the AEC cannot form or mature in some instances/species.

Revised discussion:
Main text line 556-558
“Further work on injury-induced mesenchymal plasticity, its interaction with AER cells, and cross-species comparison on this topic will be required.”

Also, considering a blastema is generated in the Xenopus froglet, the molecules that are termed inhibitory to regeneration are likely inhibitory to patterning, not blastema formation.

This is an interesting idea. Unfortunately, we think our results cannot provide insight into this topic. In our experiments, we specifically focused on very early stages of regeneration (0-3 days

© 2021. Published by The Company of Biologists under the terms of the Creative Commons Attribution License (https://creativecommons.org/licenses/by/4.0/).

post amputation). We did not test whether our identified mechanism effects blastema formation or more late stage phenotypes such as patterning. As we hope the reviewer will agree, the investigation of early stages is much easier than those late stages, and testing whether secreted inhibitors influence later stage events would require substantial new work. Due to these reasons, we think that these would be exciting new directions to explore in future studies. We have mentioned the potential role for blastema patterning in regeneration incompetency in the introduction.

Revised introduction:
Main text- line 125-126:
"Regeneration-incompetency was suggested to result from changes in mesodermal tissue, and may involve defects in patterning of the blastema (Sessions and Bryant, 1988; Yokoyama et al., 2001)."

Technically, the study uses the latest in methodologies including scRNAseq, HCR-FISH, and combining in situ hybridization with IHC and histology, while combining these with traditional methodologies like bead implantations. Overall, the methods generated here will be very useful for the research community. As a technical suggestion for later studies, I suggest some use of RI matching medium such as EasyIndex or ethyl cinnamate, which helps overcome autofluorescence and opaqueness when imaging whole mount HCR samples. This is not a request for this manuscript, but merely a point of advice.

Another strength of the study is the accessible interactive online platform of the RNAseq data. This will be a great resource for the regeneration community. The manuscript is clear with few grammatical errors.

We appreciate the constructive criticisms and suggestions.

Lines 72-88: The idea that the AEC of regenerating limbs is analogous to the AER seen in frog, chick, and mouse limbs has changed recently since salamanders do not express FGF ligands fgf8, 9, and 17 in the epithelium of the developing limb (Purushothaman et al., 2019) or AEC of the regenerating limb (Nacu et al., 2016), which has been validated by scRNAseq data (Gerber et al., 2018).

We thank the reviewer for pointing this out. The expression of epidermal *Fgf8* in salamanders is indeed very interesting as it pertains to our work. Unfortunately, we found it difficult to find a consensus on where and when *Fgf8* is expressed in axolotls, especially within the context of recent single-cell transcriptomics studies.

In summary, Han et al. (Dev Dynamics, 2001) showed that there are *Fgf8* expressing epidermal cells at the basal layers of AEC during axolotl limb regeneration. This description parallels early work of Tassava where he started characterizing molecular properties of AEC tissue (*e.g.* Nace et al 1995) and highlighting the proposal that the basal layer could contain cells critical for regeneration. Very recently, Vincent et al. (Development, 2020) showed *Fgf8* expressing cells in different parts of the AEC during limb regeneration, not just basal layers. Han et al and Purushothaman showed that *Fgf8* is not expressed during limb development in axolotl, which parallels early findings where axolotls were suggested to not have morphologically identified AER during their development, but to have a ridge structure during regeneration (Tank et al. 1977). In the Nacu et al. study, the focus was mostly on an accessory limb model (which is considered as a model for normal limb growth or regeneration by some groups), and *Fgf8 in situs* did not show any labelling. In Gerber et al., 2018, we could not see where *Fgf8* is expressed in the published version of the manuscript (other than mesenchymal cells). Still, even in the Gerber et al study, the cell numbers analysed are very low (with a focus on connective tissue cell types), which may be another reason that it is not reported in the main manuscript. Lastly, we would like to note that there are 4 more axolotl limb regeneration single-cell transcriptomics studies in the literature (3 of them published very recently). Data from Leigh et al (Nat Comm, 2018), and Li et al (Protein Cell, 2020) were not able to detect a separate *Fgf8*+ cell cluster and, in these studies, there is no new cluster emergence upon limb amputations that could be labelled as AEC. Qin et al (Cell Death Differ, 2020) used single-cell transcriptomics and suggested identification of "AEC" as a separate cluster upon limb amputations. However, it was not reported whether this population expresses *Fgf8*. In the Leigh, Li, and Qin papers, the authors comment on basal epidermis clusters. Nevertheless, the marker

© 2021. Published by The Company of Biologists under the terms of the Creative Commons Attribution License (https://creativecommons.org/licenses/by/4.0/).

genes reported for these populations (e.g. Krt12) are not unique to AER cells in our study. The fourth study is from Rodgers et al (Experimental Cell Research, 2020) and was conducted only at post amputation samples. The authors did not report Fgf8+ epidermal cell cluster, and the information about epidermal cell types is very limited. Some ambiguity may be due to using different methods (e.g. different dissociation protocols, staining protocols, sequencing platforms) and some might be due to biological reasons (e.g. using different age animals, using accessory limb model).

Collectively, we think that these studies demonstrate that there is still an evolving understanding of the salamander AEC and its potential similarity (or difference to) a development AER program. We certainly agree that this data represents an important perspective to understanding the AER-AEC analogy, and have edited the main text accordingly.

Revised introduction:
Main text line 92-102:
"Due to their requirement for proximal-distal outgrowth as well as the similarity in Fgf8 expression patterns, the AEC in regenerating limbs was suggested to be analogous to the apical-ectodermal- ridge (AER), a tissue that has been well-studied during mouse and chicken limb development (Beck et al., 2009). However, current results suggest that limb regeneration-competent salamanders lack a developmental AER (Purushothaman et al., 2019). Moreover, recent findings (including single-cell transcriptomic data) have provided conflicting results on epidermal Fgf8 expression during axolotl limb regeneration (Gerber et al., 2018; Han et al., 2001; Leigh et al., 2018; Li et al., 2020; Nacu et al., 2016; Qin et al., 2020; Rodgers et al., 2020; Vincent et al., 2020). Therefore, it is unclear if cells within AEC tissue use a novel transcriptional programme for regeneration, or whether they re-deploy a transcriptional programme associated with developmental AER."

In line 83-85, I feel justification for the current study should be modified considering we know the regenerating AEC is necessary, but don't know why it is necessary. The specialized wound epidermis of the regenerating limb could be phagocytosis of debri, histolysis of underlying tissue, growth support, maintaining the plastic blastemal state of underlying cells, or providing directional growth cues for underlying blastema cells. Considering the froglet limb may have all or some of these roles intact, maybe the AEC loses some key component necessary for limb patterning.

We discussed this point above (rebuttal letter – lines 123-134) and edited our manuscript according to the comment from the reviewer. We are grateful to the reviewer for emphasizing this issue.

In lines 92-95, it is stated that the progressive loss of limb regeneration in Xenopus coincides "with their inability to form a specialized wound epidermis". I think it is important to state that the loss of the WE does not necessarily mean it is the causal reason for losing the ability to regenerate. My reasoning is stated above.

Whilst this correlation has been generally accepted in the literature, we agree that it should be also mentioned that the causal link to regeneration is not clear in *Xenopus* and we have edited our text accordingly.

Main text line 87-90
"However, it remains largely unclear which cell types within AEC tissue are critical for regeneration, which transcriptional and functional properties are associated with a mature AEC and regeneration, and why the AEC cannot form or mature in some instances/species."

Main text line 106-110:
"Moreover, tadpoles lose their limb regeneration ability progressively during development, coinciding with their inability to form a specialized wound epidermis, although the mechanisms of regeneration incompetence and their connection to the specialized wound epidermis remain incompletely understood (Christen & Slack, 1997; Dent, 1962)."

Line 128 – The authors state they demonstrated the critical role of inhibitory factors. I think this is

© 2021. Published by The Company of Biologists under the terms of the Creative Commons Attribution License (https://creativecommons.org/licenses/by/4.0/).

overstating. Yes, the results support their hypothesis, but they do not demonstrate the necessity and sufficiency of the inhibitory molecules in vivo. One would need to remove these inhibitors to show that regeneration commences in a complete manner in froglets. I think this is a phrasing thing rather than any limitation in the study.

We thank the reviewer for this comment, and certainly share the aspiration to investigate the necessity and sufficiency of the inhibitory molecules *in vivo*. However, as we discussed in our manuscript, this is a difficult hypothesis to test as we cannot remove/transfer all secreted factors together *in vivo*. However, by focusing on a single inhibitor (*Noggin*), we performed two experiments to investigate the *in vivo* function of the secreted inhibitors and we present them below.

First, in an effort to reduce the amount of a secreted inhibitor, we implanted anti-NOGGIN beads on the amputation plane of regeneration-restricted/incompetent tadpoles *in vivo* and tested the regeneration-outcome. When we conducted this experiment, we observed a slight improvement in the number of formed digits/digit-like structures, further supporting the hypothesis that secreted inhibitors limit regeneration-competency in late stage tadpoles. We see that this perturbation has a mild effect compared to our FGF10 bead rescue experiment in regeneration-restricted/incompetent tadpoles (**Figure 5A**). We do not know if this mild effect is due to technical problems with the perturbation or due to biological reasons. These could be: (1) diffusivity of antibodies from beads is limited and causing a milder phenotype; (2) beads are repelled within a day, and hence there would be a transient Noggin removal – meanwhile according to our other findings and model, blocking FGF10 would have a longer effect compared to this transient Noggin removal; and (3) Noggin is one of the secreted inhibitors but it is likely others are playing a role as well (including Chrdl1 and Frzb that are still highly expressed in chondrogenic lineage) **(Figure 4D)**. Hence, anti-NOGGIN beads may have only removed a small part of the overall inhibition. Meanwhile, implanting FGF10 would block secretion of the other secreted inhibitors as well since it blocks the cellular source of them (chondrogenesis). We now include this experiment in results and discuss potential caveats in the discussion, as indicated below.

Revised in results:
Main text line 378-383
"Based on these observations, we then explored whether anti-NOGGIN application would improve the in vivo amputation response. Indeed, when beads loaded with anti-NOGGIN antibodies were implanted on the amputation plane of regeneration-restricted/incompetent tadpoles, we saw a mild improvement in the regenerative response ***(Figure 5A)****, highlighting that secreted inhibitors are influencing the regeneration-outcome in vivo."*

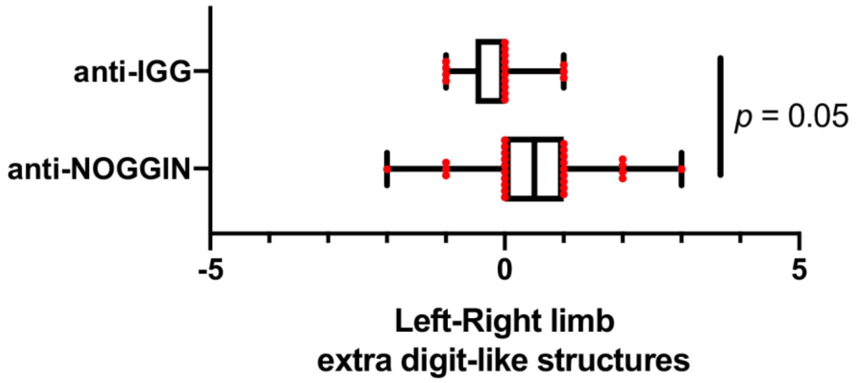

Anti-NOGGIN antibody application to distal amputations improve regeneration in –restricted and –incompetent tadpoles. –Restricted and –incompetent tadpole right and left hindlimbs were amputated and beads containing anti-IGG antibody or anti-NOGGIN antibody were placed on the right hindlimbs. Formed digits and digit-like structures were quantified in the right and left hindlimbs and the difference calculated. Anti-IGG antibody total n=17 from 3 biological replicates; Anti-NOGGIN antibody total n=28 from 4 biological replicates.

Second, based on our *ex vivo* experiments (Fig 5F), we expected NOGGIN would operate downstream of the effect of FGF10. However, we did not test this hypothesis *in vivo* at the time of

© 2021. Published by The Company of Biologists under the terms of the Creative Commons Attribution License (https://creativecommons.org/licenses/by/4.0/).

our initial submission. As reported by Yokoyama et al, we demonstrated that FGF10 can restore regenerative abilities in regeneration-restricted and –incompetent tadpoles. In the current study, we also conducted co-application of FGF10 + NOGGIN beads to the amputation plane of regeneration- restricted and –incompetent tadpoles *in vivo*. We found that co-application of FGF10 + NOGGIN decreases the positive effect of FGF10 only beads, corroborating our *ex vivo* findings (Fig 5E) and suggesting that the secreted inhibitors may be the "bottle-neck" event driving regeneration-outcome. We now mention this experiment in our results, and hope that the reviewer considers these experiments to further strengthen the findings in this study.

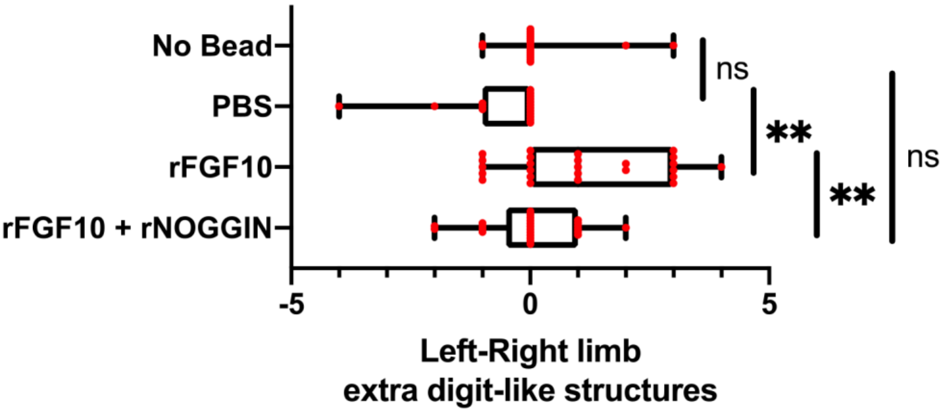

Recombinant FGF10 application to distal amputations restore regeneration in –restricted and –incompetent tadpoles. -Restricted and –incompetent tadpole right and left hindlimbs were amputated and beads containing 0.1% BSA/PBS or recombinant FGF10 or recombinant FGF10 and NOGGIN were placed on the right hindlimbs. Formed digits and digit-like structures were quantified in the right and left hindlimbs and the difference calculated. Empty total n=19 from 2 biological replicates; 0.1%/PBS bead total n=17 from 5 biological replicates; recombinant FGF10 bead total n=25 from 5 biological replicates; recombinant FGF10 and NOGGIN bead total n=25 from 4 biological replicates. ns = not significant, *P***< 0.001.

Revised in results:
Main text: 436-442
"To further test this finding in vivo, we asked if the positive effect of FGF10 in regeneration-incompetent tadpoles could be abrogated by simultaneous NOGGIN addition. For this, we inserted beads co-loaded with FGF10 and NOGGIN to the amputation plane of regeneration-restricted/incompetent tadpoles and found this significantly decreased the positive effect of FGF10- only beads (Figure 5G). These results further emphasise that FGF10 operates upstream of NOGGIN, and hence that secreted inhibitors play a dominant role in determining regeneration-outcome."

Revised in discussion:
Main text lines 505-514
"We then tested our model in vivo and found that indeed removal of secreted inhibitors (e.g. NOGGIN), or blocking the source of secreted inhibitors (chondrogenic progression via FGF10 application) could improve the regeneration-outcome in regeneration defective stages. Moreover, we demonstrated that NOGGIN cancels the positive effect of FGF10 application, further highlighting the downstream role played by the secreted inhibitors. Overall, these results align with previous transplantation experiments showing that mesoderm from regeneration-incompetent limbs is inhibitory to regeneration (Sessions and Bryant, 1988; Yokoyama et al., 2001, 2000). However, in contrast to previous interpretations, we suggest that an important contributor to this phenomenon is the enrichment of chondrogenic cell abundance within the mesoderm tissue which express inhibitory secreted factors."

Revised discussion:
Main text line: 516-523

© 2021. Published by The Company of Biologists under the terms of the Creative Commons Attribution License (https://creativecommons.org/licenses/by/4.0/).

"We further showed that by manipulating NOGGIN and FGF10 levels we could improve amputation-outcomes in regeneration-restricted/incompetent tadpoles. We see that anti-NOGGIN beads have a mild effect compared to FGF10 beads (Figure 5A and 5G), which may suggest that there are other inhibitors secreted from the chondrogenic lineage (e.g. Chrdl1, Frzb) that must also be eliminated to ensure robust regeneration. However, the mild effect of anti-NOGGIN may also be due to technical problems with the perturbation (e.g. limited duration and/or diffusivity of antibody delivery), and that a more complete inhibition of NOGGIN function would further improve the amputation-outcome."

Revised methods:
Main text line: 711-719

"Affi-gel blue gel beads (Bio-rad, ***1537301****) were incubated with the following proteins overnight at 4 degrees: 2-3 µg Rabbit-IGG isotype control antibody (ab37415); 2-3 µg anti-NOGGIN antibody (ab16054); 0.1% BSA; 1 µg recombinant human FGF10 (R&D, 345-FG) in 1-2 µl 0.1% BSA; 1-1.5 µg recombinant human FGF10 (R&D, 345-FG) and 2.5-4 µg recombinant human NOGGIN (R&D, 6057-NG) in 3-4 µl 0.1% BSA. Tadpoles were anaesthetized with 0.002% MS222, placed on a wet towel, and both right and left hindlimbs were amputated from ankle level in either -restricted or -incompetent tadpoles. 3-4 beads were placed on the amputation plane of the right hindlimb. Left hindlimbs served as an internal control for the experiments."*

Figure 2E and Line 207-212 - Figure 2E does not show the regeneration incompetent stage 60, which is important to show if the hypothesis is that the AEC gene ligand levels are correlated with regeneration success.

We apologise for the confusion here. The striking feature of regeneration-incompetent Stage 60 tadpoles is their complete absence of AER cells (Fig 2C&D). In Figure 2E we plot the variation in ligand expression levels *within* the AER cell cluster, looking for individual gene expression differences. However, as AER cells are not found in regeneration-incompetent 5 dpa samples (Fig 2C&D), we could not include this stage in the plot.

As the reviewer suggests, however, "AER genes" will be conspicuously reduced in Stage 60 tadpoles. To demonstrate this to the reviewer, we have plotted the same gene expressions for all TP63+ epidermal cells (see below) clearly showing a reduction in critical AER ligands. However, we think that adding this data may not be necessary and might confuse the readers. We would be happy to include this data if the reviewer thinks it appropriate.

© 2021. Published by The Company of Biologists under the terms of the Creative Commons Attribution License (https://creativecommons.org/licenses/by/4.0/).

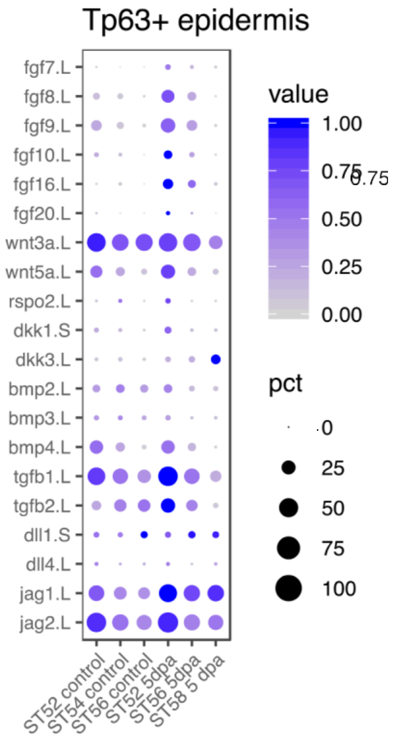

Dot plot showing expression of selected ligands for TP63+ epidermal cells during development and at 5 dpa in regeneration-competent, -restricted, and –incompetent samples. Dot color indicates mean expression; dot size represents the percentage of cells with non-zero expression.

Also, I don’t see any difference (by eye) between expression levels of the regeneration competent stage 52 5dpa and the regeneration-restricted stage 56 5dpa. Shouldn’t the level of these genes decline between these samples?

We thank the reviewer for this comment. We now plotted our previous Fig 2E with a bar plot in included it in our new Fig S6 (see below). This version makes it clearer that some ligands decline in Stage 56 5 dpa AER cells compared to Stage 52 5 dpa, albeit this decrease is very mild for most ligands.

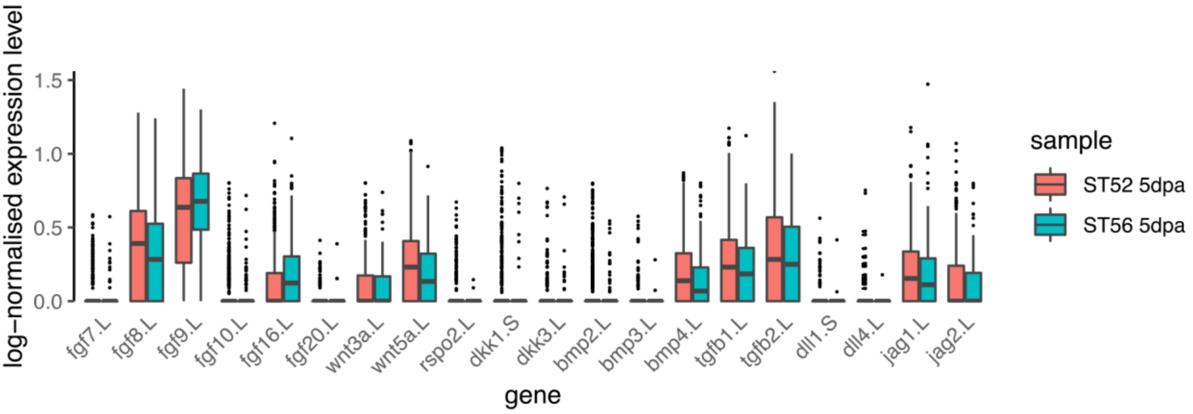

Log10-normalized gene expression visualised using boxplots to compare expression levels between Stage 52 5 dpa and Stage 56 5 dpa AER cells.

Revised result:
Main text lines 223-236
“Using our atlas, we found that, at 5 dpa, tadpole epidermis contained abundant AER cells in regeneration- competent tadpoles and a limited number of AER cells in regeneration-restricted tadpoles, while AER cells were largely absent from regeneration-incompetent tadpoles (Figures

© 2021. Published by The Company of Biologists under the terms of the Creative Commons Attribution License (https://creativecommons.org/licenses/by/4.0/).

2B-D). The signaling center properties of AER cells were reflected in the many diverse ligands they express, which can influence proliferation and cell fate decisions (Figures 2E, S6). Although Fgf8 was always expressed in AER cells, the relative expression of Fgf8 and other ligands varied among conditions (Figure 2E, S6, Supplemental Table 2), emphasizing that the detection of Fgf8 alone does not discriminate the signaling center potency of AER cells. Indeed, in addition to the changes in AER cell abundance, we also detected differentially expressed genes between AER cells from regeneration-competent and –restricted tadpoles (Figure S6B). These differences suggest that AER cells in regeneration-competent 5 dpa samples may be more "mature" compared to regeneration-restricted ones, although further work on the functional role of these genes is required."

Revised methods:
Main text lines 706-708
"Differentially expressed genes were identified using the findMarkers function (using default parameters, and comparing cells from different conditions); results were then visualized as volcano plots."

Line 212 – Is the point that there are AEC cells in regeneration competent and incompetent stages and are approximately equivalent, but their abundance is much lower in regeneration restricted stages? I think this is a fine conclusion, but needs to be highlighted. I'm not sure I see the evidence of "high signaling center potential" correlated with regeneration outcome. There is a lot of data though so maybe this just needs to be highlighted in a more focused manner. For example, which genes show the highest differential between AEC genes between regeneration competent and incompetent? What are these gene functions?

We are grateful for this comment. We observe that both the abundance, and the expression of ligands change between regeneration-competent and regeneration-restricted tadpoles. We have now revised our manuscript to highlight these two changes, as the reviewer suggested.

Additionally, we now conduct differential expression analysis between Stage 52 5 dpa and Stage 56 5 dpa AER cells, and include a volcano plot and table summarizing the results. We detect both quantitative, and some qualitative gene expression differences between AER cells in these samples. Among these genes, we recognize the majority of differentially expressed genes were previously not associated with regeneration (see below volcano plot) and it would be exciting to investigate them in the future. As our study does not focus on these target genes, we include this analysis in Figure S6, DE analysis in supplemental table 2 for readers to access, and mention them in indicated lines below.

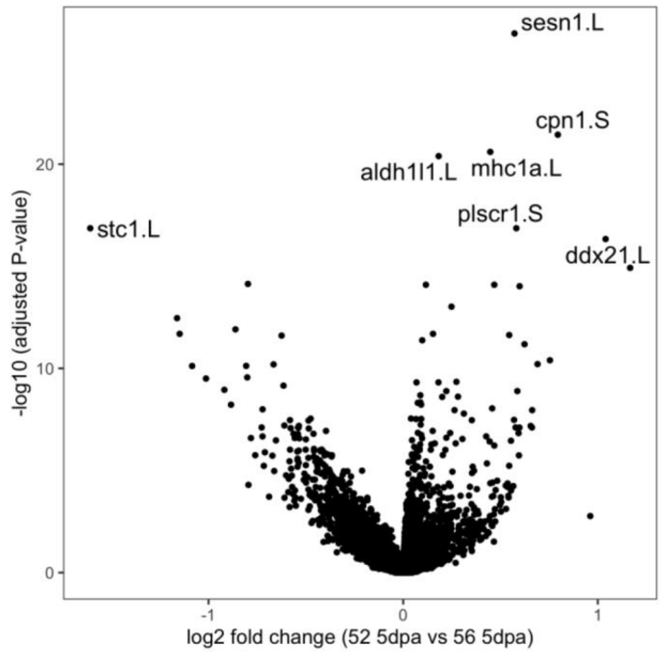

© 2021. Published by The Company of Biologists under the terms of the Creative Commons Attribution License (https://creativecommons.org/licenses/by/4.0/).

*“Differentially expressed genes are detected in AER cells between pairs of conditions and visualised using volcano plots. In **(a)** ….in **(b)**, Stage 56 5dpa is compared to Stage 52 5dpa samples. “*

Revised result:
Main text: 225-237
“The signaling center properties of AER cells were reflected in the many diverse ligands they express, which can influence proliferation and cell fate decisions (Figures 2E, S6). Although Fgf8 was always expressed in AER cells, the relative expression of Fgf8 and other ligands varied among conditions (Figure 2E, S6, Supplemental Table 2), emphasizing that the detection of Fgf8 alone does not discriminate the signaling center potency of AER cells. Indeed, in addition to the changes in AER cell abundance, we also detected differentially expressed genes between AER cells from regeneration- competent and –restricted tadpoles (Figure S6B). These differences suggest that AER cells in regeneration-competent 5 dpa samples may be more “mature” compared to regeneration-restricted ones, although further work on the functional role of these genes is required. Overall, while the signaling center potency of AER cells appeared variable, the redeployment of this developmental cell type with a high signaling center potential had a strong correlation with regeneration-outcome.”

Line 220 – do other blastema-specific genes found in urodeles like prrx and cirbp also found like sall4 and kazald1?

In our manuscript, we see Sall4 upregulated in the Fibroblast 1 cluster upon amputation, irrespective of regeneration-competency. This result suggests that Sall4 could be an injury-response gene.
Meanwhile, Kazald1 expression in Fibroblast 1 is very similar before and after limb amputations. We tried to distinguish these two phenotypes in our Fig S10B.

We detect both Cirbp and Prrx1 transcripts in some cells and found their expression to be maintained before and after amputations for the Fibroblast 1 cluster. We had already included Prrx1 data in the original submission in Fig S10B, and have now updated this figure to include Cirbp, as suggested by the reviewer. We see that Cirbp acts in a similar manner for the Fibroblast 1 cluster (see the plot below).

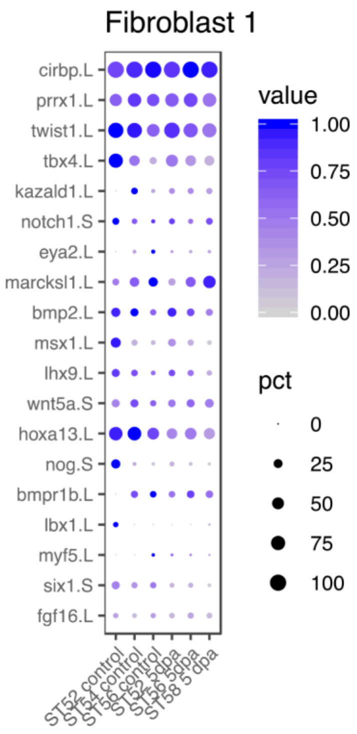

© 2021. Published by The Company of Biologists under the terms of the Creative Commons Attribution License (https://creativecommons.org/licenses/by/4.0/).

We are aware that previous studies show Cirbp and Prrx1 levels to increase in blastema tissue upon amputations. However, these findings were derived from colorimetric ISH, bulk-RNA-seq, or qPCR in blastema *tissue* in which it is not possible to determine transcript per cell information, but rather the cell numbers that express Prrx1 or relative average Prrx1 transcript amount in the tissue. We also see Fibroblast 1 population abundance change upon amputations in Fig 4C, which might explain the previous results. Based on our results, we speculate that previous results were not indicative of a change in expression level, but rather a change in population abundance between conditions. Another possibility is that our results do not align with previous work due to species specific gene expression differences.

Line 258-263 – was there any drug or pathway inhibitor that did not block AEC formation? I think this is as important to show as the ones that did block AEC formation.
We tested more small molecules than we reported in the manuscript and, indeed, some showed negligible effects on AER cell formation, whereas others even showed positive effects. A more comprehensive exploration / screening of the impact of small molecule inhibitors on AER cells is beyond the scope of the current study and is currently being investigated. We provide below an example of a drug treatment where we do not see an effect on AER cell formation; although we have not further characterized this phenotype nor performed a dose-response analysis, and so do not wish to include this in the manuscript at this preliminary stage of characterization.

We have removed unpublished data provided for the referees in confidence.

In our manuscript, to validate our novel assay with explants and HCR based quantifications, we decided to target WNT, BMP, and FGF pathways as these were previously shown to be required for *Xenopus* limb regeneration (although the effect on AEC was not tested for FGF perturbation in *Xenopus*). Then, we tested NOTCH and TGF-B as potential new targets that were not investigated in the context of *Xenopus* limb regeneration.

Line 267 – “Having established the molecular pathways required for AER cell formation”: This is an overstatement of the results from the previous section. This would all need to be done in vivo to claim this (I’m not stating it should for revision) and what pathways are not necessary? Are these pathways working on the AEC directly or the mesenchyme underneath? The results are fine, but the claim that it is all figured out is too ambitious.

Absolutely! We are sorry if our language came across as too strong and we are grateful to the reviewer for pointing this out. Although, we see that downstream elements of these five pathways are active in AER cells (e.g. Msx1/2- BMP, Lgr5 – WNT, Dusp –FGF, Hes1, NOTCH), potential new candidates should be tested *in vivo* and these experiments do not determine if the effect is direct or indirect.
We now deleted the phrasing “Having established the molecular pathways required for AER cell formation” and replaced it with “*Next,…*” (see below). Then, to make it more clear, we have now revised our text as below.

Revised result:
Main text line 294
“Next, we asked how AER cells form on the amputation plane.”

Revised result
Main text line: 288-290
“Overall, we concluded that AER cell formation requires the activity of multiple major signaling pathways, although further work is required to determine what roles these pathways play and whether they directly or indirectly regulate AER cell formation.”

© 2021. Published by The Company of Biologists under the terms of the Creative Commons Attribution License (https://creativecommons.org/licenses/by/4.0/).

Line 267-285 – yes, the AEC originates from skin cells as stated, but reference others that found this. Also, it is well appreciated in urodeles that most of the proliferation is in cells proximal to the amputation plane early after amputation and the early AEC is generated by migration, not proliferation. These experiments should be cited.

All reviewers commented on this topic, and we thank them for raising this issue. We now cite suggested key paper from Hay and Fischman, 1961 and a review paper on wound epidermis by Campbell and Crews, 2008, citing other relevant studies.

As Reviewer 3 also notes, the properties of *Xenopus* AEC are less well known than their counterparts in salamanders. Additionally, molecular characterization of *Xenopus* limb explants was not done before. Critically, in the aforementioned studies, the specialised wound epidermis was investigated as a *tissue*, without characterizing its specific cell types. Here, we specifically tested if AER cells within specialised wound epidermis *tissue* are formed after cell division. For this, we stained against *Fgf8* mRNA which corresponds to a specific population within the basal layer of AEC tissue, and check for EdU co-stained cells. On another note, we note that our scRNA-Seq data also suggests that AER cells are not very proliferative (Fig S2D). Moreover, not just in salamander AEC, but also mouse AER has been suggested to not proliferate much (Storer et al, Cell, 2013). We have edited the text accordingly.

Revised texts:
Main text line: 294-318
"Next, we asked how AER cells form on the amputation plane. It has been suggested that salamander AEC tissue forms by migration of epidermal cells to the amputation plane, and does not require cell proliferation (Campbell and Crews, 2008; Hay and Fischman, 1961). Moreover, the mouse AER was previously suggested to be a largely mitotically inactive tissue (Storer et al., 2013). However, it is not known whether similar mechanisms apply to AER cells within the specialised wound epidermis, and also to what extent they are seen in Xenopus. Therefore, we first traced skin tissue located on the edge of explants, and found that they contributed to the covering of both the distal and proximal sites (Figure S13B). As the amputation planes are covered by skin tissue from the surrounding area, we reasoned that AER cells are likely to have originated from skin cells. As amputation eliminates the majority, if not all, of AER cells in the limb, we hypothesized that AER cells are derived from remaining skin stem cells. If AER cells are induced through proliferation and differentiation following amputation, all AER cells should be the product of cell division. To test this hypothesis, we assayed the level of EdU incorporation (labelling newly synthesized DNA, hence divided cells) in newly-formed AER cells, using Fgf8 positivity to specifically identify AER cells within the AEC tissue. We found that only ~40% of AER cells (distal epidermal Fgf8+) were EdU positive at 3 dpa (Figure S13C), suggesting that most AER cells are induced independently of cell division following amputation. These results parallel our transcriptomics-based cell-cycle assessment in which AER cells display low levels of proliferation (Figure S2D). Using the transcriptomics data, we identified a stepwise activation of Lgr5.S (a WNT target gene) followed by Fgf8.L expression as a possible gene-expression trajectory that could allow basal epidermal cells to convert directly to AER cells without cell division (Figure 3D). Consistent with such a process, when visualized in vivo, we found that Fgf8+/Lgr5+ AER cells were flanked by Lgr5+ cells in the basal epidermis on the amputation plane or in the developing limb (Figures 3E and S7A-B). Overall, these results support the hypothesis that basal epidermal cells can acquire AER cell identity without cell division. "

Line 295 – I agree that the fgf10 experiments of Yokoyama et al., 2001 do not explain the formation of the AER on proximal explant tissues, but distal outgrowth is somewhat of a rule for regenerating appendages from insects to amphibians. I would be very surprised to see any functional AEC form on the proximal limb surface. We don't know why, but I don't think this study is addressing this question.

Line 351-353 – Although this idea is interesting, I don't think it is appropriate to speculate on the rule of distal transformation in the results section here.

We agree with the reviewer, and note that it was not our intention to relate our findings to the rule of distal transformation or distal outgrowth phenomena!

© 2021. Published by The Company of Biologists under the terms of the Creative Commons Attribution License (https://creativecommons.org/licenses/by/4.0/).

Here, we used proximal and distal sides of explants for different purposes as these sides have different compositions (as the proximal side contains more mature chondrogenic tissues compared

to the distal side). Based on this, we tested our model in different experiments throughout our study, all of which focused on AER cell formation rather than the rule of distal transformation. Nonetheless, we agree that we have not fully characterized why the proximal explant epidermis fails to form AER and have amended this sentence accordingly. In addition, we have added a new paragraph in the discussion that covers the rule of distal transformation in more detail.

Revised results
Main text line 391-393
“Moreover, the inability of the proximal explant epidermis to form AER might be explained, at least in part, by the abundance of chondrogenic cells at the proximal site (Figure 3A-B).”

Revised discussion:
Main text line 525-532
“It is well established that a salamander blastema will only form in a location distal to the amputation plane, a phenomenon termed as the rule of distal transformation (Butler, 1955; Nacu and Tanaka, 2011; Stocum, 1981). In our explants, we also detect that only distal sites started to form a blastema (Figure 3A), aligning with the rule of distal transformation. Interestingly, by manipulating NOGGIN and FGF10, we also could observe AER cell formation at the proximal sites of explants (Figure 5F).
However, it remains unclear if these proximal AER cells can enable the formation of a proximal blastema. Further work is required to investigate the relation between the rule of distal transformation and AER cells.”

Line 385-387 – Again, the authors explain the reason for a lack of proximal AEC formation and presume it is due to the abundant antagonist cues. I think this is an overstatement of the data and there is little evidence that this is the case. It could be due to a multitude of possibilities. They then follow this with data suggesting that the proximal AEC can be induced with FGF10 and anti-Noggin antibodies. This is a very strong result, but I’m confused from ling 994-999 that two biological replicates were used, but an n = 8. There needs to be some clarification on the statistical analysis of this because it would be a very important result, but two biological replicates would not be sufficient to make such a claim.

Firstly, we agree that there might be many contributing factors that explain the inability of AEC formation in proximal explant sites, and have changed our text accordingly as below.

Main text line 427-429:
“Nonetheless, FGF10 treatment was not sufficient to induce strong Fgf8 expression at the proximal site of explants ***(Figure S14C)****, which may be, at least in part, due to abundant antagonist cues. “*

Regarding the replicates for this experiment, we hope to clarify this here as we think there might be some confusion. The experiment about proximal AER cells is reported in initial submission version lines 1000-1002, not line 994-999. The proximal site phenotype we see did not happen in all explants, but only in 5/9 samples from two independent experiments. We do not know why only some explants showed this phenotype. Importantly, in none of the other Fgf8 staining on explants used throughout this study (which easily surpasses 100 individual tadpole limbs from many different biological backgrounds) did we see AER cells forming at the proximal site. We computed a Fisher Exact Test to evaluate the statistical significance of these observations; this yielded $p < 0.0001$and was recorded in the caption of Figure 5f. Hence, we are confident that anti Noggin and FGF10 co-application has an effect which can result in Fgf8 expressing cells appearing at the proximal site of regeneration- competent explants.

© 2021. Published by The Company of Biologists under the terms of the Creative Commons Attribution License (https://creativecommons.org/licenses/by/4.0/).

Regarding the statistical approaches in the paper, in our initial submission, we wrote details of what we consider as biological replicates in our “Materials and Methods” section. We further edited this section and hope that it is made more clear:

Revised methods line 882-885
“Sample sizes were not pre-determined in any experimental setup. In this work, biological replicates refer to samples obtained from multiple animal batches and to experiments carried out on different days. In all experiments, the number of independent tadpole limbs assayed is recorded and denoted by n in the text and figure legends.”

Overall, this is an interesting manuscript and I think the data support the correlation with chondrogenesis and the lack of AEC formation. I feel the conclusions are a bit overstated in some places and the last observation of induction of AEC formation on the proximal stump of a limb needs further validation for strong conclusions to be made. Most if not all the points I bring up can be modified without further experiments, which will still lead to a nice contribution to the field. The strong conclusions proposed in the manuscript should be carefully evaluated, which is somewhat raised in lines 467-469, but as a passing point at the end of the manuscript.

We thank the reviewer very much for their constructive criticism and we think their suggested changes significantly improve the manuscript.

Reviewer 2 Advance Summary and Potential Significance to Field:
Understanding why most vertebrate do not generate functional wound epidermis following limb amputation—and therefore why they might be incapable of mounting a productive regenerative response—is lacking. This paper aims to shed light on the differences between regeneration-competent and regeneration-incompetent epidermis that forms across amputation sites in a single species, Xenopus leavis, that has both regeneration-competent and regeneration-incompetent stages in embryos/developing tadpoles. They are bringing modern tools (single-cell RNAseq and clustering analyses) to an old and longstanding problem, and surely the deposition of this data into the public research domain will be a valuable resource-type contribution. The main issues with this work are conceptual and that some of the main claims have insufficient experimental support. The experiments that are presented are mostly interesting, but there needs to be more of them to make the conclusions they want to make as currently written. The concern is that the authors are interpreting their experimental results both beyond the scope of what they can reasonably support, and they’re also downplaying previously published work on this topic. Both of these matters might be fixable if the authors are willing to do so; it will take substantial re-writing (and abandoning some claims), some additional computational analyses (for example, pairwise comparisons between samples), and some more experimentation.

Reviewer 2 Comments for the Author:
Specific comments:

- The work published in 2000 and 2001 by Yokoyama et al. should be more thoroughly engaged. In the 2000 paper, they did demonstrate that fgf-10 is expressed in the Xenopus regeneration-competent limb mesenchyme, and they made recombinant limbs with competent mesenchyme and incompetent-staged epidermis and then demonstrated that wound epidermis marker fgf-8 is turned on in these cases. The in situs in this paper are beautiful, and they clearly demonstrate that fgf-10 is expressed in the mesenchyme in stage 52 of developing Xenopus limbs, at the level of the amputations used in this paper as well. Then, in 2001, Yokoyama et al. also showed that FGF-10 protein application is sufficient to promote regeneration in otherwise incompetent-staged Xenopus frog embryos. The second study is mentioned in this manuscript, but it is definitely shortchanged. It is possible to thoroughly explain these two works while still making the case that the current manuscript takes the observations in a more granular direction.

We regret that our citations to referred papers were found to be insufficient. We mentioned both studies in our manuscript, although we referred to Yokoyoma 2001 more often as it builds on the findings from Yokoyama 2000.

© 2021. Published by The Company of Biologists under the terms of the Creative Commons Attribution License (https://creativecommons.org/licenses/by/4.0/).

We have now revised our manuscript and discussed these studies in more detail (please see below for our changes). However, we would note that, in these studies, the authors conducted **tissue** transplantation without addressing **cellular heterogeneity** within the transplanted tissues and without using many molecular tools/markers, which bring **considerable limitations** for us to interpret and comment on those results. For example, if the grafted tissue in Yokoyama et al would have more Fibroblast 1 cluster cells versus Fibroblast 2 cluster cells (definitions based on our analysis), we may need to re-interpret the findings accordingly. However, we only have tissue information, not cell type/state information.

The general conclusion in those studies were “intrinsic” properties of mesoderm *tissue* causes regeneration-incompetency. Although the authors comment in some parts of their paper that intrinsic properties of individual cells change, as described above, their assessment is at the tissue-level not cellular-level. Our **results align** with those from Yokoyama et al. in that the mesoderm *tissue* changes its cellular composition during development (more mature chondrogenic lineage increase in proportion during limb development), which results in more secreted inhibitors impairing the ability of the limb to regenerate. Some may consider this change in cellular composition of the mesenchymal tissue as the tissue is becoming intrinsically incompetent. Our **conclusions differ** with those outlined in Yokoyama et al, as our results very strongly suggest that secreted inhibitors from regeneration-incompetent tadpoles impair the AER-cell formation and influence the regeneration-outcome, even though the intrinsic properties of the tissue favor regeneration. Particularly, our co-culture and conditioned media experiments, as well as new *in vivo* experiments (explained below), highlight the fact that, even if the intrinsic properties favour regeneration, secreted inhibitors act as the dominant process impacting the regeneration-outcome.

We are grateful to the reviewer for bringing these concerns to our attention and have now expanded our discussion accordingly.

Revised discussion:
Main text line: 466-487
“Recent research has focused on the intrinsic properties of mesodermal tissue and its ability to induce specialized wound epidermis (via Fgf10 expression), supported by the observation that transplantation of mesoderm tissue from regeneration-incompetent limbs prevents regeneration in -competent Xenopus limbs (Sessions and Bryant, 1988; Yokoyama et al., 2000). However, this approach is not able to discriminate whether cells are intrinsically incompetent or whether secreted factors cause this effect, as both would be transferred at the same time (as well as the numerous caveats associated with tissue transplantation). Moreover, this hypothesis does not explain why FGF10 is insufficient to induce AER cells across the entire epidermis, nor why regeneration outcomes are significantly correlated with the extent of ossification at the amputation plane (Dent, 1962; Nye and Cameron, 2005; Wolfe et al., 2000). Inspired by our scRNA-seq data, we sought to determine whether other secreted factors could also be contributing to regeneration incompetency. To our knowledge, there is no practical way to obtain secreted factors from -incompetent tadpoles and transfer them to -competent animals in vivo. Therefore, we established ex vivo cultures that faithfully recapitulated in vivo regeneration to test this critical hypothesis. We identified AER cells in the ex vivo limbs using spatially resolved and quantitative measurement of epidermal Fgf8 via HCR (Choi et al., 2018). Our scRNA-seq data demonstrated that high epithelial Fgf8 expression is a unique late-stage marker in the establishment of AER cell identity (Figure 3D), and therefore Fgf8 positivity in our experimental setup corresponds with high precision to the AER cell type. By using our explant systems and conducting co-culture and conditioned media experiments, both of which would be inaccessible in vivo, we found that secreted inhibitory factors in regeneration-incompetent tadpoles negatively impact AER cell formation.“

Revised discussion:
Main text line: 505-514
“We then tested our model in vivo and found that indeed removal of secreted inhibitors (e.g. NOGGIN), or blocking the source of secreted inhibitors (chondrogenic progression via FGF10 application) could improve the regeneration-outcome in regeneration defective stages. Moreover, we demonstrated that NOGGIN cancels the positive effect of FGF10 application, further highlighting the downstream role played by the secreted inhibitors. Overall, these results align with previous transplantation experiments showing that mesoderm from regeneration-incompetent

© 2021. Published by The Company of Biologists under the terms of the Creative Commons Attribution License (https://creativecommons.org/licenses/by/4.0/).

limbs is inhibitory to regeneration (Sessions and Bryant, 1988; Yokoyama et al., 2001, 2000). However, in contrast to previous interpretations, we suggest that an important contributor to this phenomenon is the enrichment of chondrogenic cell abundance within the mesoderm tissue which express inhibitory secreted factors.“

- Relatedly, the decision to call these wound epidermis cells "AER" cells throughout the rest of the manuscript needs to be revisited. Removing this nomenclature does not detract from the contribution of new data of the resource variety that this paper will conceivably make to the regeneration community. Keeping it also makes for a confusing read. They simply cannot be called "AER" cells.

The comment on lines 832-847 is closely linked to this one; we respond to both comments there, at line 849 onwards.

- It should be made more clear that when Xenopus are competent to fully regenerate perfect limbs, those original limbs are by no means completely formed/developed. This concept seems to be currently misunderstood in the field, and coverage of the topic like what is submitted here exacerbates the issue. The authors mention this in the introduction and their cartoon in Figure 1 is accurate.
Readers should be guided to keep in mind in the text that what is truly regenerative is the developing limb, not the mature limb that has reached either its final morphology or its final differentiation state with respect to the various tissue components.

We fully agree with the reviewer that it is critical to highlight what exactly is regenerating. Due to this, as the reviewer also points out, we tried our best to reflect this in our text and schematics. To alleviate the reviewer concern, we now further stress in our text that our findings are tadpole specific by adding the sentence below:

Revised discussion:
Main text line: 600-602
"Finally, in this work we have identified a cellular mechanism governing regeneration-incompetency in in developing tadpoles, although it remains unclear whether similar principles apply in adult frogs with a more definite limb."

We recognize that the reviewer brings up related points in other comments, and try to respond to them more fully here. Firstly, we note that it is hard to determine when a limb is "mature" or "completely formed/developed" and also that different groups have different opinions on this. When we look at *Xenopus* limbs, using both histology-based results (Dent, 1962) and scRNA-Seq approaches, NF Stage 56 (regeneration-restricted) (Fig 3C) limbs have differentiated cell types with almost no progenitor cells. Moreover, these limbs are patterned and are in their secondary growth – something that is rather distinct from the early phases of limb development. Further, we are aware that even with axolotl, which is the most commonly used animal for limb regeneration studies, different groups use 4-5 cm in length neotenic axolotl, and some groups use 20 cm length neotenic axolotl. Some of these studies would clearly be conducted with animals that are in their secondary growth for their limb development. Hence, some may consider NF Stage 56 tadpole limbs are similar to neotenic axolotl limbs. We are also aware that there are studies not using neotenic axolotl, as they are deemed as a juvenile state by some groups, but use post-metamorphosis animals or just newts. For these reasons, we are concerned that stressing this difference even more could create confusion, particularly given the difficulty in defining "what is completely formed/developed" and the lack of consensus on this topic.

- Concluding the cells that comprise the wound epidermis cells in regeneration-competent Xenopus developing limbs are the same cell types as those in the developing AEC is misleading. This conclusion cannot be supported by sequencing data alone, and making it would also require functional tests (akin to those in the 2000 Yokoyama paper but with molecular follow-up). The sentence in lines 184-187 is a gross overstatement: "Based on their transcriptomic signature, tissue localization, and cellular morphology, these results indicate that the cells composing the AEC do not represent a novel cell type, but rather a re-deployment of developmental AER, albeit with a higher signaling center potential." Instead, how about: "Based on our findings, we speculate that the early cells that comprise the wound epidermis in Xenopus limb buds that have been amputated

© 2021. Published by The Company of Biologists under the terms of the Creative Commons Attribution License (https://creativecommons.org/licenses/by/4.0/).

and will go on to regenerate normal limbs are morphologically and transcriptionally similar to the original AER cells that developed there a few days earlier." The authors do not need to tread into this argument of semantics to make their point, which is basically that they have a larger repertoire of information about these cells, as far as which genes they transcribe or were recently transcribed within them or their parental cells, than previous studies that predate single-cell RNAseq. The notion that AER and AEC are molecular similar is old and supported by lots of studies, but we could all benefit from knowing this more precise information.

We thank the reviewer for these comments. We appreciate that it is difficult to define and name cell types, and we spent a lot of time in interpreting the data and in choosing the "AER cells" name. After much consideration, we still think that this is the most appropriate name for this cell cluster in our data, and have made substantial edits to the text to explain our choice. We outline our arguments below, describe our edits at the end, and hope that this clarifies this issue.

Here we used an unbiased scRNA-Seq approach to identify cell types in *Xenopus*. Unlike regenerative salamaders and non-regenerative mammals, *Xenopus* has the advantage that it can form both AER and AEC tissues and so these can be compared within a single species. When analyzing the scRNA-seq atlas and defining cell types using an unbiased clustering algorithm, we found a single highly expressing *Fgf8*+ epidermal cluster – i.e. a set of cells with highly similar transcriptional programmes - that overlaps with the definitions of AER and AEC tissues, suggesting to us that the cell types comprising the AER and AEC may be rather similar. By point of contrast, we did not see two completely separate *Fgf8*+ epidermal clusters with one specifically forming upon amputation, which would indicate the AEC being a completely novel cell state.
To further assess the degree of transcriptional similarity of these cells between development and regeneration, we have now performed more comprehensive comparisons between these two samples. First, we conducted an unbiased differential expression analysis between developmental and regeneration samples (see new Figure S6, and supplementary table 2). Whilst we did find some gene expression differences – and highlight these in the revised manuscript – many key regeneration genes were expressed in both samples. For example, *Fgf8* and *Fn1* were found to be highly upregulated at 5 dpa compared to the developmental sample; however developmental AER cells still express both *Fn1* and *Fgf8*, at a lower level. To explore this more systematically, we re-plotted marker gene dotplots from our original submission (Fig S2), now segregating developmental and regeneration samples. We observe that AER marker genes are expressed during both development and regeneration, albeit with some genes showing varying levels of expression (e.g. *Fgf8*). Overall, our new analyses do not provide evidence to reject the hypothesis that these cells are (transcriptionally) very similar.

It is on the basis of this transcriptional similarity (together with the similar single-cell morphology and tissue location) that we decided to name the highly expressing *Fgf8*+ epidermal cluster defined by our scRNA-seq data: "AER cells". Based on extensive discussions with correspondents following the bioRxiv submission, and the remarks made by other reviewers, we would maintain that, for non- specialist readers, this naming – properly reasoned – is the most appropriate for this cell type.

We considered the following alternative names but consider these to be inferior to "AER cells":
- **"AEC cells"** is misleading, since the AEC appears *after* the AER, and the AEC in other species/regeneration paradigms is rather distinct (e.g. zebrafish fin AEC does not express Fgf8).
- **"AER-like cells"** leads to confusion since we have developmental AER cells that we would have to refer to as "AER-like"
- **A novel/separate name (e.g. *Fgf8*+ epidermal cells):** we do not think that this is suitable, since our results strongly suggest that cells defining the AEC are not a novel cell state, but are similar to the developmental AER.

We considered using "AER cells" for development and "AER-like cells" for regeneration throughout the text. However, we found this to be confusing for the reader, both for specialist and non-specialists, since we would be referring to a single scRNA-seq cluster and population with the same morphology and location using two different names. Instead, in our revised text, we have highlighted the distinction between developmental AER versus regenerative AEC by referring to "AER cells" with their sample/condition (e.g., ST52 AER cells correspond to the developmental AER).

© 2021. Published by The Company of Biologists under the terms of the Creative Commons Attribution License (https://creativecommons.org/licenses/by/4.0/).

The reviewer suggests transplantation experiments to test the functional equivalence of AER cells during development and regeneration. Although we very much welcome this suggestion, transplanting cell types is not as straightforward as transplanting tissues. *AER cells* within AER or AEC tissues are mostly found as basal monolayer cells and, currently, it is not possible to isolate AER cells while keeping this spatial organization intact. Moreover, it would be extremely challenging to graft them into another animal without creating problems with inflammation and/or injury. Finally, we do not wish to imply that AER cells during development and regeneration are functionally identical, but rather are highly similar and belong to the same cluster in our scRNA-seq atlas.

We are aware that there is no strict definition for a cell type. Therefore, it is critical for us to state on which basis we consider cells defining AEC and AER to be the same/similar. By detailing and describing how we have defined “AER cells”, we do not consider our naming to be misleading. Instead, we hope that by providing our criteria for naming specific populations within these tissues as “AER cells”, we leave the reader to decide whether they would consider them to be similar or different.

We are grateful to the reviewer for raising this concern and hope that our edits strengthen the manuscript. In particular, we made three major changes:

1- Based on the reviewer comment, we now included more statements about differences between tissue-level and cellular level characterisations related to AEC and AER tissues. We also included statements related to the usage of *Fgf8* to detect AEC in salamanders (as also indicated by the Reviewer 1).

In introduction:
Main text lines 72-106
“Amphibian limb regeneration relies on a specialized wound epidermis (also known as the apical-epithelial-cap, AEC) that forms on the amputation plane and has been characterized primarily as a tissue in regenerating salamander limbs (Campbell et al., 2011; Campbell and Crews, 2008; Knapp et al., 2013, p. 2013; Monaghan et al., 2012; Pearl et al., 2008; Tsai et al., 2020, 2019). It has been hypothesized that the absence or immature state of this tissue limits the regeneration potential of higher vertebrates, including mammals (Tassava & Olsen, 1982). The AEC has been suggested to impact underlying tissues by: degrading extracellular matrix (Kato et al., 2003; Miyazaki et al., 1996; Yang et al., 1999); secreting growth factors to promote proliferation (Han et al., 2001; Thornton, 1960; Thornton and Thornton, 1965; Tsai et al., 2020); enabling the self-renewal of underlying progenitor and dedifferentiated cells, leading to the formation of a proliferative structure called the blastema (Mescher, 1976; Tassava and Loyd, 1977; Tassava and Mescher, 1975); and providing directionality cues for growth (Ghosh et al., 2008; Thornton, 1960; Thornton and Thornton, 1965). Some marker genes associated with AEC (e.g. Fgf8, Fn1) were specifically seen only in the basal layers of AEC tissue, suggesting there is cellular heterogeneity within the AEC (Christensen and Tassava, 2000; Tsai et al., 2020; Yokoyama et al., 2000). However, it remains largely unclear which cell types within AEC tissue are critical for regeneration, which transcriptional and functional properties are associated with a mature AEC and regeneration, and why the AEC cannot form or maturate in some instances/species.
Due to their requirement for proximal-distal outgrowth as well as the similarity in Fgf8 expression patterns, the AEC in regenerating limbs was suggested to be analogous to the apical-ectodermal-ridge (AER), a tissue that has been well-studied during mouse and chicken limb development (Beck et al., 2009). However, current results suggest that limb regeneration-competent salamanders lack a developmental AER (Purushothaman et al., 2019). Moreover, recent findings (including single-cell transcriptomic data) have provided conflicting results on epidermal Fgf8 expression during axolotl limb regeneration (Gerber et al., 2018; Han et al., 2001; Leigh et al., 2018; Li et al., 2020; Nacu et al., 2016; Qin et al., 2020; Rodgers et al., 2020; Vincent et al., 2020). Therefore, it is unclear if cells within AEC tissue use a novel transcriptional programme for regeneration, or whether they re-deploy a transcriptional programme associated with developmental AER.

Xenopus laevis is the only commonly used model organism that develops their limbs in a similar manner to amniotes, has a detectable AER, and shows limb regeneration ability (Purushothaman et al., 2019)”

© 2021. Published by The Company of Biologists under the terms of the Creative Commons Attribution License (https://creativecommons.org/licenses/by/4.0/).

In results:
Main text lines 190-209
"Using our single-cell atlas, we compared the transcriptional profiles of cells that belonged to the AER (defined as Fgf8 expressing epidermal cells during limb development) and the AEC (Fgf8 expressing epidermal cells in 5 dpa samples). Whilst we did see some quantitative expression differences between cells related to AEC and AER tissues (Figure S5, S6A, Supplementary Table 2), they expressed many genes in common and showed a high degree of transcriptional similarity (Figures 2A-B, S5). Consistent with this, cells related to these tissues were aggregated within a single Fgf8+ epidermal cluster (Figures 2B-C). Additionally, both during development and 5 days post-amputation, Fgf8+ epidermal cells were mostly detected as a monolayer of polarized cuboidal basal cells (Figure S7), though multilayers were seen to form in some instances (Figure S8). This suggests that AEC and AER tissues are not homogenous in their cellular composition, and that it is only the basal cells that express the key Fgf8+ transcriptional program. Overall, based on their transcriptomic signature, tissue localization, and cellular morphology, the Fgf8+ cells that compose the AEC and AER tissues are very similar. We find that the AEC tissue does not require a novel cell state, but rather a re-deployment of the transcriptional program associated with developmental AER, albeit with a higher signaling center potential (Figure 2E, S6C-D). Due to their high-degree of similarity and common expression of developmental AER genes, we named all cells from the identified Fgf8+ epidermal cluster as AER cells, and referred to specific samples to distinguish between cells from the regeneration-associated AEC and the developmental AER."

In discussion:
Main text lines 446- 454
"Limb regeneration and its requirement for a mature specialized wound epidermis (the AEC) is a well- established phenomenon with extensive tissue and gene level investigations. Here, moving beyond tissue-level descriptions, we reveal cell types and transcriptional states mediating Xenopus limb regeneration and AEC tissue by using single-cell transcriptomics and ex vivo regenerating limb cultures. Transcriptome and morphological assessment indicate that the transcriptional programmes and cells defining AEC and AER tissues are largely the same, differing only in the magnitude of their signaling center potential. Hence, AEC does not seem to involve a novel transcriptional programme specific for regeneration-competent species, but rather the activation of a programme that is highly reminiscent of developmental AER, at least in Xenopus."

2- Following the suggestions from the reviewer, we now include our two new analyses (as described above) comparing AER cells during development and regeneration. Results from these analyses can be found in Fig S5 and Fig S6.

Revised Fig S5:

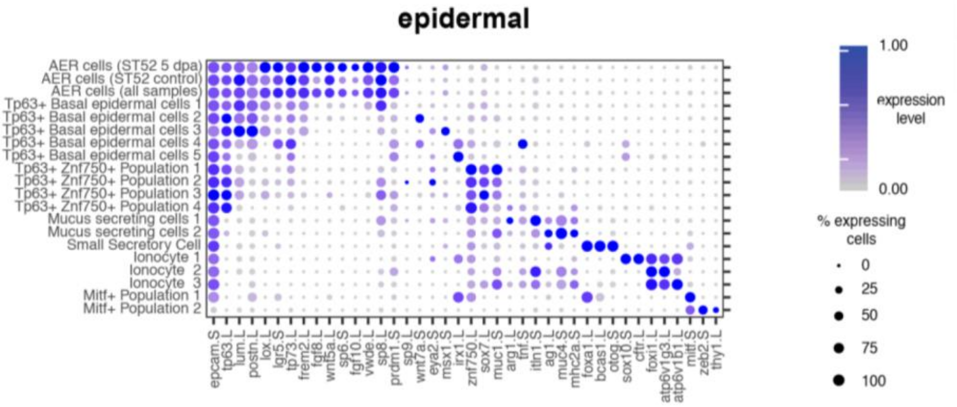

© 2021. Published by The Company of Biologists under the terms of the Creative Commons Attribution License (https://creativecommons.org/licenses/by/4.0/).

Revised new Fig S6

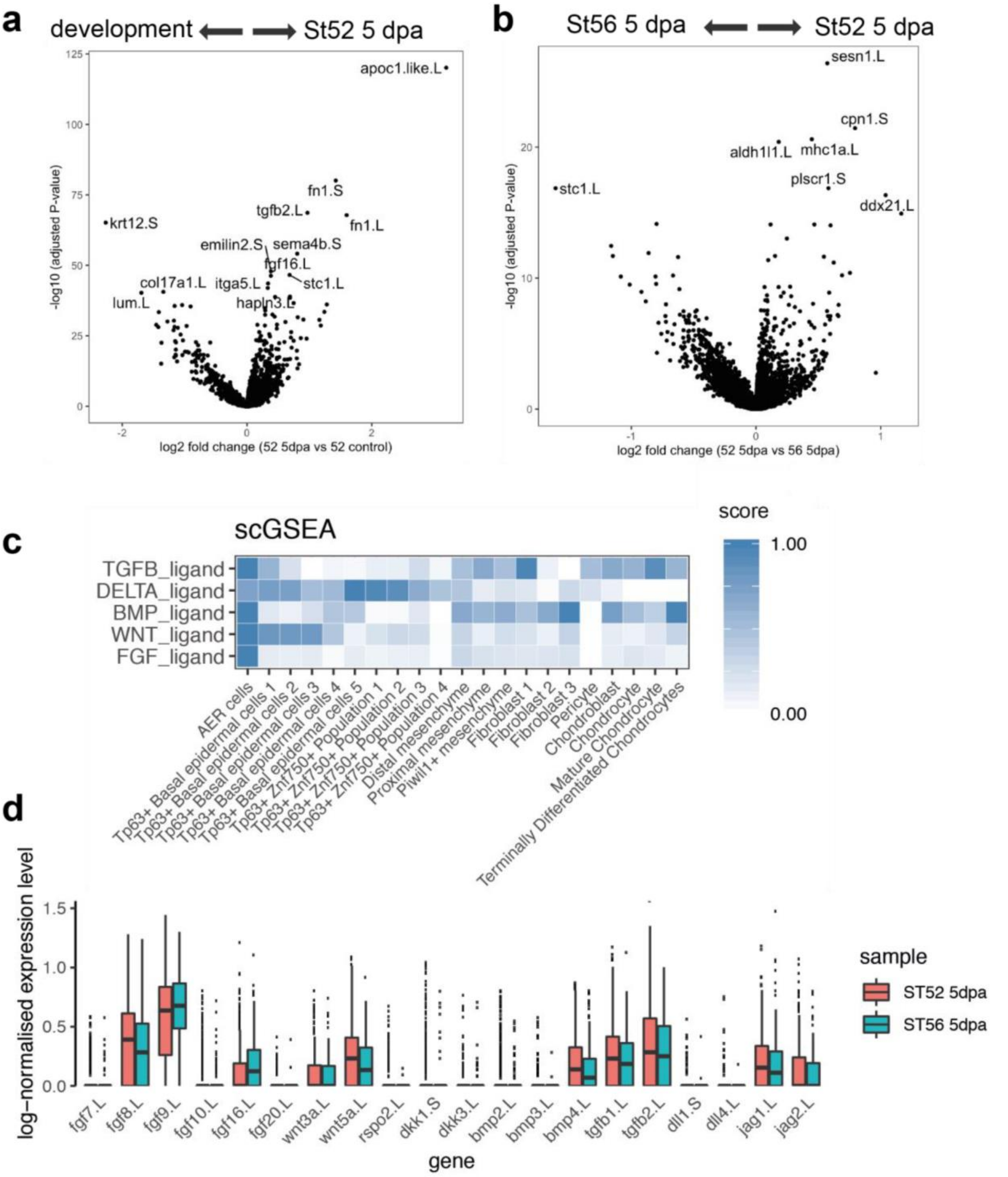

Differentially expressed genes are detected in AER cells between pairs of conditions and visualised using volcano plots. In **(a)**, Stage 52 5 dpa is compared to Stage 52 control samples; in **(b)**, Stage 56 5dpa is compared to Stage 52 5dpa samples. **(c)** Heatmap showing single-cell gene enrichment scores for ligands from the main signaling pathways are shown for epidermal cell types. AER cells have high signal center properties as they express high levels of TGF-B, Delta, BMP, WNT, and FGF ligands. Please see Supplementary Table 3 for the full list of ligands used in this analysis. **(d)** Log10-normalized gene expression visualised using boxplots to compare expression levels between Stage 52 5 dpa and Stage 56 5 dpa AER cells.

© 2021. Published by The Company of Biologists under the terms of the Creative Commons Attribution License (https://creativecommons.org/licenses/by/4.0/).

3- We further comment on the observation that “AEC tissue” is not composed of the same cell type for a limb and a tail in our discussion, and potential implication for other species.

In discussion:
Main text lines 454- 460
“Moreover, by identifying transcriptomic and morphological differences between the specialized wound epidermis of an amputated tail and limb, we demonstrated that, at the cellular level, appendage-regeneration is context-dependent and warrants caution for cross-paradigm comparisons. Indeed, it is likely that other regeneration-paradigms may use different cell types and transcriptional programmes for their specialised wound epidermis (e.g. zebrafish fin AEC does not express Fgf8 (Shibata et al., 2016)).”

- If some of the putative “dedifferentiation” genes identified in salamander studies are already expressed in Xenopus limb buds at the regeneration-competent stages, in the mesenchyme, what does this mean? Can the authors provide an alternative explanation grounded in the idea that these limbs are simply *still* developing, i.e., they are still expressing some of the genes that are characteristic of cells that are not fully differentiated yet? This issue relates to the earlier one I mentioned about making it more clear to readers that this kind of regeneration is predicated on the limb not actually being fully formed/differentiated/developed yet. Still, it is interesting to think about, and framing it as suggested does not mean this work will not be welcomed, digested, debated, and used productively by the research community.

We are somewhat confused with this comment of the reviewer, but try to address it below.

Firstly, in this study, we are not just working with developing limb buds – limb bud stages end at late NF Stage 53. In fact, the data we included related to “dedifferentiation” genes specifically involve NF Stage 55-56 and 58-60 tadpoles. NF Stage 56 and beyond tadpoles have patterned limbs and are in their secondary growth, a significantly different process to limb bud development. Moreover, based on our sequencing experiment (Fig 4C) and in alignment with Dent’s (1962) histology study, stages beyond Stage 56 do not contain progenitor cell types. We particularly highlight the Fibroblast 1 cluster, which resembles a differentiated cell type (we also see they are more abundant in late stage tadpoles).

We would like to clarify the reason we included this analysis in our manuscript. Recent research mostly focused on characterizing “dedifferentiation” genes and their link with regeneration. We particularly mention two separate phenotypes related to these genes as we think these findings may have important contributions to the field that cannot be easily addressed by previous methods (e.g. ISH) and with always-regenerating animals such as salamanders.

(1) We note some of the previously identified dedifferentiation genes are already expressed before amputations (Fig S10B). This phenotype does not seem to be related to developmental age or limbs being “developing” as the reviewer suggests. If this would be the case, all of these gene expression profiles would show decreasing expression in later stage samples as they would be more “developed”. However, we do not see such a phenotype, rather we see that gene expressions levels are largely maintained for the Fibroblast 1 cluster. We do not know why previous methods were unable to capture this phenotype. It could be due to technical limitations of ISH (which is the most commonly used method to identify gene expression patterns). Via scRNA-Seq, we can confidently distinguish cells that have high or low gene expression levels with specific population information. Alternatively, species differences could have a role in this phenotype, as the majority of these genes have been studied/identified in salamanders. Indeed, such species difference may have a role as we noted in line 591-595, unlike in salamanders where all diverse fibroblast populations were suggested to activate “dedifferentiation” genes, we see such dedifferentiation genes are activated only in a subset of fibroblasts.

Main text line 594-598
“Additionally, in contrast to axolotl limb regeneration, where a more homogenous mesenchymal transcriptional response was suggested (Gerber et al., 2018), we identified only a subset of fibroblast populations can gain transcriptional multipotency and express genes associated with

© 2021. Published by The Company of Biologists under the terms of the Creative Commons Attribution License (https://creativecommons.org/licenses/by/4.0/).

blastema.
Whether these differences between species result in more robust regenerative abilities requires further work."

(2) We identify that some of these "dedifferentiation" genes are upregulated upon injury (Fig S10A). This phenotype does not seem to have any association with developmental stage of the animal, and these genes are mostly not expressed before amputations but strongly expressed upon amputations regardless of regeneration-competency. As these genes were mostly characterized in salamanders, which can always regenerate, there was no amputation injury control in the experiments. By using varying regeneration-competency levels of Xenopus, we can discriminate injury-responses from true- regeneration specific gene expressions, which would be valuable for the field.

We discussed potential implications of this data in our discussion line 534-560.

"Benefiting from the stage-dependent regeneration-competency in Xenopus, our scRNA-seq datasets can discriminate true regeneration responses from injury responses. The majority of limb regeneration associated genes are derived from salamanders, where an injury control is not necessarily available (as these animals can always regenerate their limbs). We found that many genes associated with salamander limb regeneration (e.g. Dpt, Prdx2) (Gerber et al., 2018; Haas and Whited, 2017; Leigh et al., 2018) are upregulated upon injury in a subset of fibroblasts, regardless of regeneration competency. In a different context, recent single-cell analysis of mouse digit tip amputations suggests that, independent of the regeneration-outcome, some fibroblast populations express blastema-associated genes (e.g. Prickle1, Fbn2, Lrrc17) (Storer et al., 2020). We also see these genes upregulated upon injury in a subset of fibroblasts, but again this response is not specific to regeneration. These results suggest that there may be a conserved response to injury for mesenchymal cells in amphibians and mammals, and may be reflecting early suggestions by Tassava et al., that an injury can induce morphologically assigned "dedifferentiation" that fails to establish a blastema without a specialized wound epidermis (Tassava and Loyd, 1977; Tassava and Mescher, 1975). Indeed, we observed lower levels of some regeneration-associated distal mesenchyme genes (e.g. Shh) in the subset of fibroblasts when there are no AER cells (Figure S10), correlating with regeneration-competency. Nonetheless, our results are insufficient to determine: (1) whether the fibroblast cells progressively become intrinsically incompetent to fully dedifferentiate or (2), without signals from signaling center potent AER cells, they fail to fully dedifferentiate. Moreover, although a subset of fibroblasts can express genes from multiple lineages, the functional consequences of this gain of transcriptional multipotency and how it resolves during varying stages of regeneration- competency remain unclear. Further work on injury-induced mesenchymal plasticity, its interaction with AER cells, and cross-species comparison on this topic will be required. Nevertheless, these results underscore that caution is needed when interpreting experiments involving injury (e.g. transplantation), as well as the concern that previously implied "regeneration-genes" may be injury response genes. "

- Relatedly, this sentence is true, but it's also true that "its extent" tracks with animal age: "Together, we concluded that, upon amputation, a subset of fibroblasts manifest injury-induced mesenchymal plasticity – at least at the transcriptional level – and its extent tracks with AER cell abundance." It's a correlation and should be presented as such.

We are sorry that our word choice did not make it clear. We specifically used the word "tracks" as a sign of correlation but not a causation. We have now changed this sentence to "correlated" with AER cell abundance.

Revised sentence:
Main text: 256-258
" Together, we concluded that, upon amputation, a subset of fibroblasts manifest injury-induced mesenchymal plasticity - at least at the transcriptional level - and its extent correlates with AER cell abundance."

We agree with the reviewer that there is an inverse correlation between animal maturation and injury induced mesenchymal plasticity phenotype. We describe that regeneration-competency and

© 2021. Published by The Company of Biologists under the terms of the Creative Commons Attribution License (https://creativecommons.org/licenses/by/4.0/).

animal maturation are inversely correlated in the introduction (line 104-123). Hence, the suggested correlation by the reviewer (animal maturation and injury induced mesenchymal plasticity) is already given in our manuscript. We think specifically repeating this in the mentioned part of the manuscript would be confusing for the reader – as this, and other correlations, are already mentioned in the introduction and there are no animal maturation comments throughout the text.

Also, there is no true lineage/fate mapping here since it's just single-cell RNAseq, so we can't really conclude what they have stated. Even revising to this would convey the more accurate conclusion: "Together, we concluded that, upon amputation, a subset of fibroblasts may be stimulated to express markers known to track with regenerative success in urodeles, and this expression increase at the transcriptional level tracks with AER-cell-like abundance in wound epidermis and inversely tracks with animal maturation."

We thank the reviewer for alerting us to this issue! We are aware of the misconceptions in the literature associated with using scRNA-Seq to infer lineage decisions. Due to this, we tried to pay extra attention in interpreting and reporting these data. Here, for mesenchymal cells, we did not infer lineage or cell fate decisions. We detect these as a subset of fibroblast cells that show gene expressions associated with multiple cell fates. This transcriptional response may suggest that these cells have gained an ability to differentiate into different lineages, which would be consistent with some work in salamander and froglet. However, we do not have any functional data with tadpoles to claim this. Due to this, we specifically stated "- at least at the transcriptional level -" which is a statement we can make with the data we have. We would like to stress that "lineage decision" and "transcriptional plasticity" although very similar topics, do not mean the same, and recent single-cell transcriptomics results further create discussions on this topic.

To make our limitations and points more clearly, and to alleviate the concern of the reviewer, we have now included a sentence as below.

Revised discussion:
Main text line 554-556
"Moreover, although a subset of fibroblasts can express genes from multiple lineages, the functional consequences of this gain of transcriptional multipotency and how it resolves during varying stages of regeneration-competency remain unclear."

- An overt comparison between AER cells (from early limb bud) and the wound epithelium cells proposed to be called "AER" cells should be performed. The only analysis presented to support their claim includes all the other cell types . . .

We thank the reviewer for the comment. We have now conducted differential expression analysis between AER cells during development and during 5 dpa for regeneration-competent tadpoles, and we described this analysis in detail above (lines 865-877, 1001-1008).

- The work published in Leigh et al., 2018, is mentioned, but the specific findings about wound epidermis are not. Since this manuscript is centered on regeneration-competent wound epidermis, the authors should relate their findings to that data as well.

We thank the reviewer for this comment. We wished to compare our results to the wound epidermis results of Leigh et al. However, in their manuscript, they do not seem to detect a separate *Fgf8*+ epidermal population. They say that they found *Dlx3* in basal epidermal cells, but we observe that *Dlx3* is not solely expressed in AER cells but also in other populations. Moreover, they do not provide additional information. They also state in their methods that they remove wound epidermis in their sequencing experiments as it was creating technical problems for them, which may have influenced their results (we reproduce below sentences from the methods section of their manuscript):

From Leight et al 2018:
"…due to small blastema cell quantity in the early-bud blastemas, the entire WE was removed, and for medium-bud blastemas, the WE was removed and only half of the WE was put into the

© 2021. Published by The Company of Biologists under the terms of the Creative Commons Attribution License (https://creativecommons.org/licenses/by/4.0/).

collection tube…“

We also note that Reviewer 1 has specific comments on Fgf8 expression during salamander limb regeneration. While answering their comment, we summarized results from current single-cell results in axolotl – not just Leigh et al,- , and we would like to invite the reviewer to consider our response, which we copy below:

“In summary, Han et al. (Dev Dynamics, 2001) showed that there are *Fgf8* expressing epidermal cells at the basal layers of AEC during axolotl limb regeneration. This description parallels early work of Tassava where he started characterizing molecular properties of AEC tissue (*e.g.* Nace et al 1995) and highlighting the proposal that the basal layer could contain cells critical for regeneration. Very recently, Vincent et al. (Development, 2020) showed *Fgf8* expressing cells in different parts of the AEC during limb regeneration, not just basal layers. Han et al and Purushothaman showed that *Fgf8* is not expressed during limb development in axolotl, which parallels early findings where axolotls were suggested to not have morphologically identified AER during their development, but they have a ridge structure during regeneration (Tank et al. 1977). In the Nacu et al. study, the focus was mostly on an accessory limb model (which is considered as a model for normal limb growth or regeneration by some groups), and *Fgf8 in situs* did not show any labelling. In Gerber et al., 2018, we could not see where *Fgf8* is expressed in the published version of the manuscript (other than mesenchymal cells); but perhaps the reviewer may be referring to deposited data?. Still, even in the Gerber et al study, the cell numbers analysed are very low (with a focus on connective tissue cell types), which may be another reason that is not reported in the main manuscript. Lastly, we would like to note that there are 4 more axolotl limb regeneration single-cell transcriptomics studies in the literature (3 of them published very recently). Data from Leigh et al (Nat Comm, 2018), and Li et al (Protein Cell, 2020) were not able to detect a separate *Fgf8*+ cell cluster and, in these studies, there is no new cluster emergence upon limb amputations that could be labelled as AEC. Qin et al (Cell Death Differ, 2020) used single-cell transcriptomics and suggested identification of “AEC” as a separate cluster upon limb amputations. However, it was not reported whether this population expresses *Fgf8*. In the Leigh, Li, and Qin papers, the authors comment on basal epidermis clusters. However, the marker genes reported for these populations (e.g. Krt12) are not unique to AER cells in our study. The fourth study is from Rodgers et al (Experimental Cell Research, 2020) was conducted only at post amputation samples, the authors did not report Fgf8+ epidermal cell cluster, and the information about epidermal cell types is very limited. Some ambiguity may be due to using different methods (e.g. different dissociation protocols, staining protocols, sequencing platforms) and some might be due to biological reasons (e.g. using different age animals, using accessory limb model).

Collectively, we think that these studies show us that there is still an evolving understanding of the salamander AEC and its potential similarity (or difference to) a development AER program. We certainly agree that these data represents an important perspective to understanding the AER-AEC analogy, and have edited the main text accordingly.“

- The ex vivo experimentation might actually be the most compelling part of this paper. Noticing that there is not “bidirectional” regeneration when the embryonic limbs removed, explanted, and cultured, and then going a bit further and asking what’s happening on the end that formerly connected to the body is interesting. It’s also noteworthy that the authors did use chemical inhibitors of five different signaling pathways—even if most had already been demonstrated to be involved in limb regeneration—in this ex vivo context. This means that the modulation of these pathways is local to the amputation site, which advances our understanding of the overall process.

We thank the reviewer for this comment. We are also excited to see that limb has autonomy and many different experiments can be conducted with explants!

- Some of the previous work on where wound epidermis cells come from needs to be dealt with in the manuscript. There are many examples of previous work that supports the notion that they are derived from pre-existing epidermal cells left on the stump, but none of them are referenced or discussed. A good place to start is the seminal work of Elizabeth Hay, for example, Hay and Fischman, 1961, in newts.

All reviewers commented on this topic, and we thank them for raising this issue. We now cite

© 2021. Published by The Company of Biologists under the terms of the Creative Commons Attribution License (https://creativecommons.org/licenses/by/4.0/).

suggested key paper from Hay and Fischman, 1961 and a review paper on wound epidermis by Campbell and Crews, 2008, citing other relevant studies.

As Reviewer 3 also notes, we do not know the properties of *Xenopus* AEC as much as we know in salamanders. Additionally, molecular characterization of *Xenopus* limb explants was not performed previously. Critically, in the aforementioned studies, the specialised wound epidermis was investigated as a *tissue*, without characterizing its specific cell types. Here, we specifically tested if AER cells within specialised wound epidermis *tissue* are formed after cell division. For this, we stained against *Fgf8* mRNA which corresponds to a specific population within the basal layer of AEC tissue, and check for EdU co-stained cells. On another note, we note that our scRNA-Seq data also suggests that AER cells are not very proliferative (Fig S2D). Moreover, not just the salamander AEC, but also the mouse AER has been suggested to not proliferate much (Storer et al, Cell, 2013). We have edited our text and now also include all this information as well.

Revised texts:
Main text line: 294-319
"Next, we asked how AER cells form on the amputation plane. It has been suggested that salamander AEC tissue forms by migration of epidermal cells to the amputation plane, and does not require cell proliferation (Campbell and Crews, 2008; Hay and Fischman, 1961). Moreover, the mouse AER was previously suggested to be a largely mitotically inactive tissue (Storer et al., 2013). However, it is not known whether similar mechanisms apply to AER cells within the specialised wound epidermis, and also to what extent they are seen in Xenopus. Therefore, we first traced skin tissue located on the edge of explants, and found that they contributed to the covering of both the distal and proximal sites (Figure S13B). As the amputation planes are covered by skin tissue from the surrounding area, we reasoned that AER cells are likely to have originated from skin cells. As amputation eliminates the majority, if not all, of AER cells in the limb, we hypothesized that AER cells are derived from remaining skin stem cells. If AER cells are induced through proliferation and differentiation following amputation, all AER cells should be the product of cell division. To test this hypothesis, we assayed the level of EdU incorporation (labelling newly synthesized DNA, hence divided cells) in newly-formed AER cells, using Fgf8 positivity to specifically identify AER cells within the AEC tissue. We found that only ~40% of AER cells (distal epidermal Fgf8+) were EdU positive at 3 dpa (Figure S13C), suggesting that most AER cells are induced independently of cell division following amputation. These results parallel our transcriptomics-based cell-cycle assessment in which AER cells display low levels of proliferation (Figure S2D). Using the transcriptomics data, we identified a stepwise activation of Lgr5.S (a WNT target gene) followed by Fgf8.L expression as a possible gene-expression trajectory that could allow basal epidermal cells to convert directly to AER cells without cell division (Figure 3D). Consistent with such a process, when visualized in vivo, we found that Fgf8+/Lgr5+ AER cells were flanked by Lgr5+ cells in the basal epidermis on the amputation plane or in the developing limb (Figures 3E and S7A-B). Overall, these results support the hypothesis that basal epidermal cells can acquire AER cell identity without cell division. "

- Figures 3 and 4 are, in my opinion, the most novel in the paper. One concern, though, about interpreting the data as they have done in 4C with the bar graphs is that we don't really know if the different cell types are differentially amenable to the dissociation, encapsulation, and sequencing protocols or if their data truly reflects a different relative composition of these cell types in the samples from different stages. Certainly tissues at 5dpa, particularly distal ones, are looser and more apt to go through the pipeline successfully. Some acknowledgement of this limitation would be helpful to others doing these kinds of experiments, and it might also (rightly so) give us pause in interpreting them.

This is a very valid point that is also very hard to validate in the lab. In fact, due to similar concerns raised by the reviewer, we did not include regeneration-incompetent intact limbs in our study, as we were not able to properly dissociate them, which would limit our interpretation and could mislead us. However, we do not have any data to question the validity of data presented in Fig 4C. Although not at the same resolution, our cell type abundance graph in 4C resembles what has been described by Dent in his 1962 study (e.g. more chondrogenic lineage cells, less progenitor looking cells after certain stages). We now also refer to this paper as described below.

© 2021. Published by The Company of Biologists under the terms of the Creative Commons Attribution License (https://creativecommons.org/licenses/by/4.0/).

Revised result:
Main text lines: 365-368
"We found that that the loss of regeneration potential is associated with an increased proportion of chondrogenic lineage cells in the mesenchyme (Figure 4C, aligning with previous tissue-level observations (Dent, 1962)) and that these cells express multiple inhibitory ligands for BMP and WNT pathways (Figure 4D)."

- Perhaps the major shortcoming of the experiments is that while the authors could show that a larger fraction of fgf-8-expressing cells either exists or can be captured and sequenced following treatments that inhibit Noggin, there is no direct regeneration outcome to mirror this finding. The existence of the fgf-8- expressing cells has to suffice as the proxy. To demonstrate that mesenchymal tissues become more activated, and therefore show a greater regenerative response, they should repeat the experiments and follow with a more direct assay. This could be something simple like EdU incorporation in the mesenchymal tissues, but it could also be something else. How long can the cultures be maintained? Is it possible to measure outgrowth? Even with respect to the fgf-8- expressinng epidermal cells, why are there fewer? Can an experiment be performed to distinguish between less recruitment of fgf-8-expressing cells to the wound epithelium versus lower amounts of proliferation of the fgf-8-expressing cells that do reside in the wound epithelium? Do all of the pathways act through the same mechanism?

We recognize there are many separate questions within this point, so we will answer them in sequence:

"Perhaps the major shortcoming of the experiments is that while the authors could show that a larger fraction of fgf-8-expressing cells either exists or can be captured and sequenced following treatments that inhibit Noggin, there is no direct regeneration outcome to mirror this finding. The existence of the fgf-8- expressing cells has to suffice as the proxy. "To demonstrate that mesenchymal tissues become more activated, and therefore show a greater regenerative response, they should repeat the experiments and follow with a more direct assay. This could be something simple like EdU incorporation in the mesenchymal tissues, but it could also be something else."

We thank the reviewer for this comment. We now include new experiments that more directly test the regeneration outcome mirroring our previous findings. As we discussed in our manuscript, we cannot remove/transfer all secreted factors together *in vivo*. However, by focusing on a single inhibitor (*Noggin*), we performed two more direct experiments to investigate the *in vivo* function of the secreted inhibitors, and we present them below.

First, in an effort to reduce the amount of a secreted inhibitor, we implanted anti-NOGGIN beads on the amputation plane of regeneration-restricted/incompetent tadpoles *in vivo* and tested the regeneration-outcome. When we conducted this experiment, we observed a slight improvement in the number of formed digits/digit-like structures, further supporting the hypothesis that secreted inhibitors limit regeneration-competency in late stage tadpoles. We see that this perturbation has a mild effect compared to our FGF10 bead rescue experiment in regeneration-restricted/incompetent tadpoles (**Figure 5A**). We do not know if this mild effect is due to technical problems with the perturbation or due to biological reasons. These could be: (1) diffusivity of antibodies from beads is limited and causing a milder phenotype; (2) beads are repelled within a day, and hence there would be a transient Noggin removal – meanwhile according to our other findings and model, blocking FGF10 would have a longer effect compared to this transient Noggin removal; and (3) Noggin is one of the secreted inhibitors but it is likely others are playing a role as well (including Chrdl1 and Frzb that are still highly expressed in chondrogenic lineage) **(Figure 4D)**. Hence, anti-NOGGIN beads may have only removed a small part of the overall inhibition. Meanwhile, implanting FGF10 would block secretion of the other secreted inhibitors as well since it blocks the cellular source of them (chondrogenesis). We now include this experiment in results and discuss potential caveats in the discussion, as indicated below.

Revised in results:
Main text line 378-383
"Based on these observations, we then explored whether anti-NOGGIN application would improve the in vivo amputation response. Indeed, when beads loaded with anti-NOGGIN antibodies were implanted on the amputation plane of regeneration-restricted/incompetent tadpoles, we saw a

© 2021. Published by The Company of Biologists under the terms of the Creative Commons Attribution License (https://creativecommons.org/licenses/by/4.0/).

mild improvement in the regenerative response (Figure 5A), highlighting that secreted inhibitors are influencing the regeneration-outcome in vivo. "

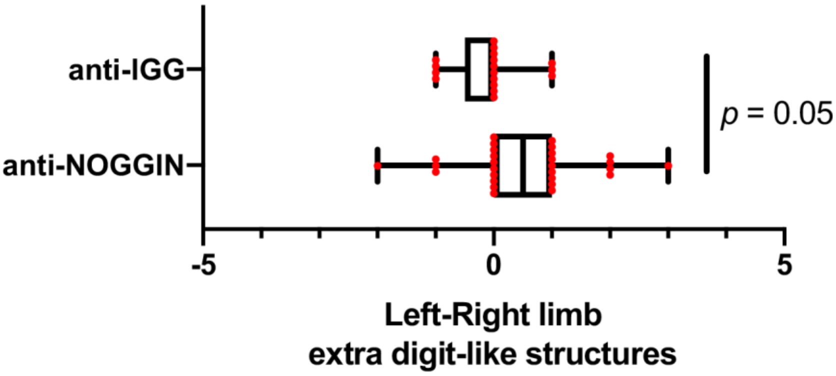

Anti-NOGGIN antibody application to distal amputations improve regeneration in –restricted and –incompetent tadpoles. –Restricted and –incompetent tadpole right and left hindlimbs were amputated and beads containing anti-IGG antibody or anti-NOGGIN antibody were placed on the right hindlimbs. Formed digits and digit-like structures were quantified in the right and left hindlimbs and the difference calculated. Anti-IGG antibody total n=17 from 3 biological replicates; Anti-NOGGIN antibody total n=28 from 4 biological replicates.

Second, based on our *ex vivo* experiments (Fig 5F), we expected NOGGIN would operate downstream of the effect of FGF10. However, we did not test this hypothesis *in vivo* at the time of our initial submission. As reported by Yokoyama et al, we demonstrated that FGF10 can restore regenerative abilities in regeneration-restricted and –incompetent tadpoles. In the current study, we also conducted co-application of FGF10 + NOGGIN beads to the amputation plane of regeneration- restricted and –incompetent tadpoles *in vivo*. We found that co-application of FGF10 + NOGGIN decreases the positive effect of FGF10 only beads, corroborating our *ex vivo* findings (Fig 5E) and suggesting that the secreted inhibitors may be the "bottle-neck" event driving regeneration-outcome. We now mention this experiment in our results, and hope that the reviewer considers these experiments to further strengthen the findings in this study.

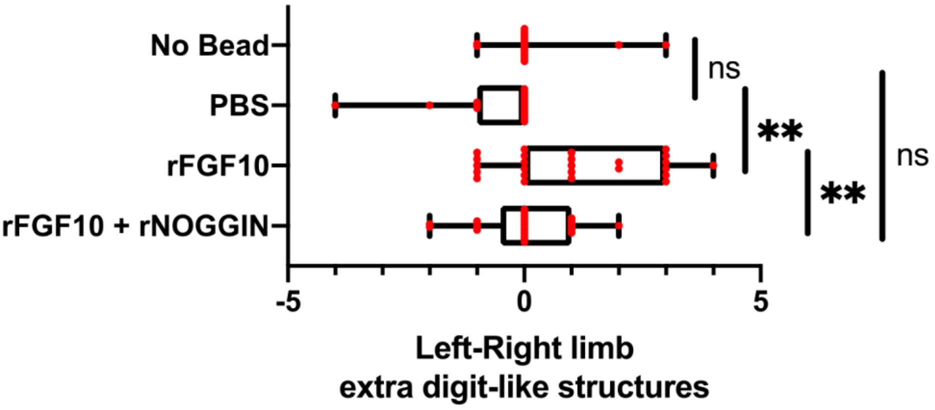

Recombinant FGF10 application to distal amputations restore regeneration in –restricted and –incompetent tadpoles. –Restricted and –incompetent tadpole right and left hindlimbs were amputated and beads containing 0.1% BSA/PBS or recombinant FGF10 or recombinant FGF10 and NOGGIN were placed on the right hindlimbs. Formed digits and digit-like structures were quantified in the right and left hindlimbs and the difference calculated. Empty total n=19 from 2 biological replicates; 0.1%/PBS bead total n=17 from 5 biological replicates; recombinant FGF10 bead total n=25 from 5 biological replicates; recombinant FGF10 and NOGGIN bead total n=25 from 4 biological replicates. ns = not significant, *P***< 0.001.

Revised in results:
Main text: 436-442
"To further test this finding in vivo, we asked if the positive effect of FGF10 in regeneration-

© 2021. Published by The Company of Biologists under the terms of the Creative Commons Attribution License (https://creativecommons.org/licenses/by/4.0/).

incompetent tadpoles could be abrogated by simultaneous NOGGIN addition. For this, we inserted beads co-loaded with FGF10 and NOGGIN to the amputation plane of regeneration-restricted/incompetent tadpoles and found this significantly decreased the positive effect of FGF10- only beads (Figure 5G). These results further emphasise that FGF10 operates upstream of NOGGIN, and hence that secreted inhibitors play a dominant role in determining regeneration-outcome."

Revised in discussion:
Main text lines 505-514
"We then tested our model in vivo and found that indeed removal of secreted inhibitors (e.g. NOGGIN), or blocking the source of secreted inhibitors (chondrogenic progression via FGF10 application) could improve the regeneration-outcome in regeneration defective stages. Moreover, we demonstrated that NOGGIN cancels the positive effect of FGF10 application, further highlighting the downstream role played by the secreted inhibitors. Overall, these results align with previous transplantation experiments showing that mesoderm from regeneration-incompetent limbs is inhibitory to regeneration (Sessions and Bryant, 1988; Yokoyama et al., 2001, 2000). However, in contrast to previous interpretations, we suggest that an important contributor to this phenomenon is the enrichment of chondrogenic cell abundance within the mesoderm tissue which express inhibitory secreted factors."

Revised discussion:
Main text line: 516-523
"We further showed that by manipulating NOGGIN and FGF10 levels we could improve amputation-outcomes in regeneration-restricted/incompetent tadpoles. We see that anti-NOGGIN beads have a mild effect compared to FGF10 beads (Figure 5A and 5G), which may suggest that there are other inhibitors secreted from the chondrogenic lineage (e.g. Chrdl1, Frzb) that must also be eliminated to ensure robust regeneration. However, the mild effect of anti-NOGGIN may also be due to technical problems with the perturbation (e.g. limited duration and/or diffusivity of antibody delivery), and that a more complete inhibition of NOGGIN function would further improve the amputation-outcome."

Revised methods:
Main text line: 711-719
*"Affi-gel blue gel beads (Bio-rad, **1537301**) were incubated with the following proteins overnight at 4 degrees: 2-3 µg Rabbit-IGG isotype control antibody (ab37415); 2-3 µg anti-NOGGIN antibody (ab16054); 0.1% BSA; 1 µg recombinant human FGF10 (R&D, 345-FG) in 1-2 µl 0.1% BSA; 1-1.5 µg recombinant human FGF10 (R&D, 345-FG) and 2.5-4 µg recombinant human NOGGIN (R&D, 6057-NG) in 3-4 µl 0.1% BSA. Tadpoles were anaesthetized with 0.002% MS222, placed on a wet towel, and both right and left hindlimbs were amputated from ankle level in either -restricted or -incompetent tadpoles. 3-4 beads were placed on the amputation plane of the right hindlimb. Left hindlimbs served as an internal control for the experiments."*

We hope the reviewer also agrees these experiments test our hypothesis with more direct assays and strengthens the findings in this study.

"How long can the cultures be maintained? Is it possible to measure outgrowth?"

This is a very exciting question! We are currently working on extending culture conditions that would enable us to measure outgrowth. However, in the scope of this work, we feel this is not necessary to include these experiments, as the 3-day culture allows us to investigate AER cell formation, which is the main topic of this study.

"Can an experiment be performed to distinguish between less recruitment of fgf-8-expressing cells to the wound epithelium versus lower amounts of proliferation of the fgf-8-expressing cells that do reside in the wound epithelium?"

As we stated in our manuscript, amputations practically remove all AER cells that we can detect. There might be undetected single AER cell clones, although we have no evidence for this statement. In our work, we tested AER cell formation at 3 dpa, and it would be very hard for a few clones to proliferate and reach the numbers that we see at 3 dpa. Moreover, in such a scenario, we

© 2021. Published by The Company of Biologists under the terms of the Creative Commons Attribution License (https://creativecommons.org/licenses/by/4.0/).

would also expect to see the large majority of AER cells as EdU+, which we do not observe. Hence, the "recruitment" of AER cells, or "recruitment and expansion" of AER cells do not seem plausible hypotheses. Meanwhile, our scRNA-Seq results (Fig S2D) and EdU experiments (Fig S13B) suggest that AER cells are not a very proliferative population. Hence, these results lead us to conclude that it is the AER cell specification that becomes problematic during *Xenopus* development.

"Do all of the pathways act through the same mechanism?"

We are not sure which pathways the reviewer refers to here.

If the pathways mentioned here are FGF, WNT, BMP, NOTCH, and TGFB (as considered experimentally in our study), it might be possible that AER cell identity would directly require activity of all these pathways. We do see that the downstream elements of all of these pathways are upregulated from basal epidermal cells to AER cell identity (based on scRNA-Seq and Lgr5 HCR results). Nonetheless, our experiments do not allow us to dissect whether the effect is direct or indirect, and we have changed our text to make it this point clearer.

Revised results
Main text lines 288-290
"Overall, we concluded that AER cell formation requires the activity of multiple major signaling pathways, although further work is required to determine what roles these pathways play and whether they directly or indirectly regulate AER cell formation."

- Interpretation of the wound epidermal (so-called "AER" cells) as a "signaling center population"—this is a bit dubious to call these such on the basis of them expressing the five ligands called out in Fig.
S9. There are so many other signaling molecules known, and certainly many more molecules may act as signaling molecules than are already characterized as such. In this heat map alone, all of the ligands included are substantially expressed in at least one other population that is not "AER." Further, it's unclear which genes these "ligand" molecules correspond to. There are lots of problems here that could be fixed by not drawing such broad conclusions. Also, where's the fgf-10 transcript being expressed among mesenchymal cells?

Thank you very much for this comment. It is our mistake and we should have given more details. The data on Fig S9 (new Fig S6) does not show 5 ligands. It shows cumulative scoring for many ligands from these five signalling pathways by using single-cell gene enrichment analysis. In fact, in this analysis, we calculate and score a total of 66 ligands. To prevent similar misunderstanding, we have now edited our new Fig S6 legend, leading to the added supplemental table 3 showing all these ligands. We also revised our legend for this figure as below.

*"**(c)** Heatmap showing single-cell gene enrichment scores for ligands from the main signaling pathways are shown for epidermal cell types. AER cells have high signal center properties as they express high levels of TGF-β, Delta, BMP, WNT, and FGF ligands. Please see Supplementary Table 3 for the full list of ligands used in this analysis."*

Regarding the comment that "In this heat map alone, all of the ligands included are substantially expressed in at least one other population that is not "AER.", Indeed at least one population not composed of AER cells secrete these ligand families at some level. However, only AER cells can secrete all of these ligand families altogether and at a very high level which, in our opinion, makes them a signalling centre.

Regarding the question about Fgf10: depending on the condition, we find that Fgf10 is expressed in different mesenchymal populations. Below, we plot the expression of Fgf10 across cell types. We have provided in our manuscript (lines 183 in results and in 895 in data and code availability) a website link where these can be checked easily, and specific sample expressions could be also visualised: https://marionilab.cruk.cam.ac.uk/XenopusLimbRegeneration/

© 2021. Published by The Company of Biologists under the terms of the Creative Commons Attribution License (https://creativecommons.org/licenses/by/4.0/).

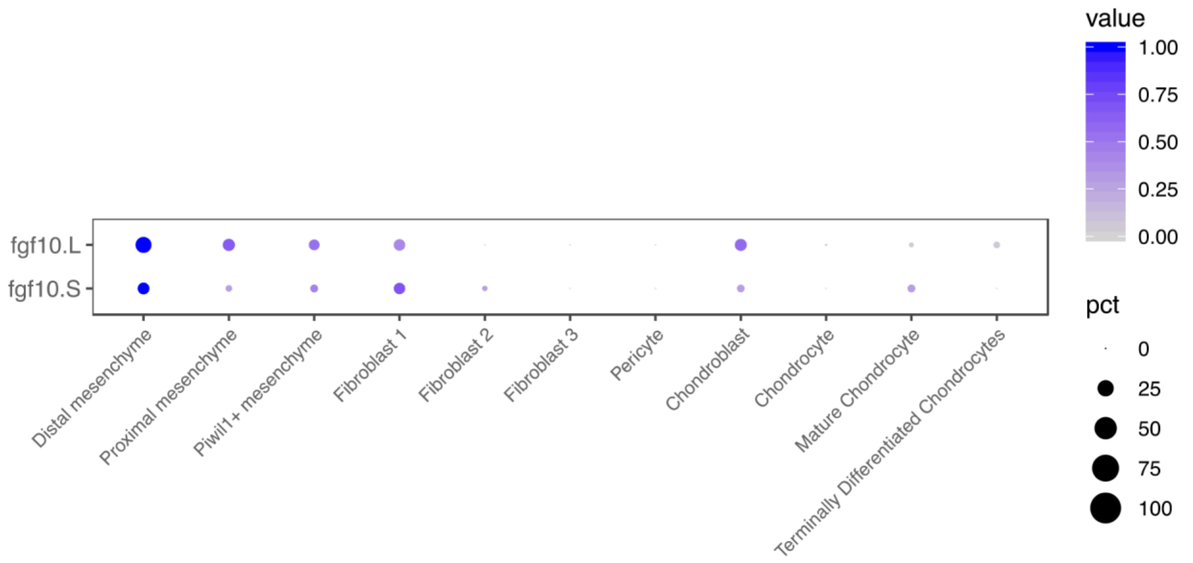

• Are there any other possible explanations for their ex vivo experiments, in toto, other then their interpretation that chondrogenesis (here, at the previously proximal end) inhibits regeneration?
This is an excellent opportunity to elaborate on other possible explanations without staking the claim so fiercely that it's chondrogenesis inhibiting it (and it seems that while the hypothesis is consistent, there are other viable explanations).

This is an excellent point. We would like to repeat below some of the observed phenotypes that lead us to our main hypothesis that "secreted factors from chondrogenic lineage blocks AER cell formation":

1- Our distal tip explant experiments lead to ectopic Fgf8 expression – Fig S15
2- Our observation that there are different behaviors in proximal versus distal sites of explants - the proximal site, being rich in more mature chondrogenic cells, does not form AER cells – Fig 3
3- Our co-culture and conditioned media experiments - secreted inhibitors, specifically the ones expressed from chondrogenic lineage, block AER cell formation ability – Fig 4
4- Our *ex vivo* and *in vivo* experiments with Fgf10 and Noggin- Fgf10 operates upstream of the effect of Noggin, and more specifically FGF10 can suppress chondrogenesis which expresses Noggin– Fig 5, Fig S15,
5- From the literature, differences in regeneration-outcome for amputation to proximal sites versus distal sites, as the proximal site has more mature chondrogenic lineage cells compared to distal sites, Wolfe et al, Dent, Overton, Nye & Cameron
6- From the literature, differences in regeneration-outcome for amputation through bone versus through joint, as through bone would face more mature chondrogenic lineage cells compared to joints. Wolfe et al, Nye & Cameron
7- From the literature, Yokoyama et al and Bryant et al tissue grafting results, as tissue grafts would also transfer secreted factors and cell types contributing to it.

As the reviewer also points, all of these observations are consistent with our hypothesis. Unfortunately, we do not have an alternative hypothesis that can explain all these observed phenotypes. We hope that our study and discussions will bring more attention to this topic of why regeneration-incompetency arises in animals and what is the connection between chondrogenic progression, AER cell formation, and limb regeneration.

• Relatedly, if the authors want to provide more solid evidence that chondrogenesis per se inhibits limb regenerative capacity, then they should devise and execute more convincing experiments. For example, if they remove skeletal elements from the harvested limbs before they culture them, can they demonstrate that these limbs have a longer potential (time frame) for pro-regenerative responses? This experiment could also be attempted in vivo using microsurgery on the animals or possibly by laser ablation. Alternatively, a genetic ablation strategy could be employed.

© 2021. Published by The Company of Biologists under the terms of the Creative Commons Attribution License (https://creativecommons.org/licenses/by/4.0/).

Thank you very much for this comment! We would be more than thrilled to conduct such experiments and indeed considered trying them. However, we think that the suggested experiments have major caveats that would not enable us to confidently test the hypothesis that "chondrogenesis directly inhibits the regenerative capacity".

More specifically, the manual removal of skeletal elements or laser ablation would create: 1) additional inflammation – which is experimentally shown to block limb regeneration in Xenopus (Mescher et al, 2013); 2) additional injury, which is likely to influence cellular behaviors; 3) we may remove non-chondrogenic cell types; and 4) we may have incomplete removal of chondrogenic cell types. We know that such caveats have created many problems of interpretation in the field. Second, we agree that genetic cell ablation strategy would be the best approach and this is an approach with which our lab has experience. However, there are again certain technical limitations and caveats. In particular, we would need a system where we can **transiently ablate** chondrogenic lineage cells **only at the amputation plane** but no other proximal site or animal body - as permanently removing chondrogenic lineage cells, or systematically removing them would create problems with the developing/regenerated limb morphology and the development of the animal in general. Hence, we need both temporally and spatially controllable cell ablation method. Only very recently have such optogenetics controlled systems begun to be described in systems such as zebrafish (Mruk et al, Development, 2020), which are easier to manipulate, and their potential problems and whether such a system would work in *Xenopus* are unknown. Moreover, such genetic labelling involving cell ablation methods result in apoptosis lasting a couple of hours. By contrast, we need a system that works rapidly as we want all the secreted factors to disappear. Also, we would need to find a method in which we can also remove already secreted inhibitory factors residing in the limb before amputation. Lastly, even if we can find a system satisfying these conditions, we would need a full-transgenic tadpole to test this hypothesis, as we need to ensure that the ablation is complete. As the reviewer would know, it is time consuming to obtain full transgenic *Xenopus laevis* tadpoles, as it takes at least a year to raise a transgenic line and then 2 months to raise them to tadpoles stages to do this experiment. Due to these reasons, we were very careful with our interpretation and statements being limited to "chondrogenesis associated secreted inhibitors", rather than "chondrogenesis itself"; although, we have multiple circumstantial evidences supporting the latter conclusion (as listed above).

For the reasons outlined above, we believe that our results already support our conclusions, while we hope to be able to conduct further such experiments in the future. Nonetheless, thanks to this and one above comment, we now edited our text:

Revised discussion Main text
lines: 563-566
"Although it remains unclear if chondrogenesis itself directly inhibits limb regeneration, there are multiple observations from our work and others that support this hypothesis (Dent, 1962; Nye and Cameron, 2005; Wolfe et al., 2000)."

Reviewer 3 Advance Summary and Potential Significance to Field:
Understanding of the similarities/differences between the AER (development) and the AEC (regeneration), molecular basis of the formation of the AEC during Xenopus limb regeneration, potential reasons for the loss of regeneration competence in Xenopus limb stages.

Reviewer 3 Comments for the Author:
This is a very impressive series of experiments involving single cell RNA sequencing of developmental limb bud stages, regeneration competent, regeneration restricted and regeneration incompetent stages in Xenopus. This is not just a characterization of gene expressions by single cell, but it leads to experiments using in vitro limbs which identify the role of the AEC/AER in the transition from regeneration competency to incompetency and the role that chondrogenesis plays in inhibiting the formation of the AEC via the production of Noggin. Although the authors did not get regeneration incompetent stages to regenerate in vivo through noggin/fgf10 manipulation they did get it to happen in vitro in terms of increases in the length and proliferation and AEC production of cultured limbs. Very impressive. This work has great significance for our understanding of the decline of regeneration in Xenopus and possibly for "the potential to unlock the ability to regrow lost limbs in non-regenerative higher vertebrates" (lines 506-507).

© 2021. Published by The Company of Biologists under the terms of the Creative Commons Attribution License (https://creativecommons.org/licenses/by/4.0/).

We thank the reviewer for their very supportive comments on our experimental designs and results. In our revised manuscript, we now include two new in vivo experiments testing our hypothesis. As we discussed in our manuscript, we cannot remove/transfer all secreted factors together *in vivo*.
However, by focusing on a single inhibitor (*Noggin*), we performed two experiments to investigate the *in vivo* function of the secreted inhibitors hypothesis and we present them below.

First, in an effort to reduce the amount of a secreted inhibitor, we implanted anti-NOGGIN beads on the amputation plane of regeneration-restricted/incompetent tadpoles *in vivo* and tested the regeneration-outcome. When we conducted this experiment, we observed a slight improvement in the number of formed digits/digit-like structures, further supporting the hypothesis that secreted inhibitors limit regeneration-competency in late stage tadpoles. We see that this perturbation has a mild effect compared to our FGF10 bead rescue experiment in regeneration-restricted/incompetent tadpoles (**Figure 5A**). We do not know if this mild effect is due to technical problems with the perturbation or due to biological reasons. These could be: (1) diffusivity of antibodies from beads is limited and causing a milder phenotype; (2) beads are repelled within a day, and hence there would be a transient Noggin removal – meanwhile according to our other findings and model, blocking FGF10 would have a longer effect compared to this transient Noggin removal; and (3) Noggin is one of the secreted inhibitors but it is likely others are playing a role as well (including Chrdl1 and Frzb that are still highly expressed in chondrogenic lineage) **(Figure 4D)**. Hence, anti-NOGGIN beads may have only removed a small part of the overall inhibition. Meanwhile, implanting FGF10 would block secretion of the other secreted inhibitors as well since it blocks the cellular source of them (chondrogenesis). We now include this experiment in results and discuss potential caveats in the discussion, as indicated below.

Revised in results:
Main text line 378-383
"Based on these observations, we then explored whether anti-NOGGIN application would improve the in vivo amputation response. Indeed, when beads loaded with anti-NOGGIN antibodies were implanted on the amputation plane of regeneration-restricted/incompetent tadpoles, we saw a mild improvement in the regenerative response (Figure 5A), highlighting that secreted inhibitors are influencing the regeneration-outcome in vivo."

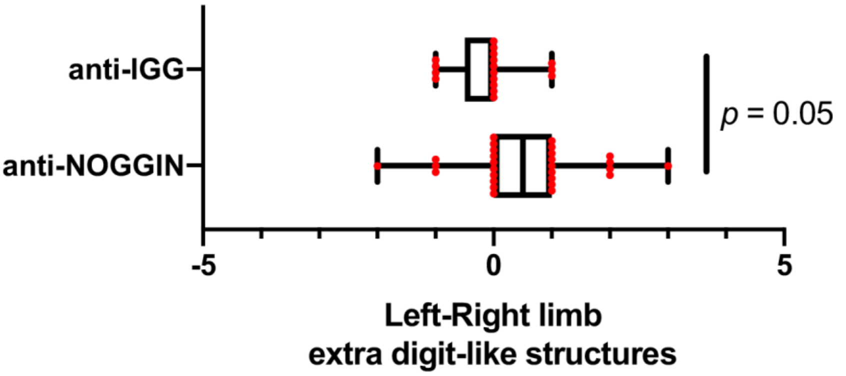

Anti-NOGGIN antibody application to distal amputations improve regeneration in -restricted and -incompetent tadpoles. -Restricted and -incompetent tadpole right and left hindlimbs were amputated and beads containing anti-IGG antibody or anti-NOGGIN antibody were placed on the right hindlimbs. Formed digits and digit-like structures were quantified in the right and left hindlimbs and the difference calculated. Anti-IGG antibody total n=17 from 3 biological replicates; Anti-NOGGIN antibody total n=28 from 4 biological replicates.

Second, based on our *ex vivo* experiments (Fig 5F), we expected NOGGIN would operate downstream of the effect of FGF10. However, we did not test this hypothesis *in vivo* at the time of our initial submission. As reported by Yokoyama et al, we demonstrated that FGF10 can restore regenerative abilities in regeneration-restricted and -incompetent tadpoles. In the current study, we also conducted co-application of FGF10 + NOGGIN beads to the amputation plane of regeneration- restricted and -incompetent tadpoles *in vivo*. We found that co-application of FGF10

© 2021. Published by The Company of Biologists under the terms of the Creative Commons Attribution License (https://creativecommons.org/licenses/by/4.0/).

+ NOGGIN decreases the positive effect of FGF10 only beads, corroborating our *ex vivo* findings (Fig 5E) and suggesting that the secreted inhibitors may be the “bottle-neck” event driving regeneration-outcome. We now mention this experiment in our results, and hope that the reviewer considers these experiments to further strengthen the findings in this study.

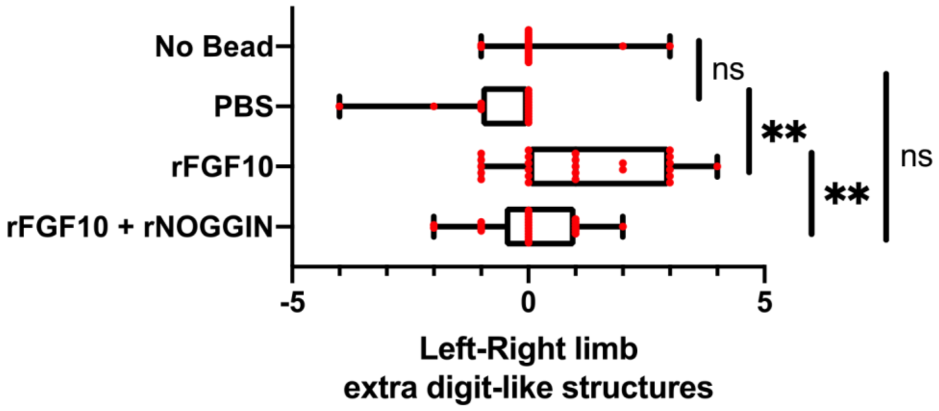

Recombinant FGF10 application to distal amputations restore regeneration in –restricted and –incompetent tadpoles. -Restricted and -incompetent tadpole right and left hindlimbs were amputated and beads containing 0.1% BSA/PBS or recombinant FGF10 or recombinant FGF10 and NOGGIN were placed on the right hindlimbs. Formed digits and digit-like structures were quantified in the right and left hindlimbs and the difference calculated. Empty total n=19 from 2 biological replicates; 0.1%/PBS bead total n=17 from 5 biological replicates; recombinant FGF10 bead total n=25 from 5 biological replicates; recombinant FGF10 and NOGGIN bead total n=25 from 4 biological replicates. ns = not significant, *P***< 0.001.

Revised in results:
Main text: 436-442
“To further test this finding in vivo, we asked if the positive effect of FGF10 in regeneration-incompetent tadpoles could be abrogated by simultaneous NOGGIN addition. For this, we inserted beads co-loaded with FGF10 and NOGGIN to the amputation plane of regeneration-restricted/incompetent tadpoles and found this significantly decreased the positive effect of FGF10- only beads (Figure 5G). These results further emphasise that FGF10 operates upstream of NOGGIN, and hence that secreted inhibitors play a dominant role in determining regeneration-outcome.”

Revised in discussion:
Main text lines 505-514
“We then tested our model in vivo and found that indeed removal of secreted inhibitors (e.g. NOGGIN), or blocking the source of secreted inhibitors (chondrogenic progression via FGF10 application) could improve the regeneration-outcome in regeneration defective stages. Moreover, we demonstrated that NOGGIN cancels the positive effect of FGF10 application, further highlighting the downstream role played by the secreted inhibitors. Overall, these results align with previous transplantation experiments showing that mesoderm from regeneration-incompetent limbs is inhibitory to regeneration (Sessions and Bryant, 1988; Yokoyama et al., 2001, 2000). However, in contrast to previous interpretations, we suggest that an important contributor to this phenomenon is the enrichment of chondrogenic cell abundance within the mesoderm tissue which express inhibitory secreted factors.”

Revised discussion:
Main text line: 516-523
“We further showed that by manipulating NOGGIN and FGF10 levels we could improve amputation-outcomes in regeneration-restricted/incompetent tadpoles. We see that anti-NOGGIN beads have a mild effect compared to FGF10 beads (Figure 5A and 5G), which may suggest that there are other inhibitors secreted from the chondrogenic lineage (e.g. Chrdl1, Frzb) that must also be eliminated to ensure robust regeneration. However, the mild effect of anti-NOGGIN may also be due to technical problems with the perturbation (e.g. limited duration and/or diffusivity of antibody

© 2021. Published by The Company of Biologists under the terms of the Creative Commons Attribution License (https://creativecommons.org/licenses/by/4.0/).

delivery), and that a more complete inhibition of NOGGIN function would further improve the amputation-outcome."

Revised methods:
Main text line: 711-719
*"Affi-gel blue gel beads (Bio-rad, **1537301**) were incubated with the following proteins overnight at 4 degrees: 2-3 µg Rabbit-IGG isotype control antibody (ab37415); 2-3 µg anti-NOGGIN antibody (ab16054); 0.1% BSA; 1 µg recombinant human FGF10 (R&D, 345-FG) in 1-2 µl 0.1% BSA; 1-1.5 µg recombinant human FGF10 (R&D, 345-FG) and 2.5-4 µg recombinant human NOGGIN (R&D, 6057-NG) in 3-4 µl 0.1% BSA. Tadpoles were anaesthetized with 0.002% MS222, placed on a wet towel, and both right and left hindlimbs were amputated from ankle level in either –restricted or -incompetent tadpoles. 3-4 beads were placed on the amputation plane of the right hindlimb. Left hindlimbs served as an internal control for the experiments."*

We hope the reviewer also agrees these experiments further strengthens the findings in this study.

The latter suggestion of the authors is contradicted in several places throughout the text where we are rightly cautioned against extrapolating across species. There are two extrapolations which stand out to me in reading this work: one, Urodele limb buds do not have an AER as the text points out (Purushothaman et al), although there is no reason why the AER has to be an identifiable multi- layered structure rather than a single layer of cells with specific gene expressions (as in supplemental figure S7);

We completely agree with the reviewer that the AER might not be a multi-layered structure in other species. Based on this comment, we feel it is more appropriate to say that "**Current results suggest** that there is no AER in axolotl". Although we added an equivalent sentence to main text line 588 for this, in our revision we also added a similar comment in the introduction.

In introduction:
Main text line: 95-96
"However, current results suggest that limb regeneration-competent salamanders lack a developmental AER (Purushothaman et al., 2019). "

In discussion:
Main text line: 588-590
"Based on current results, regeneration-competent axolotls are suggested to not have a developmental AER (Purushothaman et al., 2019), but can form AEC."

and two, if Noggin inhibits the AEC why does Noggin application in chick and mouse limb buds promote the AER? I think the latter contradiction should be more rigorously discussed.

We appreciate this comment as we believe it is very critical to acknowledge differences between species. We discussed related differences further in our discussion.

Revised discussion:
Main text line: 570-585
"Furthermore, the pace of chondrogenesis may have an association with limb regeneration ability across species, such that terrestrial warm-blooded animals may have a more robust and fast-paced chondrogenesis program compared to regeneration-competent aquatic cold blooded animals. Indeed, limb regeneration-incompetent species such as chicken or mouse have a faster limb chondrogenesis program during their development compared to regeneration-competent axolotl and Xenopus.
Additionally, although a side-by-side comparative study would be required, mice bone fractures were documented to heal faster compared to axolotl bone fractures (Hutchison et al., 2007; Vortkamp et al., 1998). It is well-established that chondrogenic programs are heavily influenced

© 2021. Published by The Company of Biologists under the terms of the Creative Commons Attribution License (https://creativecommons.org/licenses/by/4.0/).

by BMP pathway activity. The ratio of BMP agonist/antagonist (e.g. BMP4/NOGGIN) during development, injury, or upon limb amputation may be different between limb regeneration-competent and -incompetent animals. This difference may also be connected to observed Noggin phenotypes across species.

Specifically, adding exogenous Noggin results in extended AER maintenance in chicken (Pizette & Niswander, 1999) and mouse (Wang et al., 2004), whilst it abolishes AER in Xenopus (Jones et al., 2013). Targeted comparative studies on these topics will be subject of future work.“

I have only one other comment regarding work previously done on the AEC which has been studied in salamanders for more than 70 years and much less so for Xenopus. In the Intro, lines 86-88 ‘Moreover, it is not clear how a specialized wound epidermis forms during limb regeneration, what are the cell types composing the AEC, what is the broad repertoire of genes expressed by it, and why it cannot form in some instances/species’. This is a very Xenopus-centric view because quite a lot is known about the AEC in axolotls/newts. For example it is known that the early migrating epidermal cells which form the AEC do not divide (Hay & Fishman, 1961, Dev Biol. 3, 26-59) - the authors show this here for Xenopus without commenting about this early data from newts, making their findings less “surprising” (line 276);

All reviewers commented on this topic, and we thank them for raising this issue. We now cite suggested key paper from Hay and Fischman, 1961 and a review paper on wound epidermis by Campbell and Crews, 2008, citing other relevant studies.

As Reviewer 3 also notes, we do not know the properties of *Xenopus* AEC as much as we understand the AEC in salamanders. Additionally, molecular characterization of *Xenopus* limb explants was not done before. Critically, in the aforementioned studies, the specialised wound epidermis was investigated as a *tissue*, without characterizing its specific cell types. Here, we specifically tested if AER cells within specialised wound epidermis *tissue* are formed after cell division. For this, we stained against *Fgf8* mRNA which corresponds to a specific population within the basal layer of AEC tissue, and check for EdU co-stained cells. On another note, we note that our scRNA-Seq data also suggests that AER cells are not very proliferative (Fig S2D). Moreover, not just in salamander AEC, but also mouse AER has been suggested to not proliferate much (Storer et al, Cell, 2013). We edited our text and now also include all this information as well.

Revised texts:
Main text line: 293-318
“Next, we asked how AER cells form on the amputation plane. It has been suggested that salamander AEC tissue forms by migration of epidermal cells to the amputation plane, and does not require cell proliferation (Campbell and Crews, 2008; Hay and Fischman, 1961). Moreover, the mouse AER was previously suggested to be a largely mitotically inactive tissue (Storer et al., 2013). However, it is not known whether similar mechanisms apply to AER cells within the specialised wound epidermis, and also to what extent they are seen in Xenopus. Therefore, we first traced skin tissue located on the edge of explants, and found that they contributed to the covering of both the distal and proximal sites (Figure S13B). As the amputation planes are covered by skin tissue from the surrounding area, we reasoned that AER cells are likely to have originated from skin cells. As amputation eliminates the majority, if not all, of AER cells in the limb, we hypothesized that AER cells are derived from remaining skin stem cells. If AER cells are induced through proliferation and differentiation following amputation, all AER cells should be the product of cell division. To test this hypothesis, we assayed the level of EdU incorporation (labelling newly synthesized DNA, hence divided cells) in newly-formed AER cells, using Fgf8 positivity to specifically identify AER cells within the AEC tissue. We found that only ~40% of AER cells (distal epidermal Fgf8+) were EdU positive at 3 dpa (Figure S13C), suggesting that most AER cells are induced independently of cell division following amputation. These results parallel our transcriptomics-based cell-cycle assessment in which AER cells display low levels of proliferation (Figure S2D). Using the transcriptomics data, we identified a stepwise activation of Lgr5.S (a WNT target gene) followed by Fgf8.L expression as a possible gene-expression trajectory that could allow basal epidermal cells to convert directly to AER cells without cell division (Figure 3D). Consistent with such a process, when visualized in vivo, we found that Fgf8+/Lgr5+ AER cells were

© 2021. Published by The Company of Biologists under the terms of the Creative Commons Attribution License (https://creativecommons.org/licenses/by/4.0/).

flanked by Lgr5+ cells in the basal epidermis on the amputation plane or in the developing limb (Figures 3E and S7A-B). Overall, these results support the hypothesis that basal epidermal cells can acquire AER cell identity without cell division."

a microarray of the AEC has been done in axolotls (Monaghan et al., 2012) identifying >1000 genes; many transcription factors are expressed including sp9 which is thought to be crucial for its functioning; the secreted molecules Wnt5a, 5b, and several Fgfs are expressed; several Mmps and extracellular matrix molecules are expressed. This seems to be a "broad repertoire of genes"!

We thank the reviewer for this comment. In the Monaghan et al study referred to by the reviewer, and in other RNA-Seq and similar expression analysis approaches (Pearl et al BMC Dev Bio 2008, Campbell et al Dev Dynamics 2011, Knapp et al Plos ONE 2013, Tsai et al Development 2019, Tsai et al Elife 2020), experiments were performed to determine genes expressed in AEC *tissue* via bulk-approaches. Hence, these studies were not able distinguish which potential cell types are present in tissues and would include gene expression from cells that are basal epidermal but not AER cells. Here, by using single-cell transcriptomics, we specifically catalogued expressed genes in AER cells within specialised wound epidermis *tissue*. Nonetheless, we agree that this single-cell versus tissue difference and in general more Xenopus-centric statements should have been written more clearly. Hence, we have edited the text accordingly.

Revised introduction
Main text line 72-75
"Amphibian limb regeneration relies on a specialized wound epidermis (also known as the apical-epithelial-cap, AEC) that forms on the amputation plane and has been characterized primarily as a tissue in regenerating salamander limbs (Campbell et al., 2011; Campbell and Crews, 2008; Knapp et al., 2013, p. 2013; Monaghan et al., 2012; Pearl et al., 2008; Tsai et al., 2020, 2019).."

We also deleted sentences involving "broad repertoire of genes". Instead we now write:

Revised introduction
Main text line 87-89
"However, it remains largely unclear which cell types within AEC tissue are critical for regeneration, which transcriptional and functional properties are associated with a mature AEC and regeneration, and why the AEC cannot form or maturate in some instances/species.."

I think at least Hay & Fishman should be referred to because the authors do the very same experiment here (although not with tritiated thymidine, admittedly).

Thanks for the comment. We now cite this paper, and have edited our text as described in this letter, rebuttal letter lines 1241-1254.

There is a ton of other great work here, too much to mention, but I particularly liked the comparison of the AEC with the tail regenerating epithelium form previous work identifying ROCs and also the description of fibroblast changes and their dedifferentiation at different regeneration competent/incompetent stages.

We thank the reviewer for their support, especially for acknowledging differences among species!

Second decision letter

MS ID#: DEVELOP/2020/199158

MS TITLE: Secreted inhibitors drive the loss of regeneration competence in Xenopus limbs

AUTHORS: Benjamin David Simons, Jerome Jullien, John Marioni, John Gurdon, Can Aztekin, and Tom Hiscock

© 2021. Published by The Company of Biologists under the terms of the Creative Commons Attribution License (https://creativecommons.org/licenses/by/4.0/).

I have now received all the referees reports on the above manuscript, and have reached a decision. The referees' comments are appended below, or you can access them online: please go to BenchPress and click on the 'Manuscripts with Decisions' queue in the Author Area.

The overall evaluation is positive and we would like to publish a revised manuscript in Development, provided that the comments of reviewer 2 can be satisfactorily addressed. Please attend to the comments in your revised manuscript and detail them in your point-by-point response. If you do not agree with any of their criticisms or suggestions explain clearly why this is so.

We are aware that you may currently be unable to access the lab to undertake experimental revisions. If it would be helpful, we encourage you to contact us to discuss your revision in greater detail. Please send us a point-by-point response indicating where you are able to address concerns raised (either experimentally or by changes to the text) and where you will not be able to do so within the normal timeframe of a revision. We will then provide further guidance. Please also note that we are happy to extend revision timeframes as necessary.

Reviewer 1

Advance summary and potential significance to field

Clearly there was a lot to discuss between the reviewers and authors. Many of the same concerns were raised by each reviewer, but the authors clearly put a lot of thought and work in their revisions. Overall, the revisions clear up many of the concerns I originally had with the manuscript.

Comments for the author

No further changes are requested.

Reviewer 2

Advance summary and potential significance to field

In this revision, the authors have made a seriously effort to incorporate all of the suggestions and address the many concerns of the reviewers. The paper is now a better reflection of what is known, and what is unknown in the field, with more nuanced discussion in the introduction and discussion, and inclusion of more references. They have tempered claims throughout the manuscript, as called out by reviewers, that were previously overstatements. They have also now performed a few new experiments, which is key to advancing their main claims that in the tadpole limb regeneration system, inhibitory molecules secreted by chondrocytes may be driving some aspects of regenerative loss. Overall, the paper is better and will spark discussion while advancing an interesting hypothesis for why limb regeneration becomes restricted in Xenopus as the animals mature, which could inform the question in mammals as well.

Comments for the author

There are still a few areas that would benefit from a bit more revising to make the paper more clear:
—Reviewer 1 noted that the experiments point to the conclusion that these factors are “likely inhibitory to patterning, not blastema formation.” This is an excellent point, but it seems the authors mistook the point to be asking for experiments explicitly directed to address patterning (which they say would be another study altogether). However, I interpreted this critique to mean that the authors should back away from claims that the results support a model in which these inhibitory factors prevent blastema formation, but rather embrace a model in which they are more likely inhibiting downstream events, like patterning.

© 2021. Published by The Company of Biologists under the terms of the Creative Commons Attribution License (https://creativecommons.org/licenses/by/4.0/).

—The new experiments applying anti-Noggin antibodies in vivo are good and support the idea that these effects are truly biologically meaningful outside of a culture dish, even if the effects observed were small. I also like that they incorporated FGF-10 into the experiment, which links to the important earlier work published by another group, and further supports their model.
—I do think that the authors should include the "AER genes" analysis from the stage 60 tadpoles, as discussed in response to Reviewer 1. I think doing this will support their claim that at stage 60, even thought Tp63+ cells are found, they don't express the "AER genes." This is important, and readers can handle the nuances.

—Of special note, they finally got the Hay and Fischman 1961 paper in there, which would absolutely be expected to be referenced and discussed in a paper on this topic. However, neither that study nor the Campbell one actually blocked proliferation and showed the AEC still forms—the conclusions in Hay & Fischman are based on labeling of cells undergoing DNA synthesis and are therefore correlative but not functional. So they might not want to present the reference in a way that leads the reader to think that these two papers might have actually blocked cellular proliferation and observed AEC formation nonetheless.

—Relatedly, the conclusion that "basal epidermal cells can acquire AER identity without cell division" is murky. The fact that only 40% of Fgf8+ cells were EdU+ at three days does not support this conclusion one way or the other. I think they should temper this conclusion because they simply have not done a functional experiment—like one that would block proliferation and show the cells still acquire AEC identity—to back it up. I am not suggesting they do that experiment now, but rather that they do not state the data as supporting that hypothesis that "basal epidermal cells can acquire AER identity without cell division"—instead, they can say "While we favor the hypothesis that basal epidermal cells can acquire AER identity without cell division our data cannot rule out that cell division may be coupled with acquisition of AER identity."

—Follow up to Reviewer 1 and the proximal vs. distal debate: definitely this should be qualified. They made a good start with this revision, but "Nonetheless, FGF10 treatment was not sufficient to induce strong Fgf8 expression at the proximal site of explants (Figure S14C), which may be, at least in part, due to abundant antagonist cues" is still an overstatement. How about: "Nonetheless, FGF10 treatment was not sufficient to induce strong Fgf8 expression at the proximal site of explants (Figure S14C), which we hypothesize could be at least in part, attributable to differences in abundance of proposed antagonist cues."

—"Noggin cancels the positive effect of FGF10 application"—should probably be "attenuates" or similar.

—We still don't have functional data demonstrating the proposed equivalence of regeneration-used "AER" cells and AER cells in development. However, the authors have done a better job couching their decision in the revision, and they have performed additional comparative analyses (S6 and sup. table 2), so at least readers can better decide for themselves. I would still favor calling the ones that appear during initial development "AER cells" (as has always been the case) and calling these "AER-like cells." It is the sounder conclusion, but it also carries a meaning that I think actually supports their cause—the "-like" part implies there is some difference, and while they don't want to focus on transcriptional differences, which they claim/show are minor, the "-like" part may just as well be referring to the fact that they do appear later, and in response to an injury! "-like" takes the place of the more cumbersome "-reminiscent" and eliminates the need to get into the weeds about cell type and what one really means by that.

—The authors state in their response, "However, only AER cells can secrete all of these ligand families altogether and at a very high level which, in our opinion, makes them a signalling centre." It would be helpful if they would make that more clear in several places in the manuscript, their choice where, and if they don't want to, that's fine, but I think this is a critical point and can be missed in reading the paper.

—I still think a properly controlled study (with shams etc.) where they ablated or removed the skeletal element and either tested in vivo or in a subsequent explant assay would really provide a lot more evidence that the differentiation of skeletal elements underlies the inhibition of limb

© 2021. Published by The Company of Biologists under the terms of the Creative Commons Attribution License (https://creativecommons.org/licenses/by/4.0/).

regeneration. They don't want to do this (here), presumably because they think it's beyond the scope of this article, but it would certainly advance the overall model.

Reviewer 3

Advance summary and potential significance to field

Understanding of the relation between the developing limb epithelium (AER) and the regenerating limb epithelium (AEC or AC), why Xenopus fails to regenerate at later stages of development, possibilities for inducing limb regeneration in the future.

Comments for the author

The authors responded satisfactorily to my previous comments and have thus improved the paper significantly (it was already a great paper).

Second revision

Author response to reviewers' comments

Reviewer 1 Advance Summary and Potential Significance to Field:
Clearly there was a lot to discuss between the reviewers and authors. Many of the same concerns were raised by each reviewer, but the authors clearly put a lot of thought and work in their revisions. Overall, the revisions clear up many of the concerns I originally had with the manuscript.

Reviewer 1 Comments for the Author:
No further changes are requested.

Reviewer 2 Advance Summary and Potential Significance to Field:
In this revision, the authors have made a seriously effort to incorporate all of the suggestions and address the many concerns of the reviewers. The paper is now a better reflection of what is known, and what is unknown, in the field, with more nuanced discussion in the introduction and discussion, and inclusion of more references. They have tempered claims throughout the manuscript, as called out by reviewers, that were previously overstatements. They have also now performed a few new experiments, which is key to advancing their main claims that in the tadpole limb regeneration system, inhibitory molecules secreted by chondrocytes may be driving some aspects of regenerative loss. Overall, the paper is better and will spark discussion while advancing an interesting hypothesis for why limb regeneration becomes restricted in Xenopus as the animals mature, which could inform the question in mammals as well.

We thank the reviewer for their constructive and insightful comments, which have significantly improved the manuscript and made it more precise.

Reviewer 2 Comments for the Author:
There are still a few areas that would benefit from a bit more revising to make the paper more clear:

—Reviewer 1 noted that the experiments point to the conclusion that these factors are "likely inhibitory to patterning, not blastema formation." This is an excellent point, but it seems the authors mistook the point to be asking for experiments explicitly directed to address patterning (which they say would be another study altogether). However, I interpreted this critique to mean that the authors should back away from claims that the results support a model in which these inhibitory factors prevent blastema formation, but rather embrace a model in which they are more likely inhibiting downstream events, like patterning

© 2021. Published by The Company of Biologists under the terms of the Creative Commons Attribution License (https://creativecommons.org/licenses/by/4.0/).

This is an interesting idea and a topic we are very much interested in. However, we disagree that we *"should back away from claims that the results support a model in which these inhibitory factors prevent blastema formation"*. This is because, neither in our original submission nor our revised manuscript, do we claim that secreted inhibitors affect blastema formation or patterning. Reviewer 1 suggested that secreted factors may be affecting patterning but not blastema formation. Although this is an interesting hypothesis, as discussed in the rebuttal letter in response to Reviewer 1, our experiments cannot provide insight into this topic.

Here, we focused on AER cell formation at the initial stages after limb amputations. Our ex vivo work is limited to 3 days post-amputation. Likewise, our in vivo experiments are limited with bead applications, which we expect to only affect the first days after amputation. Meanwhile, blastema formation corresponds to 5-10 days post-amputation, and subsequent events, such as blastema patterning, occur at much later time points. Hence, we feel that neither our experiments nor our model sheds much light on the role of secreted factors on blastema formation or patterning; this should be the subject of future work. We hope our response clarifies this issue.

—The new experiments applying anti-Noggin antibodies in vivo are good and support the idea that these effects are truly biologically meaningful outside of a culture dish, even if the effects observed were small. I also like that they incorporated FGF-10 into the experiment, which links to the important earlier work published by another group, and further supports their model.

We thank the reviewer for their comment and agree that the new experiments draw more concrete parallels between ex vivo and in vivo!

—I do think that the authors should include the "AER genes" analysis from the stage 60 tadpoles, as discussed in response to Reviewer 1. I think doing this will support their claim that at stage 60, even thought Tp63+ cells are found, they don't express the "AER genes." This is important, and readers can handle the nuances.

Thanks for the comment. We have now included these data in Figure S6 and mentioned in the text as below: Revised main-text line 226-227:
"In parallel, AER cell associated ligand expressions were lower or absent in regeneration-incompetent *tp63*+ epidermal cells **(Figure S6E)**."

Also included the figure legend to Figure S6 as follows:
"**(e)** Dot plot showing expression of AER cell associated selected ligands for TP63+ epidermal cells during development and at 5 dpa in regeneration-competent, -restricted, and -incompetent samples. Dot color indicates mean expression; dot size represents the percentage of cells with non-zero expression."

—Of special note, they finally got the Hay and Fischman 1961 paper in there, which would absolutely be expected to be referenced and discussed in a paper on this topic. However, neither that study nor the Campbell one actually blocked proliferation and showed the AEC still forms—the conclusions in Hay & Fischman are based on labeling of cells undergoing DNA synthesis and are therefore correlative but not functional. So they might not want to present the reference in a way that leads the reader to think that these two papers might have actually blocked cellular proliferation and observed AEC formation nonetheless.

Thanks a lot for noting this! Indeed, our revised sentence could lead to misunderstandings. We now changed the sentence (main-text line 299) below:
"Next, we asked how AER cells form on the amputation plane. It has been suggested that salamander AEC tissue forms by migration of epidermal cells to the amputation plane, and does not require cell proliferation."

To

"Next, we asked how AER cells form on the amputation plane. It has been suggested that salamander AEC tissue forms by migration of epidermal cells to the amputation plane, and **may** not require cell proliferation."

© 2021. Published by The Company of Biologists under the terms of the Creative Commons Attribution License (https://creativecommons.org/licenses/by/4.0/).

—Relatedly, the conclusion that “basal epidermal cells can acquire AER identity without cell division” is murky. The fact that only 40% of Fgf8+ cells were EdU+ at three days does not support this conclusion one way or the other. I think they should temper this conclusion because they simply have not done a functional experiment—like one that would block proliferation and show the cells still acquire AEC identity—to back it up. I am not suggesting they do that experiment now, but rather that they do not state the data as supporting that hypothesis that “basal epidermal cells can acquire AER identity without cell division”—instead, they can say “While we favor the hypothesis that basal epidermal cells can acquire AER identity without cell division, our data cannot rule out that cell division may be coupled with acquisition of AER identity.”

Absolutely! Thanks for this comment. We agree with the reviewer that the functional consequences of cell division should be tested in the future. Hence, we now changed the below sentence to:

Revised main-text line 321-323:

“Overall, these results support the hypothesis that basal epidermal cells can acquire AER cell identity without cell division, although understanding the functional relevance of cell division on AER cell fate requires further work.”

—Follow up to Reviewer 1 and the proximal vs. distal debate: definitely this should be qualified. They made a good start with this revision, but “Nonetheless, FGF10 treatment was not sufficient to induce strong Fgf8 expression at the proximal site of explants (Figure S14C), which may be, at least in part, due to abundant antagonist cues” is still an overstatement. How about: “Nonetheless, FGF10 treatment was not sufficient to induce strong Fgf8 expression at the proximal site of explants (Figure S14C), which we hypothesize could be, at least in part, attributable to differences in abundance of proposed antagonist cues.”

Thanks for this comment. We now changed our sentence as the reviewer suggests.

—“Noggin cancels the positive effect of FGF10 application”—should probably be “attenuates” or similar.

We agree that “attenuates” reflect the phenotype better. We changed our sentence according to the reviewer comment.

Revised main-text line 513:

“Moreover, we demonstrated that NOGGIN **attenuates** the positive effect of FGF10 application, further highlighting the downstream role played by the secreted inhibitors.”

—We still don’t have functional data demonstrating the proposed equivalence of regeneration-used “AER” cells and AER cells in development. However, the authors have done a better job couching their decision in the revision, and they have performed additional comparative analyses (S6 and sup. table 2), so at least readers can better decide for themselves. I would still favor calling the ones that appear during initial development “AER cells” (as has always been the case) and calling these “AER-like cells.” It is the sounder conclusion, but it also carries a meaning that I think actually supports their cause—the “-like” part implies there is some difference, and while they don’t want to focus on transcriptional differences, which they claim/show are minor, the “-like” part may just as well be referring to the fact that they do appear later, and in response to an injury! “-like” takes the place of the more cumbersome “-reminiscent” and eliminates the need to get into the weeds about cell type and what one really means by that.

Thanks for this comment. We already discussed our reasoning for the lack of functional equivalence assay in our rebuttal letter. Unfortunately, as discussed before in our rebuttal letter, we do not think we can perform such an experiment with current methodologies and within a reasonable time frame. We hope to proceed with such investigations in the future.

We thank the reviewer for giving us a chance to us explain our reasoning in detail and appreciate the comment “*the authors have done a better job couching their decision in the revision, and they*

© 2021. Published by The Company of Biologists under the terms of the Creative Commons Attribution License (https://creativecommons.org/licenses/by/4.0/).

have performed additional comparative analyses (S6 and sup. table 2), so at least readers can better decide for themselves.”. With the changes during the revision, we agree with the reviewer that the current state of the manuscript is more evident on the points of similarity/differences between developmental and regeneration-associated AER cells. As the reviewer also noted, with the revised version, readers can decide the naming themselves.

As discussed before in our rebuttal letter, we see only **one** Fgf8+ epidermal population in the single-cell dataset. Naming one cluster with two names creates confusion for people from regeneration or developmental biology fields and people from different disciplines such as computational biology. Due to this, we still favour naming this cluster as AER cells for readership purposes while maintaining scientific accuracy as we described what we mean by AER cells in the manuscript.

—The authors state in their response, “However, only AER cells can secrete all of these ligand families altogether and at a very high level which, in our opinion, makes them a signalling centre.” It would be helpful if they would make that more clear in several places in the manuscript, their choice where, and if they don’t want to, that’s fine, but I think this is a critical point and can be missed in reading the paper.

This is a great suggestion! We now include a statement related to this point:

“In our dataset, we found that different populations express ligands from different major signalling pathways (FGF, BMP, WNT, DELTA, TGF-β) **(Figures S6C)**. However, only AER cells can express multiple ligands from these gene families altogether and at a very high level, making them a highly potent signalling centre **(Figures 2E, S6)**.”

—I still think a properly controlled study (with shams etc.) where they ablated or removed the skeletal element and either tested in vivo or in a subsequent explant assay would really provide a lot more evidence that the differentiation of skeletal elements underlies the inhibition of limb regeneration. They don’t want to do this (here), presumably because they think it’s beyond the scope of this article, but it would certainly advance the overall model.

As discussed in the rebuttal letter, we agree with the reviewer that an experiment targeting the role of chondrogenic cells on regeneration with cell ablation methods would be an excellent direct assay testing our model.

Unfortunately, as discussed before in our rebuttal letter, we do not think we can perform a controlled experiment with our current methodologies and within a reasonable time frame. We hope to proceed with such experiments in the future.

Reviewer 3 Advance Summary and Potential Significance to Field:
Understanding of the relation between the developing limb epithelium (AER) and the regenerating limb epithelium (AEC or AC), why Xenopus fails to regenerate at later stages of development, possibilities for inducing limb regeneration in the future.

Reviewer 3 Comments for the Author:
The authors responded satisfactorily to my previous comments and have thus improved the paper significantly (it was already a great paper).

Third decision letter

MS ID#: DEVELOP/2020/199158

MS TITLE: Secreted inhibitors drive the loss of regeneration competence in Xenopus limbs

© 2021. Published by The Company of Biologists under the terms of the Creative Commons Attribution License (https://creativecommons.org/licenses/by/4.0/).

AUTHORS: Benjamin David Simons, Jerome Jullien, John Marioni, John Gurdon, Can Aztekin, and Tom Hiscock

ARTICLE TYPE: Research Article

I am satisfied with the response to the review and the revision of the manuscript (please see the appended Editor's note). Your manuscript has been accepted for publication in Development, pending our standard ethics checks.

© 2021. Published by The Company of Biologists under the terms of the Creative Commons Attribution License (https://creativecommons.org/licenses/by/4.0/).
